# Synthesis and *h*LDHA Inhibitory Activity of New Stiripentol-Related Compounds of Potential Use in Primary Hyperoxaluria

**DOI:** 10.3390/ijms252413266

**Published:** 2024-12-10

**Authors:** Mario Rico-Molina, Juan Ortega-Vidal, Juan Molina-Canteras, Justo Cobo, Joaquín Altarejos, Sofía Salido

**Affiliations:** 1 Department of Inorganic and Organic Chemistry, Faculty of Experimental Sciences, University of Jaén, Campus of International Excellence in Agri-Food (ceiA3), 23071 Jaén, Spain; mrico@ujaen.es (M.R.-M.); or juan.ortegavidal@universityofgalway.ie (J.O.-V.); jcantera@ujaen.es (J.M.-C.); jcobo@ujaen.es (J.C.); ssalido@ujaen.es (S.S.); 2 School of Biological and Chemical Sciences, Ryan Institute, University of Galway, H91 TK33 Galway, Ireland

**Keywords:** *h*LDHA inhibitors, primary hyperoxaluria, stiripentol analogues, resolution of racemates, chiral HPLC, absolute configuration, Mosher’s method, electronic circular dichroism

## Abstract

Human lactate dehydrogenase A (*h*LDHA) is a homotetrameric isozyme involved in the conversion of glyoxylate into oxalate in the cytosol of liver cells (hepatocytes) and partially responsible for the overproduction of oxalate in patients with the rare disease called primary hyperoxaluria (PH). Recently, *h*LDHA inhibition has been validated as a safe therapeutic method to try to control the PH disease. Stiripentol (STP) is an approved drug used in the treatment of seizures associated with Dravet’s syndrome (a severe form of epilepsy in infancy) which, in addition, has been drawing interest in recent years also for potentially treating PH, due to its *h*LDHA inhibitory activity. In this work, several new STP-related compounds have been synthesized and their *h*LDHA inhibitory activity has been compared to that of STP. The synthesis of these analogues to STP was accomplished using crossed-aldol condensation guided by lithium enolate chemistry and a successive regioselective reduction of the resulting α,β-unsaturated ketones. The target molecules were obtained as racemates, which were separated into their enantiomers by chiral HPLC. The absolute configurations of pure enantiomers were determined by the modified Mosher’s method and electronic circular dichroism (ECD) spectroscopy. For the inhibitory effect over the *h*LDHA catalytic activity, a kinetic spectrofluorometric assay was used. All the new synthesized compounds turned out to be more active at 500 μM (46–72% of inhibition percentage) than STP (10%), which opens a new line of study on the possible capacity of these analogues to reduce urinary oxalate levels in vivo more efficiently.

## 1. Introduction

Stiripentol (STP) is an aromatic allylic alcohol (4,4-dimethyl-1-[3,4-[methylenedioxy]-phenyl]-1-penten-3-ol) with a 1,3-benzodioxole ring moiety that has been shown to be useful against Dravet’s syndrome (DS) [1,2]. This rare genetic disease was described by Dr. Dravet in 1978 as a severe myoclonic epilepsy in infancy [3]. DS is a drug-resistant form of developmental and epileptic encephalopathy which arises during the first year of life, with seizures often triggered by fever, infections, or vaccinations, and characterized by intellectual disability and behavioural, sleep, and motor problems [4]. Current treatments for DS are based on a combination of several drugs such as valproate, clobazam, fenfluramine, cannabidiol, and STP [5]. STP was first synthesized in 1973 [6] and authorized by the European Medicines Agency (EMA) in 2007 as an orphan drug for the treatment of DS, followed by its approval in Japan and Canada in 2012 and in the USA in 2018 [7]. Several mechanisms of action have been described that may explain its specific effect on seizures: it being a potentiator of gamma-aminobutyric acid neurotransmission, blockade of voltage-gated sodium and T-type calcium channels, inhibitor of several cytochrome P450 enzymes, and also an inhibitor of the neoglucogenesis enzyme named lactate dehydrogenase (LDH), which is highly activated during seizures [8]. The latter enzyme (LDH) connects STP with another rare disease named primary hyperoxaluria (PH).

Primary hyperoxalurias (PHs) are rare disorders caused by the deficiency of key enzymes involved in the hepatic metabolism of glyoxylate [9,10]. PHs are characterized by an abnormal accumulation of glyoxylate that leads to a nocive overproduction of oxalate in the liver, a reaction catalyzed by the hepatic enzyme lactate dehydrogenase (LDH). According to the defective enzyme (encoded by the corresponding mutated gene) there are different types of PHs, referred to as PH1 (alanine-glyoxylate aminotransferase, AGT) [11], PH2 (glyoxylate/hydroxypyruvate reductase, GRHPR) [12], and PH3 (4-hydroxy-2-oxoglutarate aldolase 1, HOGA1) [13]. In all of them, the accumulated oxalate crystallizes in kidneys as calcium oxalate and produces a deterioration in renal function. This leads to end-stage renal disease (ESRD) and to systemic oxalosis that damages other tissues. PHs are severe diseases that deeply deteriorate the quality of life of patients, requiring, in the final stages of the disease, the practice of liver and kidney transplantations [14]. Primary hyperoxaluria type 1 (PH1) is the most common and severe of the PHs, due to mutations in the *AGXT* gene (coding for AGT), with a high incidence of kidney failure as early as infancy [15]. Traditionally, PH1 has been treated through conservative strategies, such as hyperhydration, crystallization inhibitors (e.g., potassium citrate) and pyridoxine (an AGT co-factor) to slow disease progression, until Lumasiran was approved by the EMA and the U.S. Food and Drug Administration (FDA) in 2020 as an orphan drug for PH1 [16]. Lumasiran has been the first pharmacological treatment for PH1 and is a small interfering ribonucleic acid (siRNA) that silences the messenger ribonucleic acid (mRNA) of the enzyme glycolate oxidase (GO), which catalyzes the transformation of glycolate into glyoxylate. Lumasiran binds to the GO mRNA, thus inducing its degradation and preventing the synthesis of the enzyme, which consequently reduces the conversion of glycolate into glyoxylate. In other words, the mechanism of action of lumasiran inhibits oxalate synthesis by silencing the expression of the GO-encoding gene with RNA interference (RNAi) therapy, thereby reducing oxalate levels in urine and plasma [17]. More recently, another siRNA, named nedosiran, received its first approval in September 2023 in the USA to lower urinary oxalate levels in children aged ≥ 9 years and adults with PH1 and relatively preserved kidney function [18]. Nedosiran targets the degradation of the mRNA of the hepatic enzyme LDH, thereby inhibiting its expression and, as a consequence, reducing oxalate overproduction in all types of PH patients [19]. However, despite the medical progress that these two bio-drugs clearly represent in the treatment of PH patients, it must be pointed out that they have some drawbacks (high cost, injectable administration, and requiring the use of genetic material) that might be overcome with the discovery of conventional small-molecule drugs. In this other field of interest (the substrate reduction strategy), STP could have a chance.

Lactate dehydrogenase (LDH) is an enzyme in the family of 2-hydroxyacid oxidoreductases that can be found in almost all living organisms. It is mainly involved in the catalysis of the reduction of pyruvate into lactate at the end of the glycolytic process (lactate fermentation), coupled to the oxidation of cofactor NADH to NAD^+^ (reversible process) [20]. Human LDH (*h*LDH) has a tetrameric structure composed of an association of two types of subunits (H-type and M-type) that gives up to five different isoforms (isozymes): *h*LDH-1, *h*LDH-2, *h*LDH-3, *h*LDH-4, and *h*LDH-5. Homotetramers *h*LDH-1 (also named *h*LDHB; four H-type subunits) and *h*LDH-5 (also named *h*LDHA; four M-type subunits) are primarily found in the heart and brain, and in skeletal muscle and liver, respectively [21]. The latter isozyme, *h*LDHA, is not only involved in the well-known reversible conversion of pyruvate into lactate, but also in the conversion of glyoxylate into oxalate in the cytosol of liver cells (hepatocytes) [22]. This *h*LDHA is the hepatic enzyme LDH, mentioned above, whose expression is diminished by the siRNA nedosiran, leading to a reduction in oxalate levels in PH patients [18,19]. The involvement of *h*LDHA in the last step of endogenous oxalate production, together with the efficacy of nedosiran, confirms the rationale for developing PH treatments based on the inhibition of *h*LDHA by small molecules [23]. To date, there are no approved treatments for PH using conventional small-molecule drugs. Treatments with chemically stable small drugs would ensure low-cost scale-up processes, non-demanding conservation protocols, and easier oral administration. In that sense, STP could be a good candidate for becoming a new drug for PH in the future, since it was able to inhibit *h*LDHA in vitro [24], to decrease *h*LDHA-mediated oxalate synthesis by hepatocytes in vitro, and to reduce urine oxalate excretion in rats [25]. In addition, Letavernier’s group administered STP to two young PH1 patients, resulting in a significant reduction in urinary oxalate levels [25]. Later, Boyer’s group dispensed STP in combination with standard supportive care to a 21-month-old female with PH1 (with preserved kidney function), finding that urinary oxalate levels were normalized in a few weeks and the kidney stones had disappeared [26]. On the other hand, STP failed to lower plasma oxalate in a dialysis-dependent PH1 patient [27] and two further PH1 patients with chronic kidney disease and early ESRD [28]. It seems to suggest that only PH patients with preserved renal function in the early stages will benefit from STP. Nonetheless, larger-scale studies are required to validate this conclusion and, in the meantime, the results of an open-label phase 2 study of STP (already completed) are awaited [29], with the phase 3 study having just started in August 2024 [30]. In any case, efforts must be made continuously in all therapies in progress to try to solve the health problems of PH patients [31].

**Figure 1:**
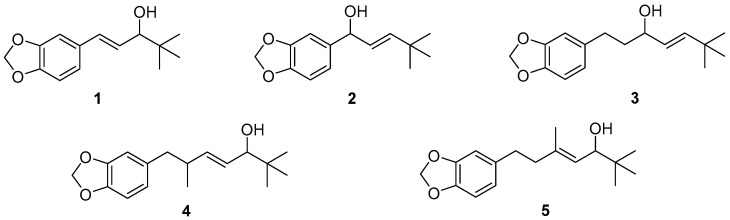
Structure of the compounds to be synthesized in this work.

Due to the interest and expectations generated around STP, we have proposed, in the present work, the evaluation of the inhibitory activity of both STP enantiomers [(+)-(*R*)-**1**, (−)-(*S*)-**1**)] against *h*LDHA (first time to be measured) and that of several novel STP-related compounds with the hope of finding better inhibitory activities against *h*LDHA than those of STP (Figure 1). Compound **2** is a regioisomer of STP (**1**) and was a new compound when this work began, until it was recently synthesized [32]. Compound **3** is a homologous structure of **2** and compounds **4** and **5** are homologous structures of **1**. This work is part of a project that we have been developing in recent years aimed at finding new *h*LDHA inhibitors as a potential therapy against PH [33,34,35,36,37,38,39]. It should also be noted that the search for new *h*LDHA inhibitors is of great interest to the scientific community, not only because of its relationship with PH [40,41,42] but also because of its role in the control of invasive tumour cells [42,43,44,45].

## 2. Results and Discussion

### 2.1. Synthesis of Stiripentol (STP, ***1***) and Iso-Stiripentol (Iso-STP, ***2***)

**Scheme 1:**
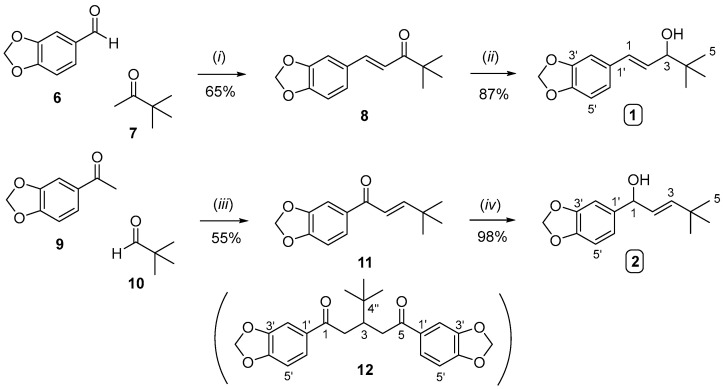
Synthesis of stiripentol (**1**) and iso-stiripentol (**2**). Reagents and conditions: (*i*) KOH, MeOH-H_2_O, 75 °C; (*ii*) NaBH_4_, MeOH, r.t.; (*iii*) KOH, DMSO, 11–13 °C; (*iv*) NaBH_4_/CeCl_3_, DCM-MeOH, 0 °C.

STP (**1**) was synthesized by Claisen–Schmidt condensation of commercially available piperonal (**6**) and pinacolone (**7**) to afford the α,β-unsaturated ketone **8**, which was selectively reduced with sodium borohydride (NaBH_4_) to give racemic STP ((±)-**1**) (Scheme 1). The experimental procedure followed is based on that described in the literature [46] and the nuclear magnetic resonance (NMR) data of compounds **8** and **1** agree with those reported in that work.

Regarding iso-STP (**2**), its synthesis was designed in two steps, following the approach used in the synthesis of STP (**1**). That is, in a first step, the aldol condensation of acetopiperone (**9**) and pivalaldehyde (**10**) was carried out (Scheme 1). When this reaction was performed, as for the synthesis of **8** (using potassium hydroxide in a mixture of methanol-water at room temperature), the α,β-unsaturated ketone **11** was obtained in ca. 21% yield along with the compound resulting from the conjugate addition of a molecule of methanol to the C-3 position of ketone **11** (ca. 5% yield). In order to prevent the formation of this by-product and increase the amount of **11**, the use of methanol was avoided, trying with polar aprotic solvents and strong bases as recommended in the literature [47]. Thus, the reaction of **9** and **10** with potassium hydroxide in dimethylsulfoxide at 11–13 °C yielded a crude reaction composed mainly of ketone **11**, which was purified by column chromatography. The NMR and high-resolution mass spectrometry (HRMS) data of compound **11** allowed us to easily establish its structure. Compound **11** is a new compound and is the regioisomer of ketone **8** (Scheme 1). Moreover, in several reactions achieved with **9** and **10**, small amounts of the side product **12** were obtained. This compound was also purified by column chromatography and its structure was established by NMR spectroscopy and mass spectrometry. Compound **12** is also a new compound, and its formation can be justified by the conjugate addition of the enolate of compound **9** to the C-3 position of ketone **11**. It should be noted that the reaction temperature of the aldol condensation of **9** and **10** is crucial to minimize the formation of **12**, since its presence increases when the reaction is accomplished at room temperature.

Once ketone **11** was properly obtained, it was reacted with sodium borohydride in methanol at room temperature for 20 h following the same protocol to obtain (±)-**1** from ketone **8**. However, the reaction from **11** was not as selective as from **8**, yielding a mixture of compound **2** and the compound with the whole α,β-unsaturated carbonyl system reduced in a 1.00:0.36 ratio (according to ^1^H NMR). In order to avoid the presence of that side product, ketone **11** was reacted with NaBH_4_ and cerium chloride (CeCl_3_) (Luche reduction) in methanol at room temperature for 20 min giving a good yield of compound **2** with only a small amount of the side product (1.00:0.06 ratio). Several attempts were made to further improve the selectivity of the Luche reduction on **11** since it was observed that iso-STP (**2**) is not stable during its column chromatography purification on silica gel (also not in HPLC purifications; see Figure S43 in the Supplementary Materials) leading to the partial isomerization into **1**. The relative instability of **2** is a consequence of the secondary OH in allylic and benzylic positions and chromatographic purifications should be avoided. Thus, the selectivity of the reaction was further increased when the Luche reduction on **11** was accomplished in a mixture of dichloromethane–methanol at 0 °C, yielding, in just 1 min, pure racemic iso-STP ((±)-**2**) (1.00:0.03 ratio) (Scheme 1). Compound **2** was recently synthesized by a Ni-catalyzed reductive coupling of piperonal (**6**) and 3,3-dimethyl-1-butyne [32] and its NMR data were in agreement with those reported in that work.

### 2.2. Synthesis of Compound ***3*** (Analogue to Iso-STP) and Compounds ***4*** and ***5*** (Analogues to STP)

Once compounds **1** and **2** were synthesized, the syntheses of three novel analogues of them (**3**–**5**) were designed (Figure 1). These compounds maintain the 1,3-benzodioxole ring moiety and the *tert*-butyl group of STP (**1**) and iso-STP (**2**), but have two or three additional carbon atoms between the aromatic ring and the allylic alcohol subunit. With these compounds, **3**–**5**, we tried to find out if an increase in the distance between the end moieties of **1** and **2** would have a positive impact on the inhibitory activity of **3**–**5** with respect to **1** and **2**. Furthermore, in the case of compound **3** we sought to give stability to said compound by moving the OH group of the iso-STP (**2**) from both benzylic and allylic positions to a non-benzylic location.

**Scheme 2:**
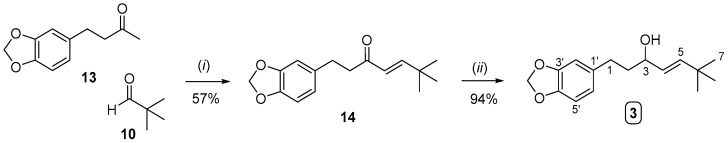
Synthesis of compound **3**, an analogue to iso-stiripentol (**2**). Reagents and conditions: (*i*) (a) LDA, THF, −80 °C; (b) *p*-TsOH, DCM, r.t.; (*ii*) NaBH_4_/CeCl_3_, DCM-MeOH, 0 °C.

The synthesis of compound **3** was first attempted by aldol condensation of piperonyl acetone (**13**) and pivalaldehyde (**10**) with KOH in a mixture of methanol–water at different temperatures, following a similar protocol to that used in the synthesis of STP (**1**). However, a fraction of the starting materials remained unreacted and the column chromatography of the crude reaction yielded small amounts of the α,β-unsaturated ketone **14** (Scheme 2). In order to avoid the formation of side products in the reaction between **13** and **10**, a preformed lithium enolate of ketone **13** was prepared by a reaction of **13** with lithium diisopropylamide (LDA) at a low temperature following a standard protocol [48]. The resulting kinetic enolate of **13** was treated with **10** at low temperature until the reaction was completed. Then, the crude β-hydroxycarbonylic was treated with *p*-toluenesulfonic acid (*p*-TsOH) at room temperature to obtain ketone **14** (Scheme 2). Compound **14** was a new compound, and its structure was established by NMR and HRMS data. Compound **14** was submitted to the Luche reduction (NaBH_4_/CeCl_3_) as for compound **11** to give pure racemic (±)-**3** (Scheme 2). This final compound was also a new compound and its structure was unequivocally determined by NMR and HRMS.

**Scheme 3:**
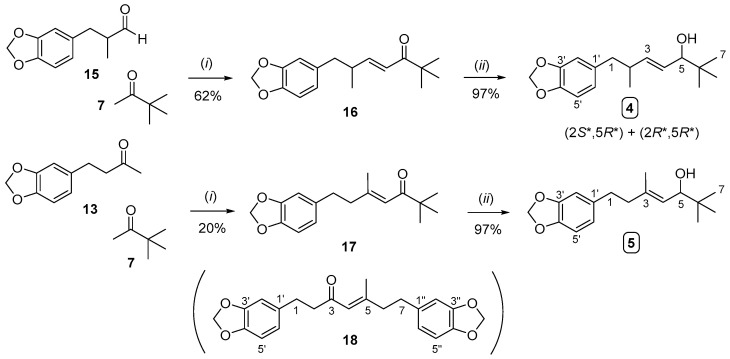
Synthesis of compounds **4** and **5**, two analogues to stiripentol (**1**). Reagents and conditions: (*i*) (a) LDA, THF, −80 °C; (b) *p*-TsOH, DCM, r.t.; (*ii*) NaBH_4_/CeCl_3_, DCM-MeOH, 0 °C.

In a similar manner, compounds **4** and **5** were synthesized using pinacolone-based lithium enolate chemistry (Scheme 3), following the same protocol to obtain **3**. Thus, pinacolone (**7**) was deprotonated with LDA at low temperature and then, the resulting lithium enolate being reacted with helional (**15**) to give the α,β-unsaturated ketone **16**, after treating the resulting β-hydroxycarbonylic crude with *p*-TsOH. This lithium-based protocol proved to be better than the classical cross-aldol reaction of **7** and **15** using KOH, since ketone **16** (Δ^3^ regioisomer) was obtained in the latter case together with its regioisomer Δ^2^. Finally, compound **16** was treated with the system NaBH_4_/CeCl_3_, as with ketones **11** and **14,** to give compound **4** as a 1:1 mixture of two diastereoisomers (Scheme 3). These compounds could not be separated by flash chromatography on silica gel but could be separated by semi-preparative HPLC using an acetonitrile–water mixture. Thus, pure racemic (±)-(2S*,5R*)-**4** and (±)-(2R*,5R*)-**4** were properly isolated. These two racemates have almost identical NMR data and could be separated into enantiomers, and their absolute configurations could be established (see Section 2.3 below). Both compounds **16** and **4** were new ones.

Regarding compound **5**, its synthesis was achieved by reaction of the lithium enolate of pinacolone (**7**) with piperonyl acetone (**13**) to give the α,β-unsaturated ketone **17** (after dehydration of the resulting β-hydroxycarbonylic crude with *p*-TsOH), which was regioselectively reduced into **5** by the Luche reduction (Scheme 3). Ketone **17** was formed from **7** and **13** with a much lower yield than the rest of ketones (**8**, **11**, **14**, **16**), which can be rationalized by the fact that **17** is the result of a cross-aldol addition between ketones and not between a ketone and an aldehyde (as for **8**, **11**, **14**, and **16**). The lower electrophilic character of ketones versus aldehydes and their comparatively higher steric hindrances explain this result. In fact, the aldol reaction between **7** and **13** was not completed, and unreacted starting ketone **13** was isolated in the column chromatography of the crude reaction. Furthermore, it is noteworthy that when **13** was treated with LDA and **7** was added afterwards (in order to obtain a regioisomer of **17**), the compound **17** was formed again and not the desired α,β-unsaturated ketone. This finding may be explained by the presence of the bulky *tert*-butyl group in pinacolone (**7**) which hinders the attack of the enolate of **13** on the carbonyl group of **7**, resulting in the exchange of lithium between both ketones. Another finding related to the great steric hindrance of pinacolone (**7**) is the isolation of some amounts of compound **18**, formed by the aldol condensation of **13** with itself (Scheme 3).

### 2.3. Resolution of Racemates ***1***, ***3***, and ***4*** into Their Enantiomers and Determination of Absolute Configuration

**Figure 2:**
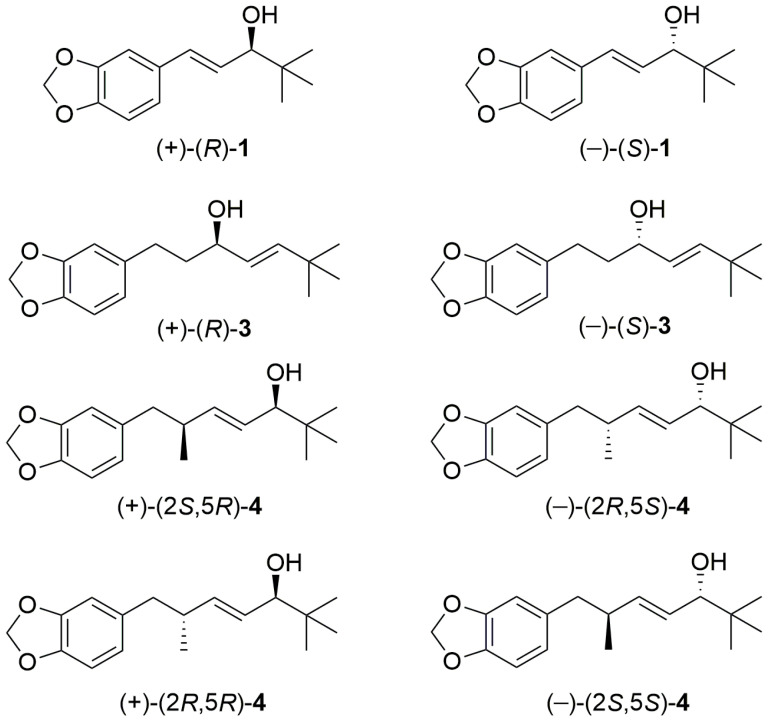
Enantiomers purified by chiral HPLC in this work (compound (±)-**2** was unstable in chiral HPLC and compound (±)-**5** showed low *h*LDHA inhibitory activity; see Section 2.4 below).

Stiripentol (STP, **1**) contains a stereogenic centre at the C-3 position and therefore exists as a pair of enantiomers (Figure 2). Both enantiomers were obtained by asymmetric synthesis [49,50], enzyme-catalyzed kinetic resolution of racemic STP [51], conventional synthesis, including a step involving a lipase-catalyzed resolution of a racemic precursor of STP [52], and by chiral HPLC of racemic STP [53]. For comparative purposes, we first tried to separate the enantiomers of STP by derivatizing (±)-**1** with (−)-(1*S*)-camphanic chloride to give a mixture of the camphanoate diastereoisomers suitable for being separated by conventional chromatography [54]. We have used this approach in the past to successfully separate stereoisomers of a sandalwood odorant [55]. However, the methodology did not work this time with (±)-**1,** since the resulting camphanoate diastereoisomers were partially hydrolyzed in contact with the stationary phase (silica gel) of the column chromatography and, in addition, rearranged to some extent to give mixtures that were difficult to analyze. For that reason, we decided to accomplish the resolution of (±)-**1** by HPLC using the chiral stationary phase Chiralpak^®^ IC (semi-preparative column) and a mixture of *n*-hexane and isopropanol as mobile phase. This chiral HPLC method allowed the separation of (±)-**1** to give (+)-(*R*)-**1** (purity: 98%) and (−)-(*S*)-**1** (purity: 99%) (Figure 2). The purity of each enantiomer was checked by HPLC using a chiral analytical column (Chiralpak^®^ IC) (chromatograms of the enantiomers are included in the Supplementary Materials; Figures S50 and S51). The assignment of the absolute configuration on C-3 for each enantiomer was made by polarimetry and comparison with the literature [49].

The same chiral HPLC protocol used for the resolution of (±)-**1** was followed to separate racemic iso-STP ((±)-**2**) into its enantiomers. However, it did not work in this case due to the tendency of (±)-**2** to rearrange into (±)-**1** on the chiral stationary phase during elution of components in the HPLC equipment as well as on a conventional HPLC column (Figure S43 in Supplementary Materials).

**Scheme 4:**
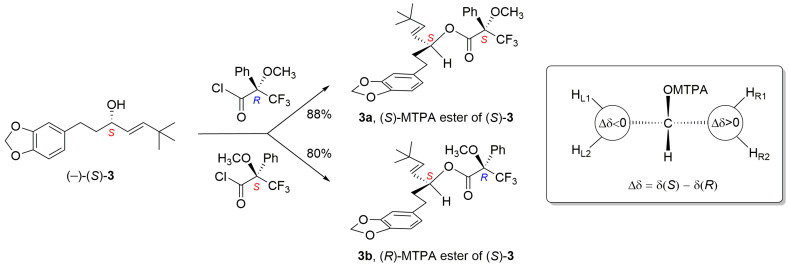
Esterification of (−)-(*S*)-**3** with (−)-(2*R*)-MTPA and (+)-(2*S*)-MTPA chlorides (modified Mosher’s method) to give MTPA esters **3a** and **3b** and assignment of absolute configuration on C-3.

On the other hand, the chiral HPLC protocol used to separate (±)-**1** did properly work with racemics (±)-**3**, (±)-(2S*,5R*)-**4** and (±)-(2R*,5R*)-**4**. Thus, (±)-**3** was separated into (+)-(*R*)-**3** (purity: 99%) and (−)-(*S*)-**3** (purity: 99%) in the Chiralpak^®^ IC semi-preparative column, using, in this case, a mixture of *n*-hexane and dichloromethane as mobile phase (Figure 2). To determine the absolute configuration of the enantiomers of **3**, the modified Mosher’s method [56] was used. Thus, the *levo* enantiomer (−)-**3** was treated with (−)-(2*R*)-MTPA chloride and (+)-(2*S*)-MTPA chloride, separately, affording the (*S*)-MTPA ester **3a** and (*R*)-MTPA ester **3b**, respectively (Scheme 4).

After assigning the ^1^H NMR signals of MTPA esters **3a** and **3b**, the sign of the difference in chemical shifts [Δδ = δ(*S*) − δ(*R*)] for pairs of protons next to the stereogenic carbon (C-3) was calculated (Table 1). The absolute configuration of the carbinol (C-3) can be reliably deduced by analyzing those signs and following the model reported in the literature [56]. In this case, the *levo* enantiomer (−)-**3** was assigned an *S* configuration because the positive differences in chemical shifts [Δδ = δ(*S*) − δ(*R*)] are located on the right side of the plane of the *O*-MTPA moiety, whereas those with negative values are all on the opposite side of that plane (Scheme 4).

**Figure 3:**
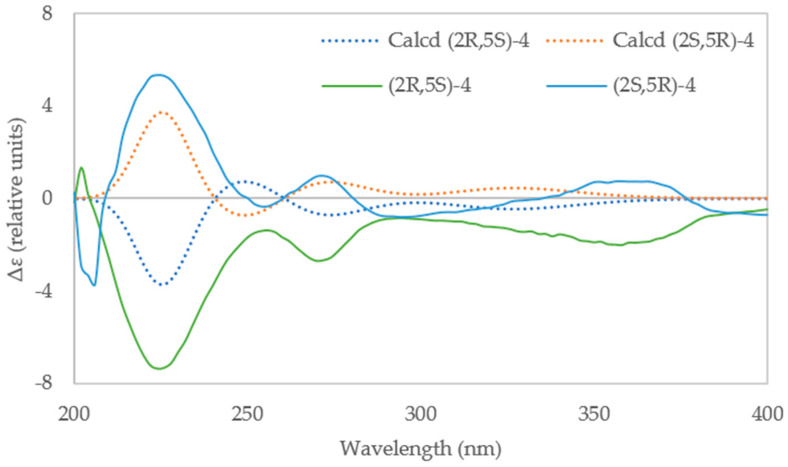
Comparative experimental and TD-DFT calculated ECD spectra for (+)-(2*R*,5*S*)-**4** and (−)-(2*S*,5*R*)-**4**.

In a similar manner, the two racemic diastereoisomers of compound **4** were separated into their four enantiomers by chiral HPLC using the same semi-preparative column as for (±)-**1** and (±)-**3**. Thus, the racemic (±)-(2S*,5R*)-**4** was separated into (+)-(2*S*,5*R*)-**4** (purity: 95%) and (−)-(2*R*,5*S*)-**4** (purity: 98%), whereas the racemic (±)-(2R*,5R*)-**4** was separated into (+)-(2*R*,5*R*)-**4** (purity: 99%) and (−)-(2*S*,5*S*)-**4** (purity: 99%) (Figure 2). The chromatogram of each enantiomer is included in Figures S55–S60 of the Supplementary Materials. These four enantiomers have two asymmetric carbons, one oxygenated (C-5) and the other bonded to a methyl group (C-2). The absolute configuration on their C-5 carbons (chiral secondary carbinols) could be determined by the modified Mosher’s method, as for the enantiomers of compound **3**. However, the absolute configuration on their C-2 carbons were not possible to establish by NMR and molecular modelling, since the coupling constants and chemical shifts in their NMR spectra were almost identical in all cases. Fortunately, the assignment of absolute stereochemistry could be achieved by electronic circular dichroism (ECD) spectroscopy [57], comparing experimental and calculated ECD spectra. Therefore, the *levo* enantiomer isolated from (±)-(2S*,5R*)-**4** displayed negative Cotton effects at 224 nm, 272 nm, and 358, which were the same as those for the *dextro* enantiomer but with opposite signs. Thus, the absolute configurations for these *levo* and *dextro* enantiomers were assigned as (2*R*,5*S*) and (2*S*,5*R*), respectively, by comparison of the predicted time-dependent density functional (TD-DFT) and experimental ECD data (Figure 3).

**Figure 4:**
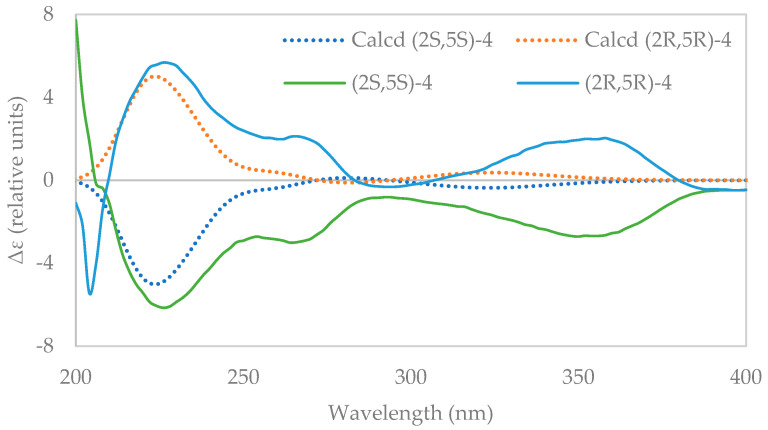
Comparative experimental and TD-DFT calculated ECD spectra for (−)-(2*S*,5*S*)-**4** and (+)-(2*R*,5*R*)-**4**.

Likewise, the *levo* enantiomer isolated from (±)-(2R*,5R*)-**4** showed negative Cotton effects at 226 nm, 264 nm (low intensity) and 358 nm, and same but opposite signs for the *dextro* enantiomer, which were consistent with those observed in calculated ECD spectra (Figure 4). Accordingly, the absolute configurations for these other *levo* and *dextro* enantiomers were assigned as (2*S*,5*S*) and (2*R*,5*R*), respectively (Figure 4).

Regarding racemic (±)-**5**, its resolution into enantiomers by chiral HPLC was not attempted because the inhibition percentage of the enzyme *h*LDHA was lower than 50% (see Section 2.4 below).

### 2.4. Inhibitory Activity of Racemates ***1***–***5*** and Selected Enantiomerically Pure Compounds Against hLDHA

The inhibitory effect over *h*LDHA catalytic activity (in the conversion of pyruvate to lactate) of racemic synthesized compounds **1**–**5** and some pure enantiomers separated from them was evaluated by a kinetic spectrofluorometric assay [33,37,38]. Briefly, the decrease in the co-substrate β-NADH fluorescence (λ_emission_ = 460 nm) was measured for 10 min, with different concentrations of the inhibitor, and the maximum slopes of these kinetic curves were compared with that obtained when no inhibitors were added (100% enzymatic activity).

In a first screening assay, all racemic compounds **1**–**5** were evaluated, at a single concentration of 500 μM, and the results were expressed as the percentage of inhibition at that concentration. The enantiomers of (±)-**1** were included in the study because of the interest in learning, for the first time, the inhibitory activity of the enantiomers of this commercial drug (Table 2). As observed (Table 2), racemic STP ((±)-**1**), its enantiomers ((+)-**1**, (−)-**1**), and racemic iso-STP ((±)-**2**) showed low percentages of inhibition (10–38%), whereas significant values (46–72% of inhibition) were achieved for compounds (±)-**3**, (±)-(2S*,5R*)-**4**, (±)-(2R*,5R*)-**4**, and (±)-**5**. Surprisingly, all designed compounds, analogues to STP and iso-STP, highlighted better inhibition percentages than the respective prototype precursors. Encouraged by the favourable results with the new compounds, we carried out, in a second phase of this study, a test of the inhibitory activity against *h*LDHA for the determination of IC_50_ values for those compounds that had shown a notable activity (**3**–**5**). In addition, the IC_50_ values for the pure enantiomers of **3** and **4** were determined (Figure 2, Table 2). The enantiomers of (±)-**2** were not successfully separated (see Section 2.3 above) and the separation of those of (±)-**5** was not attempted, because the percentage inhibition of (±)-**5** was below 50%. Therefore, in this second assay, eight different concentrations, in a range between 0 and 1000 µM, were prepared from the pure enantiomers of **3** and **4**. Thus, a dose response curve fitting the logarithm of inhibitor concentration vs. normalized enzymatic activity allowed us to determine IC_50_ values for each of them (Table 2). All these novel compounds showed IC_50_ values in the range of 100–200 µM, significantly improving the activity shown by **1** and **2** (with IC_50_ values higher than 500 µM). Nevertheless, it is worth noting that no remarkable differences in activity were observed between enantiomers of a given compound (Table 2). Among all of them, enantiomer (−)-(2*R*,5*S*)-**4** showed the lowest IC_50_ value (101.7 µM), which, comparing with the literature, is of the same order of magnitude as other LDHA inhibitors like galloflavin (IC_50_ = 103.6 µM) or luteolin-7-*O*-β-D-glucopiranoside (IC_50_ = 139.2 µM) [58].

The inhibitory activity of STP over *h*LDHA was measured for the first time by Sada et al. using purified mammalian lactate dehydrogenase (LDH) [24]. They found that STP, at a concentration of 500 μM, inhibited the lactate-to-pyruvate conversion as well as the pyruvate-to-lactate conversion by approximately 40%. We have found, for STP, an inhibition percentage of ca. 10% at the same concentration in the kinetic spectrofluorometric assay used here. This difference may be explained by the particular experimental conditions used in one assay compared to the other one. In any case, what is conclusive is that we have found several compounds related to STP that improve its activity, and that they will have to be compared with STP to determine whether they are more effective than STP, or not, in reducing urinary oxalate levels in vivo. In addition, the potential hepatotoxicity of these STP analogues should be evaluated, since it has been reported that some antiepileptic drugs, among which is STP, would have several side effects, such as liver injury [59]. However, on the other hand, STP proved to have a hepatoprotective effect on acetaminophen-induced hepatotoxicity in rats [60]. As a result of the existence of evidence in one direction or the other, the label for STP does not currently indicate that it causes liver injury. However, a recent study shows that adverse drug events of hepatobiliary disorders have been reported for STP in several pharmacovigilance databases [61] and, therefore, further work should be conducted on STP and analogues in the future.

## 3. Materials and Methods

### 3.1. General Experimental Methods

All solvents and reagents used in the synthesis or biological evaluations were purchased and used as received, except tetrahydrofuran (THF), which was previously dried, using standard methodologies [62]. Compound **9** was purchased from Alfa Aesar (Thermo Fisher Scientific, Waltham, MA, USA); compound **6**, diisopropylamine (DIPA), *n*-butyllithium (*n*-BuLi), (−)-(2*R*)-MTPA chloride, and (+)-(2*S*)-MTPA chloride were purchased from Sigma-Aldrich (Merck KGaA, Darmstadt, Germany); compound **7** and potassium hydroxide (KOH) were purchased from Fluka (Merck KGaA, Darmstadt, Germany); compounds **13** and **15** were purchased from Sensient Fragances (Sensient Technologies, Milwaukee, WI, USA); compound **10** and anhydrous cerium (III) chloride (CeCl_3_) were purchased from Thermo Fisher (Thermo Fisher Scientific, Waltham, MA, USA); sodium borohydride (NaBH_4_) was purchased from Riedel de Häen (Honeywell International Inc., Charlotte, NC, USA); *p*-toluenesulfonic acid (*p*-TsOH) was purchased from Panreac (Panreac Química, Barcelona, Spain). All solvents (*n*-hexane (Hex), diethyl ether (Et_2_O), dichloromethane (DCM), acetonitrile (ACN), ethyl acetate (EtOAc), and methanol (MeOH), THF) were purchased from VWR (Avantor, Radnor, PA, USA) except dimethylsulfoxide (DMSO), which was purchased from Panreac (Panreac Química, Barcelona, Spain).

Reactions were monitored by analytical thin-layer chromatography (TLC) on silica gel 60 F_254_ precoated aluminum sheets (0.25 mm, Merck Chemicals, Darmsdadt, Germany) and spots were detected using UV light (λ = 254 nm). Purification steps of synthesized α,β-unsaturated ketones (**8**, **11**, **14**, **16**, **17**) were carried out by column chromatography (CC) using Silica gel 60 (particle size 0.040-0.063 mm) (Merck Chemicals, Darmsdadt, Germany). Purification of synthesized compounds (**4** and **5**) was performed on a Shimadzu semipreparative HPLC (Shimadzu Corporation, Kyoto, Japan) that had a diode array detector (DAD), operating in the range of 190–800 nm (Shimadzu SPD-M20A, Shimadzu Corporation, Kyoto, Japan), at 25 °C, and using a C18 reversed-phase Spherisorb^®^ S5 ODS-2 column, 250 mm × 10 mm i.d., 5 μm (Waters Chromatography Division, Milford, MA, USA). Testing for the purity of synthesized compounds was performed on a Waters 1525 analytical HPLC (Waters Chromatography Division, Milford, MA, USA) that had a diode array detector (DAD), operating in the range of 190–800 nm (Waters 2998 Photodiode Array Detector, Waters Chromatography Division, Milford, MA, USA), at 25 °C and using a C18 reversed-phase Spherisorb^®^ S5 ODS-2 column, 250 mm × 3 mm i.d., 5 μm (Waters Chromatography Division, Milford, MA, USA). Chiral HPLC purifications were conducted on the same HPLC equipment previously described using a Chiralpak^®^ IC column, 250 mm × 10 mm i.d., 5 μm (Daicel Corporation, Illkrich, France). In the same way, the purity of separated enantiomers was determined on the HPLC equipment previously described using a Chiralpak^®^ IC column, 250 mm × 4.6 mm i.d., 5 μm (Daicel Corporation, Illkrich, France). The ECD measurements were achieved on a Chirascan V100 spectrophotometer (Applied Photophysics, Leatherhead, UK). Optical rotations were recorded at 589 nm (the Na D line) on (a) an Autopol^®^ V polarimeter (Rudolph Research Analytical, Karlsruhe, Baden-Württemberg, Germany) at room temperature and using quartz cells of 1 dm path length, and (b) on a Perkin Elmer 241 polarimeter (PerkinElmer Inc., Waltham, MA, USA), using quartz cells of 1 dm path length, in chloroform solutions. Melting points of solid compounds were recorded on an Electrothermal 9100 (Antylia Scientific, Vernon Hills, IL, USA) equipment.

^1^H NMR and ^13^C NMR spectra were measured on a Bruker Avance 400 spectrometer (Bruker Daltonik GmbH, Bremen, Germany) operating at 400 and 100 MHz for ^1^H and ^13^C, respectively. Deuterated chloroform (CDCl_3_) was used as solvent. Chemical shifts (in ppm) were referenced to solvent peaks, as an internal reference. The coupling constants (*J*) are expressed in hertz (Hz). The coupling system is described by using the following abbreviations: *s*, singlet; *d*, doublet; *t*, triplet; *m*, multiplet; *dd*, doublet of doublets; *ddd*, doublet of doublet of doublets; *dq*, doublet of quartets; *td*, triplet of doublets; *br s*, broad singlet; *br d*, broad doublet; *br q*, broad quartet. The total assignment of ^1^H and ^13^C signals was made using 2D NMR spectra such as COSY, HSQC, and HMBC.

Gas chromatography–Mass spectra (GC–MS) were conducted on a Thermo Trace GC 1610 gas chromatograph coupled to a Thermo Orbitrap Exploris GC 240 mass spectrometer (Thermo Fisher Scientific, Waltham, MA, USA). The GC was equipped with a TG-5SILMS capillary column (30 m × 0.25 mm × 0.25 μm), with the following specifications: carrier gas He; flow rate 1.2 mL/min; temperature programmed from 50 °C to 110 °C at 10 °C/min, then from 110 °C to 225 °C at 12 °C/min, then from 225 °C to 250 °C at 15 °C/min and remain at 250 °C for 17.25 min. For compounds **12** and **18,** the temperature was increased to 325 °C, with an injector temperature of 300 °C. Samples were injected using the split mode (ratio 1:100). High-resolution mass spectra (HRMS) were registered on the previously described equipment using an ionizing voltage of 70 eV (EI).

### 3.2. Synthesis of Stiripentol (***1***)

#### 3.2.1. Synthesis of Compound **8**

In a mixture of 3,3-dimethylbutan-2-one (**7**, 2.5 mL, 20.1 mmol) and 3′,4′-(methylenedioxy) benzaldehyde (**6**, 3.0 g, 20.0 mmol) in MeOH (65 mL), a solution of KOH (11.2 g, 200.3 mmol) in water (25 mL) was added in three times. The reaction vessel was heated at 75 °C, while under stirring, for 1 day (TLC monitored using Hex–Et_2_O 8:2). The resulting solution was cooled to room temperature and diluted with water (50 mL). The precipitated solid was filtered off, washed with water (10 mL) and MeOH (10 mL), and dried to give 3.0 g (65%) of compound **8** as a pale yellow solid.

(*E*)-1-(3′,4′-methylenedioxyphenyl)-4,4-dimethylpent-1-en-3-one (**8**): melting point: 91.6–93.0 °C; ^1^H NMR (400 MHz, CDCl_3_) δ (ppm) 7.59 (*d*, *J*_1,2_ = 15.5 Hz, 1H, H-1), 7.08 (*d*, *J*_2′,6′_ = 1.6 Hz, 1H, H-2′), 7.05 (*dd*, *J*_6′,5′_ = 8.0 Hz, *J*_6′,2′_ = 1.6 Hz, 1H, H-6′), 6.95 (*d*, *J*_2,1_ = 15.5 Hz, 1H, H-2), 6.81 (*d*, *J*_5′,6′_ = 8.0 Hz, 1H, H-5′), 6.00 (*s,* 2H, O-CH_2_-O), 1.21 (*s*, 9H, H-4a, H-4b, H-5); ^13^C NMR (100 MHz, CDCl_3_) δ (ppm) 204.4 (C-3), 149.7 (C-4′), 148.4 (C-3′), 142.8 (C-1), 129.5 (C-1′), 125.0 (C-6′), 118.9 (C-2), 108.7 (C-5′), 106.7 (C-2′), 101.7 (O-CH_2_-O), 43.3 (C-4), 26.5 (C-4a, C-4b, C-5). The spectroscopic data agree with those described in the literature [46]. HRMS (EI) (M^+^) calc. for C_14_H_16_O_3_ 232.10994, found 232.10953; *m*/*z* (%): 232.10953 (M^+^, 4), 175.03896 (M^+–^C_4_H_9_, 100), 145.02840 (79), 117.03348 (33), 89.03856 (39), 63.02292 (8).

#### 3.2.2. Synthesis of Stiripentol (**1**) from Compound **8**

NaBH_4_ (0.49 g, 13.0 mmol) was added portion-wise to an ice-cold solution of compound **8** (1.0 g, 4.3 mmol) in MeOH (10 mL) under stirring. The resulting solution was allowed to stir at room temperature for 18 h (TLC monitored using Hex–Et_2_O 8:2). Once the reaction had completed, the resulting solution was neutralized, adding, portion-wise, a mixture of ice–HCl 37%. The precipitated solid was filtered off, washed with cold water (10 mL) and dried to give 876 mg (87%) of stiripentol (**1**) as an off-white solid.

The purity of compound **1** was checked by analytical HPLC (see Supplementary Materials, Figure S42) using a C18 reversed-phase Spherisorb^®^ S5 ODS-2 column as described in Section 3.1. The analysis was performed under a linear gradient with ACN (solvent A) and water (solvent B) at a flow rate of 0.7 mL min^−1^, from 70% to 20% B in 20 min, with another 5 min to return to the initial conditions.

(*E*)-1-(3′,4′-methylenedioxyphenyl)-4,4-dimethylpent-1-en-3-ol (stiripentol (STP), **1**): melting point: 74.2–75.4 °C; ^1^H NMR (400 MHz, CDCl_3_) δ (ppm) 6.93 (*d*, *J*_2′,6′_ = 1.6 Hz, 1H, H-2′), 6.81 (*dd*, *J*_6′,5′_ = 8.0 Hz, *J*_6′,2′_ = 1.6 Hz, 1H, H-6′), 6.75 (*d*, *J*_5′,6′_ = 8.0 Hz, 1H, H-5′), 6.47 (*d*, *J*_1,2_ = 16.0 Hz, 1H, H-1), 6.11 (*dd*, *J*_2,1_ = 16.0 Hz, *J*_2,3_ = 7.0 Hz, 1H, H-2), 5.95 (*s,* 2H, O-CH_2_-O), 3.88 (*d*, *J*_3,2_ = 7.0 Hz, 1H, H-3), 0.96 (*s*, 9H, H-4a, H-4b, H-5); ^13^C NMR (100 MHz, CDCl_3_) δ (ppm) 148.1 (C-3′), 147.3 (C-4′), 131.6 (C-1), 131.5 (C-2′), 127.9 (C-2), 121.2 (C-6′), 108.4 (C-5′), 105.8 (C-2′), 101.2 (O-CH_2_-O), 81.1 (C-3), 35.4 (C-4), 25.9 (C-4a, C-4b, C-5). The spectroscopic data agree with those described in the literature [46]. HRMS (EI) (M^+^) calc. for C_14_H_18_O_3_ 234.12559, found 234.12527; *m/z* (%): 234.12527 (M^+^, 4), 177.05472 (M^+^–C_4_H_9_, 100), 159.04416 (M^+^–C_4_H_9_–H_2_O, 15), 147.04413 (89), 119.04920 (31), 91.05426 (29), 65.03860 (8). Analytical HPLC (λ = 254 nm): purity = 99%, *t*_R_ = 11.9 min.

### 3.3. Synthesis of Iso-Stiripentol (***2***)

#### 3.3.1. Synthesis of Compound **11**

Into a round flask, 3′,4′-(methylenedioxy)acetophenone (**9**, 150 mg, 0.90 mmol) was added, along with DMSO (1 mL) as solvent. Next, a third of the total needed amount of KOH (10 mg, 0.15 mmol) was added, portion-wise, and the mixture was kept under vigorous stirring at room temperature for 40 min. Then, the solution was cooled to 11–13 °C and trimethylacetaldehyde (**10**, 0.1 mL, 0.90 mmol) was added dropwise, followed by the rest of the KOH (20 mg, 0.30 mmol). The resulting solution was allowed to stir at 11–13 °C for 4 min (TLC monitored using Hex–Et_2_O 6:4). Once the reaction had completed, the mixture was neutralized with HCl 2N, and then water (5 mL) was added. The aqueous solution was extracted with DCM (3 × 10 mL). Then, the organic phases were joined and washed once with water (15 mL). The final organic phase was dried over anh. Na_2_SO_4_, and finally the solvent was removed in vacuo. The product obtained was purified by column chromatography (CC).

The purification step was performed by CC using mixtures of Hex–Et_2_O. With the mixture of solvents in a 9:1 ratio, pure compound **11** was obtained as a white solid (115 mg, 55%), whereas, with a 7:3 ratio, pure compound **12** was isolated as little white crystals (33.9 mg, 10%).

(*E*)-1-(3′,4′-methylenedioxyphenyl)-4,4-dimethylpent-2-en-1-one (**11**): melting point: 82.0–82.9 °C; ^1^H NMR (400 MHz, CDCl_3_) δ (ppm) 7.55 (*dd*, *J*_6′,5′_ = 8.1 Hz, *J*_6′,2′_ = 1.7 Hz, 1H, H-6′), 7.44 (*d*, *J*_2′,6′_ = 1.7 Hz, 1H, H-2′), 7.04 (*d*, *J*_3,2_ = 15.6 Hz, 1H, H-3), 6.86 (*d*, *J*_5′,6′_ = 8.1 Hz, 1H, H-5′), 6.73 (*d*, *J*_2,3_ = 15.6 Hz, 1H, H-2), 6.04 (*s*, 2H, O-CH_2_-O), 1.14 (*s*, 9H, H-4a, H-4b, H-5); ^13^C NMR (100 MHz, CDCl_3_) δ (ppm) 189.4 (C-1), 159.1 (C-3), 151.6 (C-4′), 148.3 (C-3′), 133.0 (C-1′), 124.8 (C-6′), 120.5 (C-2), 108.6 (C-2′), 107.9 (C-5′), 101.9 (O-CH_2_-O), 34.3 (C-4), 28.9 (C-4a, C-4b, C-5). HRMS (EI) (M^+^) calc. for C_14_H_16_O_3_ 232.10994, found 232.10942; *m/z* (%): 232.10942 (M^+^, 13), 217.08586 (36), 159.08040 (11), 149.02327 (100), 121.02836 (28) 65.03855 (15).

1,5-bis(3′,4′-methylenedioxyphenyl)-3-(*tert*-butyl)pentane-1,5-dione (**12**): melting point: 130.7-132.0 °C; ^1^H NMR (400 MHz, CDCl_3_) δ (ppm) 7.53 (*dd*, *J*_6′,5′_ = 8.2 Hz, *J*_6′,2′_ = 1.6 Hz, 2H, H-6′), 7.37 (*d*, *J*_2′,6′_ = 1.6 Hz, 2H, H-2′), 6.82 (*d*, *J*_5′,6′_ = 8.2 Hz, 2H, H-5′), 6.02 (*s,* 4H, O-CH_2_-O), 3.12 (*dd*, *J*_2b/4b,2a/4a_ = 15.7 Hz, *J*_2b/4b,3_ = 4.0 Hz, 2H, H-2b, H-4b), 2.77-2.83 (*m*, 1H, H-3), 2.70 (*dd*, *J*_2a/4a,2b/4b_ = 15.7 Hz, *J*_2a/4a,3_ = 8.0 Hz, 2H, H-2a, H-4a), 0.96 (*s*, 9H, H-4″a, H-4″b, H-4″c); ^13^C NMR (100 MHz, CDCl_3_) δ (ppm) 198.3 (C-1), 151.6 (C-4′), 148.2 (C-3′), 132.2 (C-1′), 124.3 (C-6′), 108.2 (C-2′), 107.9 (C-5′), 101.9 (O-CH_2_-O), 40.2 (C-3), 40.1 (C-2, C-4), 33.9 (C-4″), 27.7 (C-4″a, C-4″b, C-4″c). HRMS (EI) (M^+^) calc. for C_23_H_24_O_6_ 396.15729, found 396.15631; *m/z* (%): 396.15631 (M^+^, 0.1), 359.02866 (10), 299.06180 (36), 281.05133 (22), 225.04301 (100), 207.03247 (47), 151.02422 (16), 91.05425 (37).

#### 3.3.2. Synthesis of Iso-Stiripentol (**2**) from Compound **11**

Anh. CeCl_3_ (97 mg, 0.39 mmol) was added to a solution of compound **11** (91 mg, 0.39 mmol) in a DCM:MeOH mixture (1:1 *v*/*v*, 9.5 mL). The resulting solution was allowed to stir at room temperature for 30 min. Then, the mixture was cooled to 0 °C and NaBH_4_ (16 mg, 0.39 mmol) was added portion-wise for 10 min. Once the addition was completed, the mixture was kept stirring at 0 °C until the end of the reaction in a few minutes (TLC monitored using Hex–Et_2_O 5:5). Water (20 mL) was added to the resulting solution, and the mixture was extracted with Et_2_O (3 × 20 mL). The organic phases were joined, dried over anh. Na_2_SO_4_, and the solvent was removed in vacuo to yield 101 mg (98%) of compound **2** as a greyish syrup.

(*E*)-1-(3′,4′-methylenedioxyphenyl)-4,4-dimethylpent-2-en-1-ol (iso-stiripentol (iso-STP), **2**) ^1^H NMR (400 MHz, CDCl_3_) δ (ppm) 6.87 (*d*, *J*_2′,6′_ = 1.7 Hz, 1H, H-2′), 6.82 (*ddd*, *J*_6′,5′_ = 8.0 Hz, *J*_6′,2′_ = 1.7 Hz, *J*_6′,1_ = 0.7 Hz, 1H, H-6′), 6.78 (*d*, *J*_5′,6′_ = 8.0 Hz, 1H, H-5′), 5.95 (*s*, 2H, O-CH_2_-O), 5.77 (*dd*, *J*_3,2_ = 15.6 Hz, *J*_3,1_ = 1.2 Hz, 1H, H-3), 5.52 (*dd*, *J*_2,3_ = 15.6 Hz, *J*_2,1_ = 6.8 Hz, 1H, H-2), 5.07 (*brd*, *J*_1,2_ = 6.8 Hz, 1H, H-1), 1.02 (*s*, 9H, H-4a, H-4b, H-5); ^13^C NMR (100 MHz, CDCl_3_) δ (ppm) 147.8 (C-4′), 147.0 (C-3′), 143.5 (C-3), 137.8 (C-1′), 127.2 (C-2), 119.7 (C-6′), 108.2 (C-2′), 107.0 (C-5′), 101.1 (O-CH_2_-O), 75.3 (C-1), 33.0 (C-4), 29.6 (C-4a, C-4b, C-5). The spectroscopic data agree with those described in the literature [32]. HRMS (EI) (M^+^) calc. for C_14_H_18_O_3_ 234.12559, found 234.12529; *m*/*z* (%): 234.12529 (M^+^, 15), 217.08612 (8), 177.05473 (M^+^–C_4_H_9_, 80), 149.02341 (100), 147.04416 (89), 135.04414 (33), 119.04922 (37), 93.03353 (35), 77.03864 (16), 65.03861 (37). Analytical HPLC (λ = 254 nm): *t*_R_ = 10.9 min.

### 3.4. Synthesis of Compound ***3***

#### 3.4.1. Synthesis of Compound **14**

Into a dry round flask, DIPA (0.5 mL, 3.45 mmol) was added, with dry THF (5 mL) added as a solvent. The solution was cooled to 0 °C and *n*-BuLi (1.5 mL of 2,5 M hexane solution, 3.63 mmol) was added dropwise. The mixture was kept at 0 °C, under stirring, for 30 min. The resulting solution of lithium diisopropylamide (LDA), thus prepared, was cooled to –83 °C using an EtOAc–solid CO_2_ bath. Then, a solution of 4-(3′,4′-methylenedioxyphenyl)butan-2-one (**13**, 600 mg, 3.12 mmol) in dry THF (2 mL) was added dropwise. In the same way, ten minutes after the end of the addition of compound **13**, trimethylacetaldehyde (**10**, 0.3 mL, 2.69 mmol) was added. The reaction mixture was kept stirring, keeping the flask in the bath without adding more solid CO_2_ to it (TLC monitored using Hex–Et_2_O 4:6). Once the reaction had completed, water (3 mL) was added dropwise very slowly. Then, Et_2_O (30 mL) was added, and the organic layer was washed with brine (20 mL). Next, the aqueous phase was extracted with Et_2_O (2 × 30 mL) and the three remaining organic phases were joined and dried over anh. Na_2_SO_4_, and the solvent was removed in vacuo. The complex mixture obtained was immediately dissolved in DCM (20 mL) and *p*-TsOH (600 mg, 3.09 mmol) was added. The solution was kept at room temperature, undergoing stirring (TLC monitored using Hex–Et_2_O 4:6). Once the reaction had finished, a solution of NaHCO_3_ 10% p/v (30 mL) was added, and the mixture was extracted with DCM (2 × 30 mL). Finally, the organic phase was washed with brine (2 × 30 mL), dried over anh. Na_2_SO_4_, and the solvent was removed in vacuo. The product obtained was purified by column chromatography (CC).

The purification step was performed by CC using mixtures of Hex–Et_2_O. With the mixture of solvents in a 9:1 ratio, pure compound **14** was obtained as a yellowish oily mass (399 mg, 57%).

(*E*)-1-(3′,4′-methylenedioxyphenyl)-6,6-dimethylhept-4-en-3-one (**14**) ^1^H NMR (400 MHz, CDCl_3_) δ (ppm) 6.79 (*d*, *J*_5,4_ = 16.2 Hz, 1H, H-5), 6.72 (*d*, *J*_5′,6′_ = 7.9 Hz, 1H, H-5′), 6.69 (*d*, *J*_2′,6′_ = 1.6 Hz, 1H, H-2′), 6.65 (*dd*, *J*_6′,5′_ = 7.9 Hz, *J*_6′,2′_ = 1.6 Hz, 1H, H-6′), 5.99 (*d*, *J*_4,5_ = 16.2 Hz, 1H, H-4), 5.91 (*s*, 2H, O-CH_2_-O), 2.82–2.87 (*m*, 4H, H-1, H-2), 1.06 (*s*, 9H, H-6a, H-6b, H-7); ^13^C NMR (100 MHz, CDCl_3_) δ (ppm) 200.3 (C-3), 157.5 (C-5), 147.7 (C-3′), 145.9 (C-4′), 135.3 (C-1′), 125.5 (C-4), 121.3 (C-6′), 109.0 (C-2′), 108.4 (C-5′), 100.9 (O-CH_2_-O), 42.2 (C-2), 33.9 (C-6), 30.0 (C-1), 28.8 (C-6a, C-6b, C-7). HRMS (EI) (M^+^) calc. for C_16_H_20_O_3_ 260.14124, found 260.14096; *m/z* (%): 260.14096 (M^+^, 4), 203.07036 (M^+^–C_4_H_9_, 100), 173.05981 (16), 145.06487 (16), 135.04410 (93), 119.04919 (20), 91.05425 (28), 77.03861 (18), 65.03860 (8).

#### 3.4.2. Synthesis of Compound **3** from Compound **14**

Anh. CeCl_3_ (254 mg, 1.03 mmol) was added to a solution of compound **14** (268 mg, 1.03 mmol) in a DCM:MeOH mixture (1:1 *v*/*v*, 10 mL). The resulting solution was allowed to stir at room temperature for 30 min. Then, the mixture was cooled to 0 °C and NaBH_4_ (41 mg, 1.03 mmol) was added portion-wise for 10 min. Once the addition was completed, the mixture remained stirring at 0 °C until the end of the reaction after a few minutes (TLC monitored using Hex–Et_2_O 4:6). Water (30 mL) was added to the resulting solution, and the mixture was extracted with Et_2_O (3 × 30 mL). The organic phases were joined and washed with water (30 mL). The final organic layer was dried over anh. Na_2_SO_4_, and the solvent was removed in vacuo to yield 254 mg (94%) of compound **3** as a yellowish syrup.

The purity of compound **3** was checked by analytical HPLC (see Supplementary Materials, Figure S44) using a C18 reversed-phase Spherisorb^®^ S5 ODS-2 column, as described in Section 3.1. The analysis was performed under a linear gradient with ACN (solvent A) and water (solvent B) at a flow rate of 0.7 mL min^−1^, from 70% to 20% B in 20 min, with another 5 min to return to the initial conditions.

(*E*)-1-(3′,4′-methylenedioxyphenyl)-6,6-dimethylhept-4-en-3-ol (**3**) ^1^H NMR (400 MHz, CDCl_3_) δ (ppm) 6.72 (d, J_5′,6′_ = 7.8 Hz, 1H, H-5′), 6.69 (d, J_2′,6′_ = 1.6 Hz, 1H, H-2′), 6.64 (dd, J_6′,5′_ = 7.8 Hz, J_6′,2′_ = 1.6 Hz, 1H, H-6′), 5.91 (s, 2H, O-CH_2_-O), 5.66 (d, J_5,4_ = 15.7 Hz, 1H, H-5), 5.38 (dd, J_4,5_ = 15.7 Hz, J_4,3_ = 7.1 Hz, 1H, H-4), 4.02–4.07 (m, 1H, H-3), 2.54–2.66 (m, 2H, H-1), 1.70–1.88 (m, 2H, H-2), 1.01 (s, 9H, H-6a, H-6b, H-7); ^13^C NMR (100 MHz, CDCl_3_) δ (ppm) 147.7 (C-3′), 145.7 (C-4′), 143.5 (C-5), 136.0 (C-1′), 127.6 (C-4), 121.3 (C-6′), 109.1 (C-2′), 108.3 (C-5′), 100.9 (O-CH_2_-O), 72.6 (C-3), 39.3 (C-2), 32.9 (C-6), 31.7 (C-1), 29.6 (C-6a, C-6b, C-7). HRMS (EI) (M^+^) calc. for C_16_H_22_O_3_ 262.15689, found 262.15640; *m*/*z* (%): 262.15640 (M^+^, 4), 205.08592 (M^+^–C_4_H_9_, 7), 187.07536 (M^+^–C_4_H_9_–H_2_O, 7), 136.05182 (40), 135.04401 (100), 91.05419 (15), 77.03856 (13). Analytical HPLC (λ = 285 nm): purity = >99%, t_R_ = 13.8 min.

### 3.5. Synthesis of Compound ***4***

#### 3.5.1. Synthesis of Compound **16**

Into a dry round flask, DIPA (0.3 mL, 2.31 mmol) was added, with dry THF (4 mL) used as a solvent. The solution was cooled to 0 °C and *n*-BuLi (0.9 mL of 2.5 M hexane solution, 2.43 mmol) was added dropwise. The mixture was kept at 0 °C under stirring for 30 min. The resulting solution of lithium diisopropylamide (LDA) thus prepared, was cooled to −83 °C using an EtOAc:solid CO_2_ bath. Then, 3,3-dimethylbutan-2-one (**7**, 0.3 mL, 2.09 mmol) was added dropwise. In the same way, ten minutes after the end of the addition of compound **7** a solution of 2-methyl-3-(3′,4′-methylenedioxyphenyl)propanal (**15**, 346 mg, 1.80 mmol) in dry THF (2 mL) was added. The reaction mixture was kept under stirring and keeping the flask in the bath without adding more solid CO_2_ to it (TLC monitored using Hex–Et_2_O 6:4). Once the reaction was completed, water (1.5 mL) was added dropwise very slowly. Then, Et_2_O (20 mL) was added and the organic layer was washed with brine (10 mL). Next, the aqueous phase was extracted with Et_2_O (2 × 20 mL) and the three remaining organic phases were joined, dried over anh. Na_2_SO_4_, and the solvent was removed in vacuo. The complex mixture obtained was immediately dissolved in DCM (20 mL) and *p*-TsOH (402 mg, 2.07 mmol) was added. The solution was kept at room temperature, with stirring being maintained (TLC monitored using Hex–Et_2_O 5:5). Once the reaction had finished, a solution of NaHCO_3_ 10% p/v (20 mL) was added and the mixture was extracted twice with DCM (2 × 20 mL). Finally, the organic phase was washed with brine (2 × 20 mL), dried over anh. Na_2_SO_4_, and the solvent was removed in vacuo. The product obtained was purified by column chromatography (CC).

The purification step was performed by CC using mixtures of Hex–Et_2_O. With the mixture of solvents in a 9:1 ratio, pure compound **16** was obtained as a yellowish oily mass (307 mg, 62%).

(*E*)-1-(3′,4′-methylenedioxyphenyl)-2,6,6-trimethylhept-3-en-5-one (**16**) ^1^H NMR (400 MHz, CDCl_3_) δ (ppm) 6.83 (*dd*, *J*_3,4_ = 15.3 Hz, *J*_3,2_ = 7.3 Hz, 1H, H-3), 6.70 (*d*, *J*_5′,6′_ = 7.8 Hz, 1H, H-5′), 6.60 (*d*, *J*_2′,6′_ = 1.6 Hz, 1H, H-2′), 6.55 (*dd*, *J*_6′,2′_ = 1.6 Hz, *J*_6′,5′_ = 7.8 Hz, 1H, H-6′), 6.33 (*dd*, *J*_4,3_ = 15.3 Hz, *J*_4,2_ = 0.9 Hz, 1H, H-4), 5.90 (*d*, *J*_CH2a,CH2b_ = 2.8 Hz, 1H, O-CH_2a_-O), 5.90 (*d*, *J*_CH2b,CH2a_ = 2.8 Hz, 1H, O-CH_2b_-O), 2.49-2.66 (*m*, 2H, H-1), 2.49–2.56 (*m*, 1H, H-2), 1.09 (*s*, 9H, H-6a, H-6b, H-7), 1.04 (*d*, *J*_2a,2_ = 6.2 Hz, 3H, H-2a); ^13^C NMR (100 MHz, CDCl_3_) δ (ppm) 204.5 (C-5), 151.4 (C-3), 147.5 (C-3′), 145.9 (C-4′), 133.7 (C-1′), 123.0 (C-4), 122.2 (C-6′), 109.6 (C-2′), 108.1 (C-5′), 100.9 (O-CH_2_-O), 43.0 (C-6), 42.4 (C-1), 39.0 (C-2), 26.2 (C-6a, C-6b, C-7), 19.1 (C-2a). HRMS (EI) (M^+^) calc. for C_17_H_22_O_3_ 274.15689, found 274.15665; *m/z* (%): 274.15665 (M^+^, 2), 217.08615 (M^+^–C_4_H_9_, 2), 174.06767 (3), 135.04407 (100), 115.05430 (2), 77.03863 (8).

#### 3.5.2. Synthesis of Compound **4** from Compound **16**

Anh. CeCl_3_ (163 mg, 0.66 mmol) was added to a solution of compound **16** (181 mg, 0.66 mmol) in a DCM–MeOH mixture (1:1 *v*/*v*, 10 mL). The resulting solution was allowed to stir at room temperature for 30 min. Then, the mixture was cooled to 0 °C and NaBH_4_ (26 mg, 0.66 mmol) was added portion-wise for 10 min. Once the addition was completed, stirring was maintained at 0 °C until the end of the reaction a few minutes later (TLC monitored using Hex–Et_2_O 7:3). Water (30 mL) was added to the resulting solution, and the mixture was extracted with Et_2_O (3 × 30 mL). The organic phases were joined and washed with water (30 mL). The final organic layer was dried over anh. Na_2_SO_4_, and the solvent was removed in vacuo to yield 182 mg (97%) of compound **4**. Further purification was carried out by HPLC.

The purification step was performed by semipreparative HPLC using a C18 reversed-phase Spherisorb^®^ S5 ODS-2 column as described in Section 3.1, with ACN (solvent A) and water (solvent B) as the mobile phase at a flow rate of 5 mL min^−1^. The best separation was achieved with isocratic conditions of 55% B, and the total run time was 50 min. Both diastereomers (2S*,5R*)-**4** and (2R*,5R*)-**4** were isolated.

The purity of compounds (2S*,5R*)-**4** and (2R*,5R*)-**4** were checked by analytical HPLC (see Supplementary Materials, Figures S46 and S47) using a C18 reversed-phase Spherisorb^®^ S5 ODS-2 column, as described in Section 3.1. The analysis was performed with ACN (solvent A) and water (solvent B) at a flow rate of 0.7 mL min^−1^ and under isocratic conditions of 55% B and the total run time was 30 min.

(2S*,5R*)-(*E*)-1-(3′,4′-methylenedioxyphenyl)-2,6,6-trimethylhept-3-en-5-ol ((2S*,5R*)-**4**) ^1^H NMR (400 MHz, CDCl_3_) δ (ppm) 6.70 (*d*, *J*_5′,6′_ = 7.9 Hz, 1H, H-5′), 6.62 (*d*, *J*_2′,6′_ = 1.6 Hz, 1H, H-2′), 6.57 (*dd*, *J*_6′,5′_ = 7.9 Hz, *J*_6′,2′_ = 1.6 Hz, 1H, H-6′), 5.90 (*d*, *J*_CH2a,CH2b_ = 2.5 Hz, 1H, O-CH_2a_-O), 5.90 (*d*, *J*_CH2b,CH2_ = 2.5 Hz, 1H, O-CH_2b_-O), 5.52 (*ddd*, *J*_3,4_ = 15.4 Hz, *J*_3,2_ = 7.5 Hz, *J*_3,5_ = 0.3 Hz, 1H, H-3), 5.40 (*ddd*, *J*_4,3_ = 15.4 Hz, *J*_4,5_ = 7.5 Hz, *J*_4,2_ = 0.7 Hz, 1H, H-4), 3.64 (*d*, *J*_5,4_ = 7.5 Hz, 1H, H-5), 2.46–2.57 (*m*, 2H, H-1), 2.35–2.46 (*m*, 1H, H-2), 1.00 (*d*, *J*_2a,2_ = 6.6 Hz, 3H, H-2a), 0.81 (*s*, 9H, H-6a, H-6b, H-7); ^13^C NMR (100 MHz, CDCl_3_) δ (ppm) 147.5 (C-3′), 145.7 (C-4′), 138.6 (C-3), 134.6 (C-1′), 128.7 (C-4), 122.1 (C-6′), 109.7 (C-2′), 108.1 (C-5′), 100.8 (O-CH_2_-O), 81.2 (C-5), 43.3 (C-1), 38.8 (C-2), 34.8 (C-6), 25.8 (C-6a, C-6b, C-7), 20.1 (C-2a). HRMS (EI) (M^+^) calc. for C_17_H_24_O_3_ 276.17254, found 276.17224; *m/z* (%): 276.17224 (M^+^, 0.9), 258.16165 (1), 219.10179 (M^+^–C_4_H_9_, 1), 201.09117 (M^+^–C_4_H_9_–H_2_O, 3), 175.07552 (1), 135.04404 (100), 105.03354 (1), 77.03862 (7). Analytical HPLC (λ = 285 nm): purity = 98%, *t*_R_ = 20.7 min.

(2R*,5R*)-(*E*)-1-(3′,4′-methylenedioxyphenyl)-2,6,6-trimethylhept-3-en-5-ol ((2R*,5R*)-**4**) ^1^H NMR (400 MHz, CDCl_3_) δ (ppm) 6.71 (*d*, *J*_5′,6′_ = 7.9 Hz, 1H, H-5′), 6.63 (*d*, *J*_2′,6′_ = 1.6 Hz, 1H, H-2′), 6.57 (*dd*, *J*_6′,5′_ = 7.9 Hz, *J*_6′,2′_ = 1.6 Hz, 1H, H-6′), 5.91 (*s*, 2H, O-CH_2_-O), 5.56 (*ddd*, *J*_3,4_ = 15.5 Hz, *J*_3,2_ = 7.1 Hz, *J*_3,5_ = 0.5 Hz, 1H, H-3), 5.42 (*ddd*, *J*_4,3_ = 15.5 Hz, *J*_4,5_ = 7.3 Hz, *J*_4,2_ = 0.7 Hz, 1H, H-4), 3.66 (*d*, *J*_5,4_ = 7.3 Hz, 1H, H-5), 2.47–2.59 (*m*, 2H, H-1), 2.35–2.47 (*m*, 1H, H-2), 0.99 (*d*, *J*_2a,2_ = 6.6 Hz, 3H, H-2a), 0.85 (*s*, 9H, H-6a, H-6b, H-7); ^13^C NMR (100 MHz, CDCl_3_) δ (ppm) 147.5 (C-3′), 145.7 (C-4′), 138.4 (C-3), 134.6 (C-1′), 128.6 (C-4), 122.1 (C-6′), 109.6 (C-2′), 108.0 (C-5′), 100.8 (O-CH_2_-O), 81.1 (C-5), 43.3 (C-1), 38.6 (C-2), 34.9 (C-6), 25.8 (C-6a, C-6b, C-7), 20.0 (C-2a). HRMS (EI) (M^+^) calc. for C_17_H_24_O_3_ 276.17254, found 276.17221; *m/z* (%): 276.17221 (M^+^, 1), 258.16165 (1), 219.10173 (M^+^–C_4_H_9_, 1), 201.09114 (M^+^–C_4_H_9_–H_2_O, 2), 135.04402 (100), 105.03353 (1), 77.03860 (7). Analytical HPLC (λ = 285 nm): purity = 99%, *t*_R_ = 23.5 min.

### 3.6. Synthesis of Compound ***5***

#### 3.6.1. Synthesis of Compound **17**

Into a dry round flask, DIPA (0.6 mL, 4.34 mmol) was added, and dry THF (6 mL) was used as a solvent. The solution was cooled to 0 °C and *n*-BuLi (1.8 mL of 2,5 M hexane solution, 4.57 mmol) was added dropwise. The mixture was kept at 0 °C, maintaining stirring for 30 min. The resulting solution of lithium diisopropylamide (LDA), thus prepared, was cooled to –83 °C using an EtOAc–solid CO_2_ bath. Then, 3,3-dimethylbutan-2-one (**7**, 0.5 mL, 3.93 mmol) was added dropwise. In the same way, ten minutes after the end of the addition of compound **7,** a solution of 4-(3′,4′-methylenedioxyphenyl)butan-2-one (**13**, 651 mg, 3.39 mmol) in dry THF (1 mL) was added. Stirring continued for the reaction mixture, and the flask was kept in the bath without adding more solid CO_2_ to it (TLC monitored using Hex–Et_2_O 4:6). Once the reaction had completed, water (3 mL) was added dropwise very slowly. Then, Et_2_O (30 mL) was added, and the organic layer was washed with brine (20 mL). Next, the aqueous phase was extracted with Et_2_O (2 × 30 mL) and the three remaining organic phases were joined, dried over anh. Na_2_SO_4_, and the solvent was removed in vacuo. The complex mixture obtained was immediately dissolved in DCM (20 mL) and *p*-TsOH (755 mg, 3.90 mmol) was added. The solution was kept at room temperature while stirring was maintained (TLC monitored using Hex–Et_2_O 4:6). Once the reaction was finished, a solution of NaHCO_3_ 10% p/v (30 mL) was added and the mixture was extracted twice with DCM (2 × 30 mL). Finally, the organic phase was washed with brine (2 × 30 mL), dried over anh. Na_2_SO_4_, and the solvent was removed in vacuo. The product obtained was purified by column chromatography (CC).

The purification step was performed by CC using mixtures of Hex–Et_2_O. With the mixture of solvents in a 93:7 ratio, pure compound **17** was obtained as a yellowish oily mass (187 mg, 20%), whereas, with a 91:9 ratio, 383 mg of reactant **13** was isolated.

(*E*)-1-(3′,4′-methylenedioxyphenyl)-3,6,6-trimethylhept-3-en-5-one (**17**) ^1^H NMR (400 MHz, CDCl_3_) δ (ppm) 6.70 (*d*, *J*_5′,6′_ = 7.8 Hz, 1H, H-5′), 6.63 (*d*, *J*_2′,6′_ = 1.8 Hz, 1H, H-2′), 6.58 (*dd*, *J*_6′,5′_ = 7.8 Hz, *J*_6′,2′_ = 1.8 Hz, 1H, H-6′), 6.17–6.18 (*m*, 1H, H-4), 5.91 (*s*, 2H, O-CH_2_-O), 2.68–2.72 (*m*, 2H, H-1), 2.38 (*t*, *J*_2,1_ = 7.8 Hz, 2H, H-2), 2.10 (*d*, *J*_3a,4_ = 1.3 Hz, 3H, H-3a), 1.08 (*s*, 9H, H-6a, H-6b, H-7); ^13^C NMR (100 MHz, CDCl_3_) δ (ppm) 206.5 (C-5), 157.0 (C-3), 147.7 (C-3′), 145.9 (C-4′), 135.1 (C-1′), 121.3 (C-6′), 120.1 (C-4), 108.9 (C-2′), 108.3 (C-5′), 100.9 (O-CH_2_-O), 43.8 (C-6), 43.6 (C-2), 33.9 (C-1), 26.6 (C-6a, C-6b, C-7), 19.5 (C-3a). HRMS (EI) (M^+^) calc. for C_17_H_22_O_3_ 274.15689, found 274.15659; *m*/*z* (%): 274.15659 (M^+^, 0.6), 259.13306 (4), 217.08601 (M^+^–C_4_H_9_, 8), 189.09114 (2), 135.04402 (100), 115.05428 (2), 77.03860 (8).

Opposite reaction: a solution of 4-(3′,4′-methylenedioxyphenyl)butan-2-one (**13**, 600 mg, 3.12 mmol), in dry THF (1 mL), was added to the previously prepared LDA solution, and then 3,3-dimethylbutan-2-one (**7**, 0.3 mL, 2.69 mmol) was used as the electrophile. The second step was performed using a solution of *p*-TsOH (600 mg, 3.09 mmol) in DCM (20 mL). Once the work-up was completed, the obtained product was purified by column chromatography (CC) using mixtures of Hex–Et_2_O. With the solvent mixture in a 93:7 ratio, pure compound **17** was isolated as a yellowish oily mass (69 mg, 9%), whereas with a 91:9 ratio, pure compound **18** was obtained as a pale yellow solid (31 mg, 3%).

(*E*)-1,7-bis(3′,4′-methylenedioxyphenyl)-5-methylhept-4-en-3-one (**18**): melting point: 76.1–77.2 °C. ^1^H NMR (400 MHz, CDCl_3_) δ (ppm) 6.78 (*d*, *J*_2′,6′_ = 0.8 Hz, 1H, H-2′), 6.73 (*d*, *J*_5′,6′_ = 7.9 Hz, 1H, H-5′), 6.72 (*d*, *J*_5″,6″_ = 7.9 Hz, 1H, H-5″), 6.72 (*dd*, *J*_6′,5′_ = 7.9 Hz, *J*_6′,2′_ = 0.8 Hz, 1H, H-6′), 6.68 (*d*, *J*_2″,6″_ = 1.1 Hz, 1H, H-2″), 6.64 (*dd*, *J*_6″,5″_ = 7.9 Hz, *J*_6″,2″_ = 1.1 Hz, 1H, H-6″), 6.06 (*brs*, 1H, H-4), 5.91 (*s*, 2H, O-CH_2_-O), 2.65-2.85 (*m*, 8H, H-1, H-2, H-6, H-7), 1.85 (*d*, *J*_5a,6_ = 1.1 Hz, 3H, H-5a); ^13^C NMR (100 MHz, CDCl_3_) δ (ppm) 199.5 (C-3), 158.7 (C-5), 147.7 (C-3′), 147.6 (C-3″), 145.8 (C-4′, C-4″), 135.7 (C-1′), 135.3 (C-1″), 124.1 (C-4), 121.3 (C-6′), 121.2 (C-6″), 109.2 (C-2′), 109.0 (C-2″), 108.3 (C-5′), 108.2 (C-5″), 100.9 (O-CH_2_-O), 46.2 (C-2), 36.4 (C-6), 34.3 (C-1), 30.0 (C-7), 26.0 (C-5a). HRMS (EI) (M^+^) calc. for C_22_H_22_O_5_ 366.14672, found 366.14615; *m*/*z* (%): 366.14615 (M^+^, 1), 299.06174 (33), 281.05118 (38), 225.04294 (100), 207.03238 (77), 135.04407 (99), 91.05422 (21), 77.03859 (18).

#### 3.6.2. Synthesis of Compound **5** from Compound **17**

Anh. CeCl_3_ (80 mg, 0.32 mmol) was added to a solution of compound **17** (87 mg, 0.32 mmol) in a DCM:MeOH mixture (1:1 *v*/*v*, 8 mL). The resulting solution was allowed to stir at room temperature for 30 min. Then, the mixture was cooled to 0 °C and NaBH_4_ (26 mg, 0.64 mmol) was added, portion-wise. Once the addition was completed, stirring was maintained at 0 °C until the end of the reaction (TLC monitored using Hex–Et_2_O 6:4). Water (20 mL) was added to the resulting solution, and the mixture was extracted with Et_2_O (3 × 30 mL). The organic phases were joined and washed with water (30 mL). The final organic layer was dried over anh. Na_2_SO_4_, and the solvent was removed in vacuo to yield 86 mg (97%) of compound **5**. Further purification was carried out by HPLC.

The purification step was performed by semipreparative HPLC using a C18 reversed-phase Spherisorb^®^ S5 ODS-2 column as described in Section 3.1, with ACN (solvent A) and water (solvent B) as mobile phase at a flow rate of 5 mL min^−1^. Purification was achieved with isocratic conditions of 55% B, and the total run time was 45 min.

The purity of compound **5** was checked by analytical HPLC (see Supplementary Materials, Figure S48) using a C18 reversed-phase Spherisorb^®^ S5 ODS-2 column as described in Section 3.1. The analysis was performed with ACN (solvent A) and water (solvent B) at a flow rate of 0.7 mL min^−1^ and under isocratic conditions of 55% B, and the total run time was 35 min.

(*E*)-1-(3′,4′-methylenedioxyphenyl)-3,6,6-trimethylhept-3-en-5-ol (**5**) ^1^H NMR (400 MHz, CDCl_3_) δ (ppm) 6.71 (*d*, *J*_5′,6′_ = 7.9 Hz, 1H, H-5′), 6.66 (*d*, *J*_2′,6′_ = 1.6 Hz, 1H, H-2′), 6.61 (*dd*, *J*_6′,5′_ = 7.9 Hz, *J*_6′,2′_ = 1.6 Hz, 1H, H-6′), 5.90 (*s*, 2H, O-CH_2_-O), 5.19 (*dq*, *J*_4,5_ = 9.3 Hz, *J*_4,3a_ = 1.3 Hz, 1H, H-4), 3.98 (*d*, *J*_5,4_ = 9.3 Hz, 1H, H-5), 2.60-2.73 (*m*, 2H, H-1), 2.29 (*t*, *J*_2,1_ = 7.8 Hz, 2H, H-2), 1.71 (*d*, *J*_3a,4_ = 1.3 Hz, 3H, H-3a), 0.84 (*s*, 9H, H-6a, H-6b, H-7); ^13^C NMR (100 MHz, CDCl_3_) δ (ppm) 147.6 (C-3′), 145.7 (C-4′), 138.6 (C-3), 135.8 (C-1′), 125.7 (C-4), 121.2 (C-6′), 109.0 (C-2′), 108.2 (C-5′), 100.9 (O-CH_2_-O), 76.0 (C-5), 42.1 (C-2), 35.5 (C-6), 34.2 (C-1), 25.6 (C-6a, C-6b, C-7), 17.0 (C-3a). HRMS (EI) (M^+^) calc. for C_17_H_24_O_3_ 276.17254, found 276.17212; *m/z* (%): 276.17212 (M^+^, 0.8), 219.10165 (M^+^–C_4_H_9_, 3), 201.09108 (M^+^–C_4_H_9_–H_2_O, 3), 175.07542 (2), 135.04399 (100), 91.05421 (3), 77.03858 (8). Analytical HPLC (λ = 285 nm): purity = >99%, *t*_R_ = 18.6 min.

### 3.7. Resolution of Racemates and Optical Rotatory Power Analysis

Stiripentol (**1**) and the most active racemic compounds synthesized (**3**, (2S*,5R*)-**4** and (2R*,5R*)-**4**) were purified by chiral HPLC, using a Chiralpak^®^ IC column as described in Section 3.1. The proper purity of each enantiomer was also checked by analytical HPLC (chromatograms of each pure enantiomer are included in the Supplementary Materials, Figures S49–S60) using a Chiralpak^®^ IC column (see Section 3.1) and the same conditions as the purifications, except a flow rate of 0.7 mL min^−1^ for stiripentol (**1**) and 1 mL min^−1^ was used for compounds **3**, (2S*,5R*)-**4** and (2R*,5R*)-**4**. The optical rotatory power analysis was evaluated for each pure enantiomer using the polarimeter described in Section 3.1.

Stiripentol (**1**) was purified using Hex (solvent A) and isopropanol (solvent B) as the mobile phase at a flow rate of 4 mL min^−1^. Solutions of stiripentol were prepared in mixtures of Hex–isopropanol (1:1, *v*/*v*) at concentrations of 100 mg mL^−1^, and an injection volume of 70 μL. The best separation was achieved with isocratic conditions of 2% B and a total run time of 30 min. The levorotatory enantiomer, (−)-(*S*)-**1** ([α]_D_ = −27.2°, c = 0.97 cg/mL, CHCl_3_), was isolated with a retention time of 21.6 min (λ = 254 nm, purity: >99%) while the dextrorotatory enantiomer, (+)-(*R*)-**1** ([α]_D_ = +27.8°, c = 0.94 cg/mL, CHCl_3_), was isolated with a retention time of 25.6 min (λ = 254 nm, purity: 98%).

Compound **3** was purified using Hex (solvent A) and DCM (solvent B) as mobile phase at a flow rate of 5 mL min^−1^. Solutions of **3** were prepared in mixtures of Hex:DCM (7:3, *v*/*v*) at concentrations of 100 mg mL^−1^, and an injection volume of 40 μL. The best separation was achieved with isocratic conditions of 30% B and a total run time of 30 min. The levorotatory enantiomer, (−)-(*S*)-**3** (${\left[\alpha \right]}_{D}^{20}$ = −6.9°, c = 1.07 cg/mL, CHCl_3_), was isolated with a retention time of 20.2 min (λ = 285 nm, purity: >99%) while the dextrorotatory enantiomer, (+)-(*R*)-**3** (${\left[\alpha \right]}_{D}^{20}$ = +6.4°, c = 1.06 cg/mL, CHCl_3_), was isolated with a retention time of 24.1 min (λ = 285 nm, purity: >99%).

Compound (2S*,5R*)-**4** was purified using Hex (solvent A) and ethyl acetate (solvent B) as the mobile phase at a flow rate of 5 mL min^−1^. Solutions of (2S*,5R*)-**4** were prepared in mixtures of Hex–EtOAc (9:1, *v/v*) at concentrations of 50 mg mL^−1^ and an injection volume of 30 μL. The best separation was achieved with isocratic conditions of 2% B and a total run time of 35 min. The dextrorotatory enantiomer, (−)-(2*R*,5*S*)-**4** (${\left[\alpha \right]}_{D}^{20}$ = −4.8°, c = 0.10 cg/mL, MeOH), was isolated with a retention time of 30.8 min (λ = 285 nm, purity: 98%) while the levorotatory enantiomer, (+)-(2*S*,5*R*)-**4** (${\left[\alpha \right]}_{D}^{20}$ = +4.9°, c = 0.09 cg/mL, MeOH), was isolated with a retention time of 34.2 min (λ = 285 nm, purity: 95%).

Compound (2R*,5R*)-**4** was purified using Hex (solvent A) and EtOAc (solvent B) as the mobile phase at a flow rate of 5 mL min^−1^. Solutions of (2R*,5R*)-**4** were prepared in mixtures of Hex–EtOAc (9:1, *v*/*v*) at concentrations of 163 mg mL^−1^ and an injection volume of 15 μL. The best separation was achieved with isocratic conditions of 2% B and a total run time of 30 min. The dextrorotatory enantiomer, (+)-(2*R*,5*R*)-**4** (${\left[\alpha \right]}_{D}^{20}$ = +1.9°, c = 0.09 cg/mL, MeOH), was isolated with a retention time of 25.7 min (λ = 285 nm, purity: 99%) while the levorotatory enantiomer, (−)-(2*S*,5*S*)-**4** (${\left[\alpha \right]}_{D}^{20}$ = −1.7°, c = 0.07 cg/mL, MeOH), was isolated with a retention time of 29.6 min (λ = 285 nm, purity: 99%).

### 3.8. Esterification of (−)-***3*** with (−)-(2R)- and (+)-(2S)-MTPA Chloride (Mosher’s Method)

(−)-(2*R*)-MTPA chloride (17.7 μL, 0.095 mmol) was added to a stirred solution of (−)-**3** (11.8 mg, 0.045 mmol) and *N*,*N*-dimethylaminopyridine (DMAP) (18.2 mg, 0.149 mmol) in dry DCM (1 mL). The crude reaction had stirring maintained at room temperature (TLC monitored using Hex–Et_2_O 6:4). Once the reaction was completed, the crude was diluted with Et_2_O (5 mL), and the organic solution was washed with water (5 mL). After that, the aqueous phase was extracted twice with Et_2_O (2 × 5 mL). The organic phases were joined and dried over anh. Na_2_SO_4_, and the solvent was removed in vacuo [63]. The product obtained was purified by column chromatography (CC).

The purification step was performed by CC using mixtures of Hex–Et_2_O. With the mixture of solvents in a 95:5 ratio, pure compound **3a** (18.9 mg, 88%) was obtained as a colourless liquid.

In the same way, the (*R*)-MTPA ester (**3b**) was obtained as a colourless liquid after reacting (−)-**3** with (+)-(2*S*)-MTPA chloride, with an 80% yield.

(*S*)-(*E*)-1-(3′,4′-methylenedioxyphenyl)-6,6-dimethylhept-4-en-3-yl (2*S*)-3,3,3-trifluoro-2-methoxy-2-phenylpropanoate (**3a**) ^1^H NMR (400 MHz, CDCl_3_) δ (ppm) 7.53-7.56 (*m*, 2H, *m*-Ph), 7.36-7.43 (*m*, 3H, *o,p*-Ph), 6.71 (*d*, *J*_5′,6′_ = 8.0 Hz, 1H, H-5′), 6.58 (*d*, *J*_2′,6′_ = 1.8 Hz, 1H, H-2′), 6.53 (*dd*, *J*_6′,5′_ = 8.0 Hz, *J*_6′,2′_ = 1.8 Hz, 1H, H-6′), 5.92 (*s*, 2H, O-CH_2_-O), 5.89 (*d*, *J*_5,4_ = 15.5 Hz, 1H, H-5), 5.46 (*td*, *J*_3,4_ = 7.9 Hz, *J*_3,2_ = 5.3 Hz, 1H, H-3), 5.38 (*dd*, *J*_4,5_ = 15.5 Hz, *J*_4,3_ = 7.9 Hz, 1H, H-4), 3.55-3.56 (*m*, 3H, OMe), 2.41–2.52 (*m*, 2H, H-1), 1.81–2.02 (*m*, 2H, H-2), 1.01 (*s*, 9H, H-6a, H-6b, H-7).

(*S*)-(*E*)-1-(3′,4′-methylenedioxyphenyl)-6,6-dimethylhept-4-en-3-yl (2*R*)-3,3,3-trifluoro-2-methoxy-2-phenylpropanoate (**3b**) ^1^H NMR (400 MHz, CDCl_3_) δ (ppm) 7.52-7.50 (*m*, 2H, *m*-Ph), 7.36-7.43 (*m*, 3H, *o,p*-Ph), 6.72 (*d*, *J*_5′,6′_ = 7.8 Hz, 1H, H-5′), 6.63 (*d*, *J*_2′,6′_ = 1.8 Hz, 1H, H-2′), 6.58 (*dd*, *J*_6′,5′_ = 7.8 Hz, *J*_6′,2′_ = 1.8 Hz, 1H, H-6′), 5.92 (*s*, 2H, O-CH_2_-O), 5.83 (*dd*, *J*_5,4_ = 15.6 Hz, *J*_5,3_ = 0.9 Hz, 1H, H-5), 5.42 (*br q*, *J*_3,4_ = 7.9 Hz, 1H, H-3), 5.23 (*dd*, *J*_4,5_ = 15.6 Hz, *J*_4,3_ = 7.9 Hz, 1H, H-4), 3.57-3.58 (*m*, 3H, OMe), 2.49–2.62 (*m*, 2H, H-1), 1.85–2.08 (*m*, 2H, H-2), 0.99 (*s*, 9H, H-6a, H-6b, H-7).

### 3.9. Computational Details of ECD Calculation

The conformational analysis of the four enantiomers of compound **4** was performed in the Schrödinger Macromodel 2018 software using the Monte Carlo Minimum method (MCMM). Subsequently, conformers were fully optimized in Gaussian 16 [64] with a functional/basic set combination of B3LYP/6-311 + g(2d,p). Then, the time-dependent density functional (TD-DFT) methodology was used to determine values of energies, oscillator strengths, and rotational strengths associated with 20 electronic excitations at the B3LYP/6-311 + g(2d,p) level. Solvent’s influence (MeOH) was considered in the calculation, using the polarizable continuum model (PCM) [65]. Theorical ECD spectra were plotted by SpecDis software (version 1.7.1) [66] with a half-bandwidth of 0.33 eV. The simulations were performed on the Luxembourg national supercomputer MeluXina. The experimental results of the ECD analysis were as follows:

(−)-(2*R*,5*S*)-**4**: ECD (c = 3.6 × 10^−4^ M, MeOH) λ_max_ (Δε) 224 (−7.35), 272 (−2.69), 358 (−2.00) nm;

(+)-(2*S*,5*R*)-**4**: ECD (c = 3.6 × 10^−4^ M, MeOH) λ_max_ (Δε) 224 (+5.34), 272 (+0.98), 358 (+0.74) nm;

(−)-(2*S*,5*S*)-**4**: ECD (c = 3.6 × 10^−4^ M, MeOH) λ_max_ (Δε) 226 (−6.15), 264 (−3.01), 358 (−2.69) nm;

(+)-(2*R*,5*R*)-**4**: ECD (c = 3.6 × 10^−4^ M, MeOH) λ_max_ (Δε) 226 (+5.68), 264 (+2.12), 358 (+2.03) nm.

### 3.10. Human Lactate Dehydrogenase a Enzymatic Activity Assay

The ability of the synthesized compounds to inhibit the *h*LDHA enzyme was measured using recombinant human LDHA (95%, specific activity >300 units/mg and concentration of 0.5 mg/mL, Abcam, Cambridge, UK) with sodium pyruvate (96%, Merck) as a substrate and β-NADH (≥97%, Merck) as a cofactor in a potassium phosphate buffer (100 mM, pH 7.3). The enzymatic assay was conducted on 96-well microplates and a decrease in the β-NADH fluorescence (λ_excitation_ = 340 nm; λ_emission_ = 460 nm) was detected in a TECAN Infinite 200 Pro M Plex fluorescent plate reader (Tecan Group Ltd., Männedorf, Switzerland) at 28 °C. The activity was determined according to a protocol reported in the literature [67] and modified by us [33,37]. The final volume in each well was set to 200 μL using 100 mM potassium phosphate buffer, 0.041 units/mL LDHA, 155 μM β-NADH, 1 mM pyruvate (saturated conditions), and DMSO solutions (5%, *v*/*v*) of pure compounds. The addition of pyruvate allowed the for the beginning of the reaction, and fluorescence was registered every 60 s during 10 min. A lineal time interval was selected to calculate the slope at every single concentration. Controls for the establishment of the 0% and 100% enzymatic activity were introduced in the assay. In the case of the 0% control, buffer was added instead of piruvate, whilst for the 100% control, no inhibitor was added. The compound 3-[[3[(cyclopropylamino)sulfonyl]-7-(2,4-dimethoxy-5-pyrimidinyl)-4-quinolinyl]amino]-5-(3,5-difluorophenoxy)benzoic acid (GSK 2,837,808A, Tocris, MN, USA) was used as a positive control at 1 μM in the well [68]. The slope obtained at each concentration was compared to the one obtained for the 100% enzymatic activity control to determine the corresponding enzymatic activity. A nonlinear regression analysis in GraphPad Prism version 9.00 for Windows (GraphPad Software, La Jolla, CA, USA) was used for the dose–response curve, for the fitting of the logarithm of inhibitor concentration vs. normalized enzymatic activity, to calculate IC_50_ values (see Figures S65–S74 in the Supplementary Materials). All measurements were made in triplicate and data were expressed as the mean ± SD.

## 4. Conclusions

The present work reports on the finding that three novel compounds, structurally related to stiripentol (STP), are capable of inhibiting human lactate dehydrogenase A (*h*LDHA) significantly more extensively than STP. Taking into account that the inhibition of *h*LDHA has already been validated as a safe therapeutic method to treat primary hyperoxaluria (PH) and that STP is currently being studied as a candidate to become a new drug for PH in the future, further efforts should be made to understand whether these analogues of STP increase, or do not increase, the proven ability of STP to reduce urinary oxalate levels in vivo. In parallel, new STP-related compounds should be designed using virtual docking screening in order to lower the best IC_50_ value found (ca. 100 μM) in this work.

## Figures and Tables

**Table 1:** Differences in the ^1^H NMR chemical shifts in the (*S*) and (*R*)-MTPA esters of (−)-**3**, to define the stereochemistry at C-3.

Proton	*δ*(*S*) (Ester 3a) (ppm)	*δ*(*R*) (Ester 3b) (ppm)	∆*δ*^SR^ = *δ*(S) − *δ*(R)	
			ppm	Hz (400 MHz)
**O-CH_2_-O**	5.92	5.92	0	0
**2′**	6.58	6.63	−0.05	−20
**5′**	6.71	6.72	−0.01	−4
**6′**	6.53	6.58	−0.05	−20
**1**	2.41–2.52 (2.47)	2.49–2.62 (2.56)	−0.09	−36
**2**	1.81–2.02 (1.92)	1.85–2.08 (1.97)	−0.05	−20
**3**	5.46	5.42	+0.04	+16
**4**	5.38	5.23	+0.15	+60
**5**	5.89	5.83	+0.06	+24
**6a, 6b, 7**	1.01	0.99	+0.02	+8

**Table 2:** Percentages of inhibition for racemics **1**–**5** and enantiomers of **1**, and IC_50_ values for racemics **3**–**5** and enantiomers of **3** and **4** against *h*LDHA.

Compound	% Inhibition ^a^ (*h*LDHA)	IC_50_ (μM) ^b^ (*h*LDHA)	R^2^
Stiripentol ((±)-**1**)	9.9 ± 2.3	>500	
(−)-(*S*)-**1**	34.0 ± 2.1	>500	
(+)-(*R*)-**1**	38.4 ± 1.6	>500	
iso-Stiripentol ((±)-**2**)	21.1 ± 3.5	>500	
(±)-**3**	70.0 ± 3.0	125.3 ± 19.0	0.98
(−)-(*S*)-**3**	ND ^c^	213.7 ± 13.9	0.96
(+)-(*R*)-**3**	ND ^c^	166.3 ± 16.9	0.99
(±)-(2S*,5R*)-**4**	56.3 ± 8.2	199.8 ± 23.8	0.98
(−)-(2*R*,*5S*)-**4**	ND ^c^	101.7 ± 6.7	0.99
(+)-(2*S*,*5R*)-**4**	ND ^c^	128.4 ± 12.4	0.97
(±)-(2R*,5R*)-**4**	71.7 ± 2.2	129.6 ± 15.3	0.98
(+)-(2*R*,*5R*)-**4**	ND ^c^	179.7 ± 10.5	0.95
(−)-(2*S*,*5S*)-**4**	ND ^c^	149.3 ± 10.4	0.97
(±)-**5**	46.4 ± 2.8	200.5 ± 13.9	0.98

^a^ Percentage of inhibition values are presented as the mean ± SD of *n* = 4 replicates at a single concentration of 500 μM. ^b^ IC_50_ values are presented as the mean ± SD of *n* = 3 replicates, evaluated at eight different concentrations, in a range of 0.46–1000 μM. ^c^ Not determined.

## Data Availability

Data are contained within the article and Supplementary Materials.

## Supplementary Materials

The following supporting information can be downloaded at https://www.mdpi.com/article/10.3390/ijms252413266/s1.
