# Supplementary material for: Synthesis and hLDHA Inhibitory Activity of New Stiripentol-Related Compounds of Potential Use in Primary Hyperoxaluria
Source: Int J Mol Sci. 2024 Dec 10;25(24):13266. doi: 10.3390/ijms252413266 (PMC11675970; doi:10.3390/ijms252413266)
Supplement: Supplementary file 1 [file ijms-25-13266-s001.zip › ijms-3339511-supplementary.pdf]

SUPPLEMENTARY MATERIAL

Synthesis and *h*LDHA Inhibitory Activity of New Stiripentol-Related Compounds of Potential Use in Primary Hyperoxaluria

Mario Rico-Molina [1], Juan Ortega-Vidal [1,2], Juan Molina-Canteras [1], Justo Cobo [1], Joaquín Altarejos [1,*] and Sofía Salido [1]

[1] Department of Inorganic and Organic Chemistry, Faculty of Experimental Sciences, University of Jaén, Campus of International Excellence in Agri-Food (ceiA3), 23071 Jaén, Spain; mrico@ujaen.es (M.R.-M.); jovidal@ujaen.es (J.O.-V.); jcantera@ujaen.es (J.M.-C.); jcobo@ujaen.es (J.C.); ssalido@ujaen.es (S.S.)

[2] School of Biological and Chemical Sciences, Ryan Institute, University of Galway, H91TK33 Galway, Ireland; juan.ortegavidal@universityofgalway.ie (J.O.-V.)

* Correspondence: jaltare@ujaen.es

Table of contents

1. **NMR spectra** **p. 2**
2. **High resolution mass spectra** **p. 30**
3. **HPLC chromatograms** **p. 37**
4. **Quiral HPLC chromatograms** **p. 39**
5. **DFT-optimized lowest energy conformers** **p. 42**
6. **Dose response curves against *h*LDHA** **p. 44**

1. NMR spectra

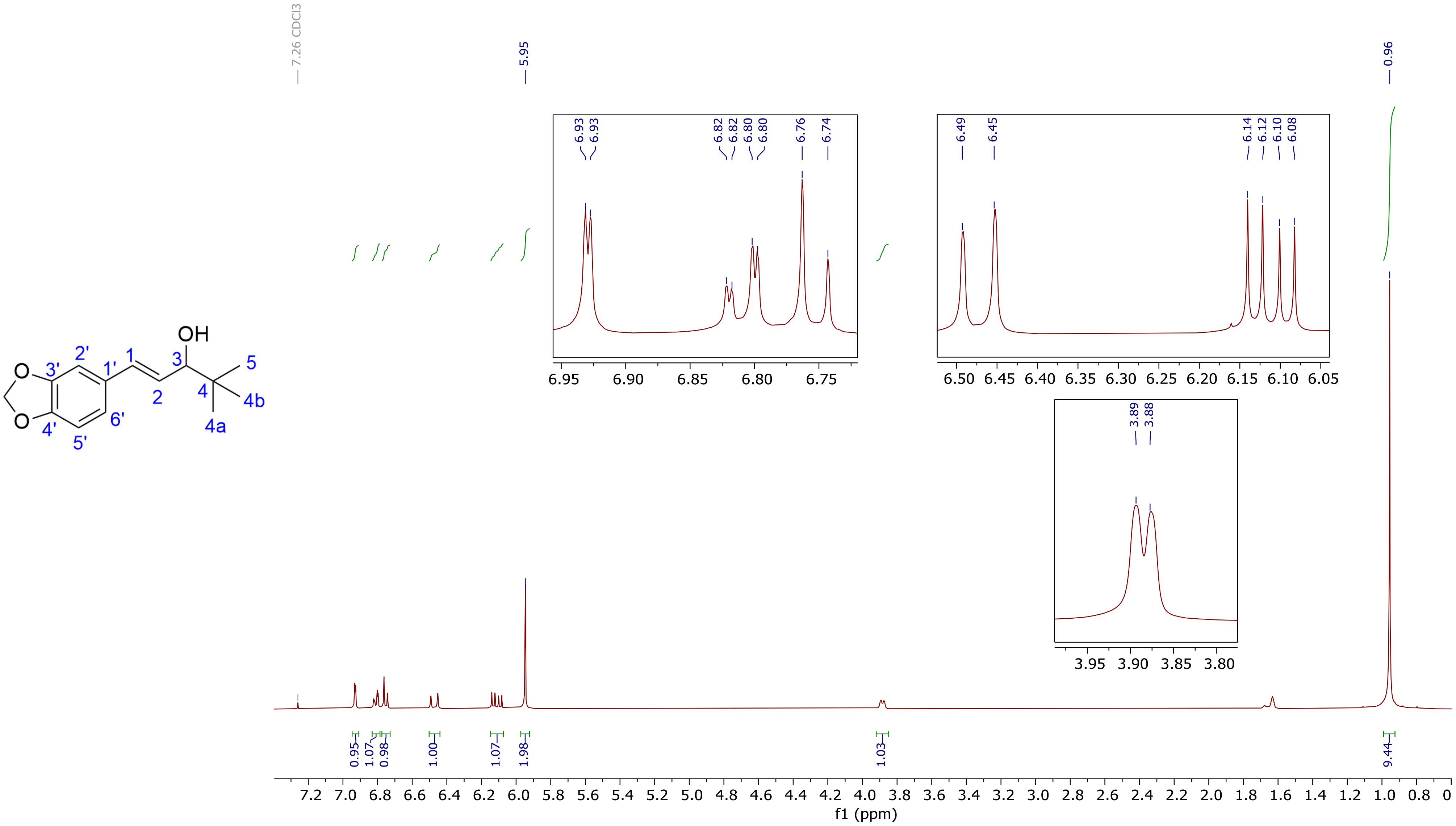

Figure S1. ^{1}H NMR spectrum (400 MHz) of compound **1** in $CDCl_3$.

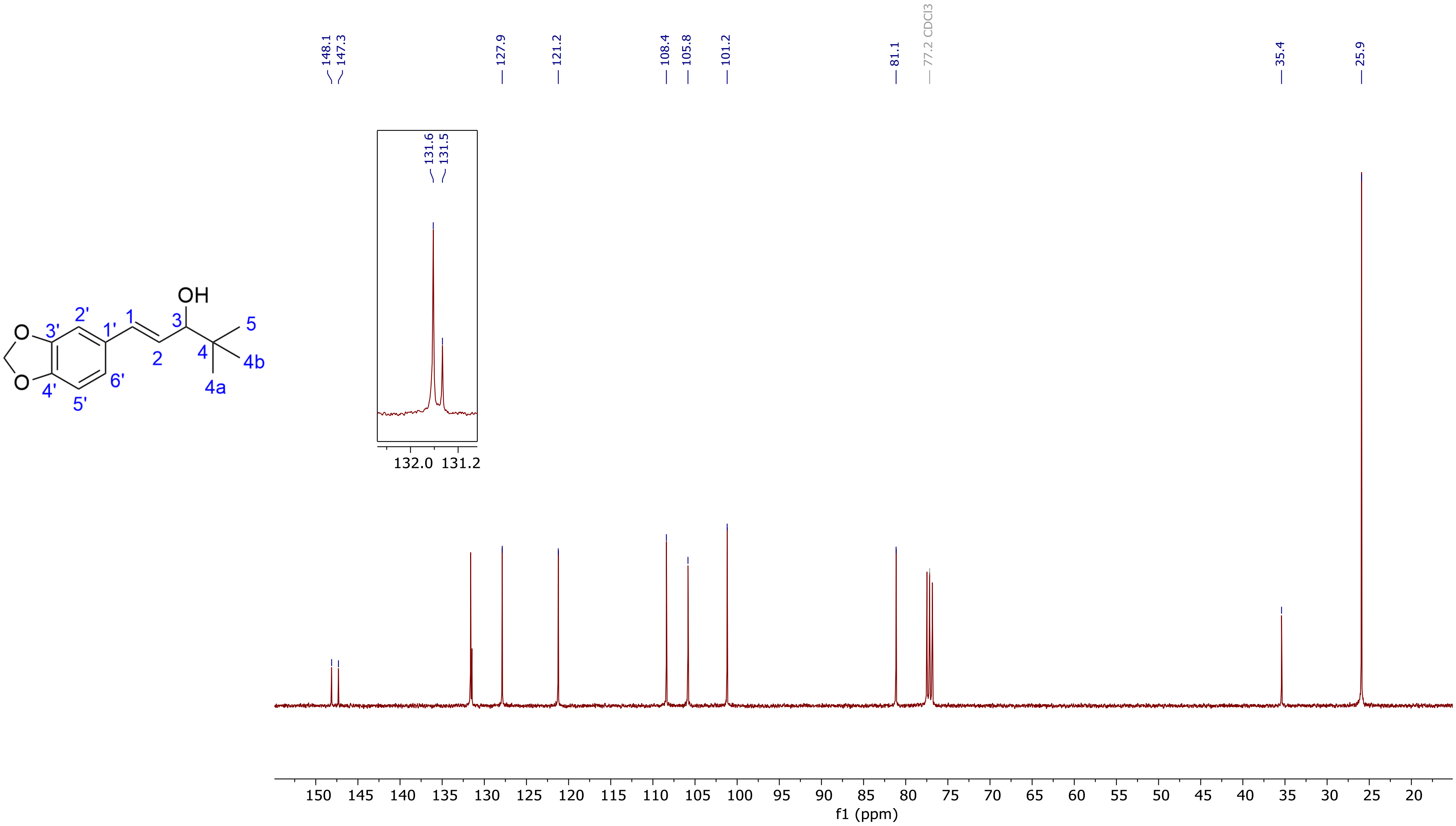

Figure S2. ^{13}C NMR spectrum (100 MHz) of compound **1** in $CDCl_3$.

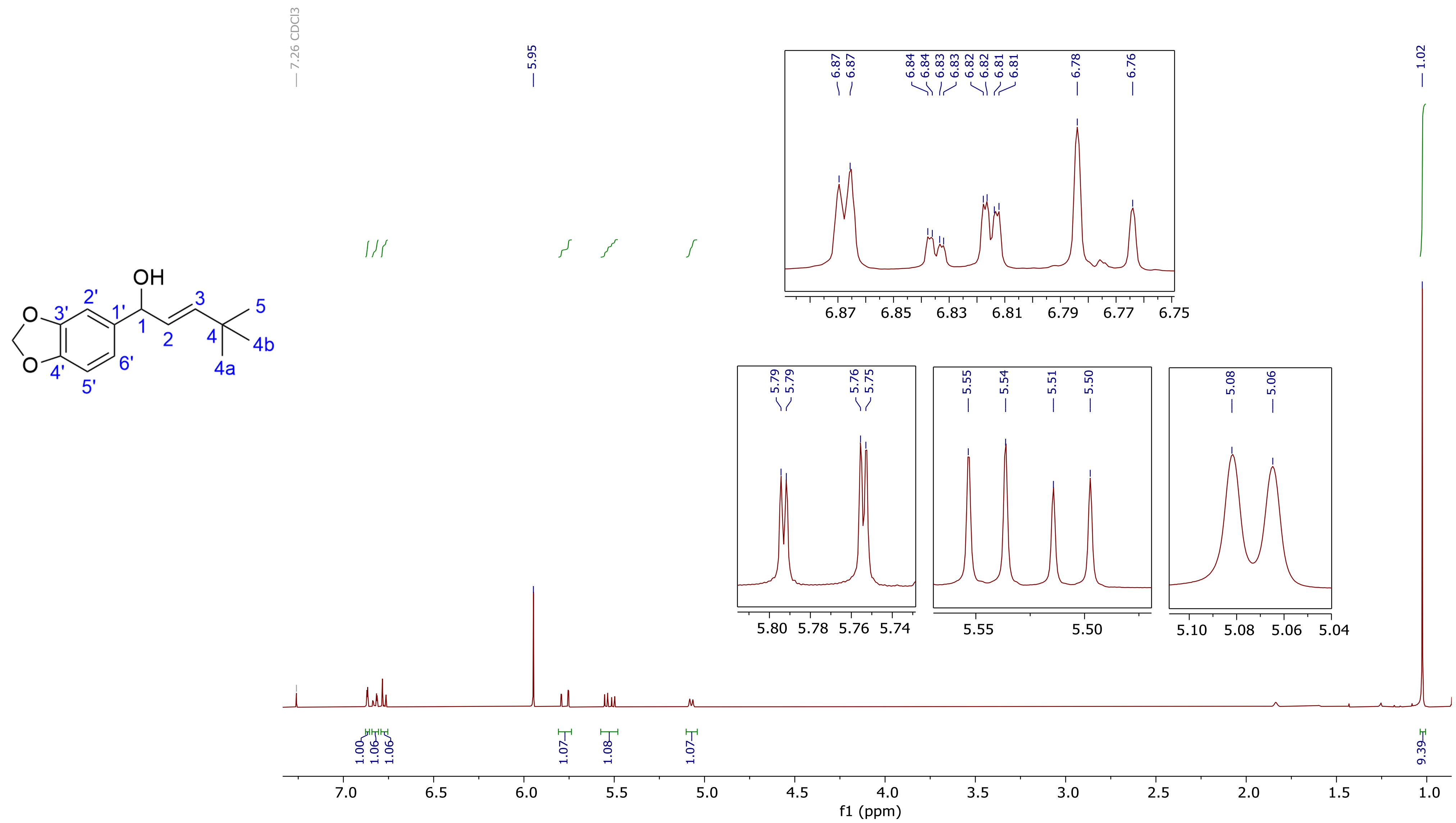

Figure S3. ^{1}H NMR spectrum (400 MHz) of compound **2** in $CDCl_3$.

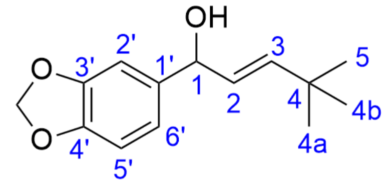

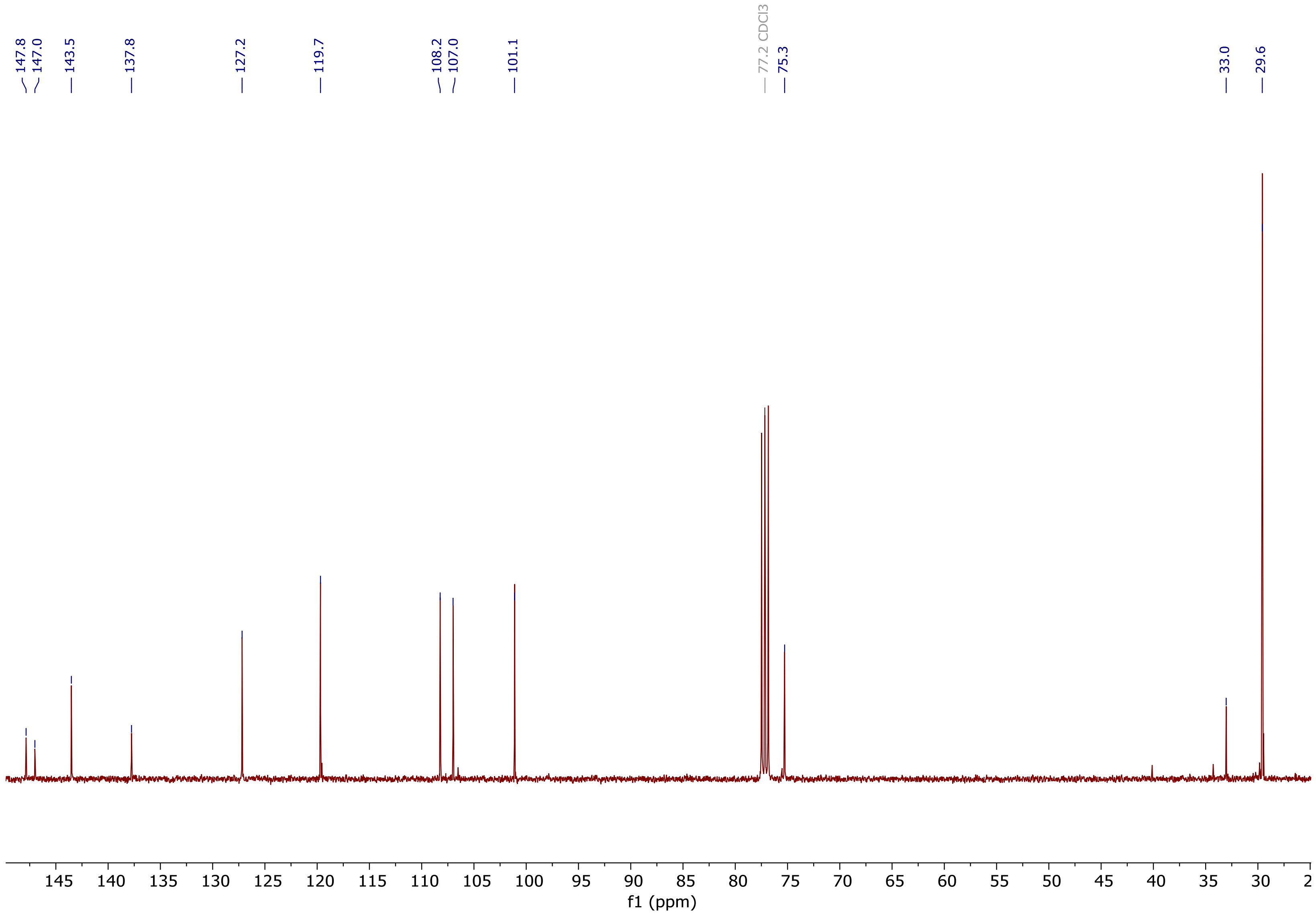

Figure S4. ^{13}C NMR spectrum (100 MHz) of compound **2** in $CDCl_3$.

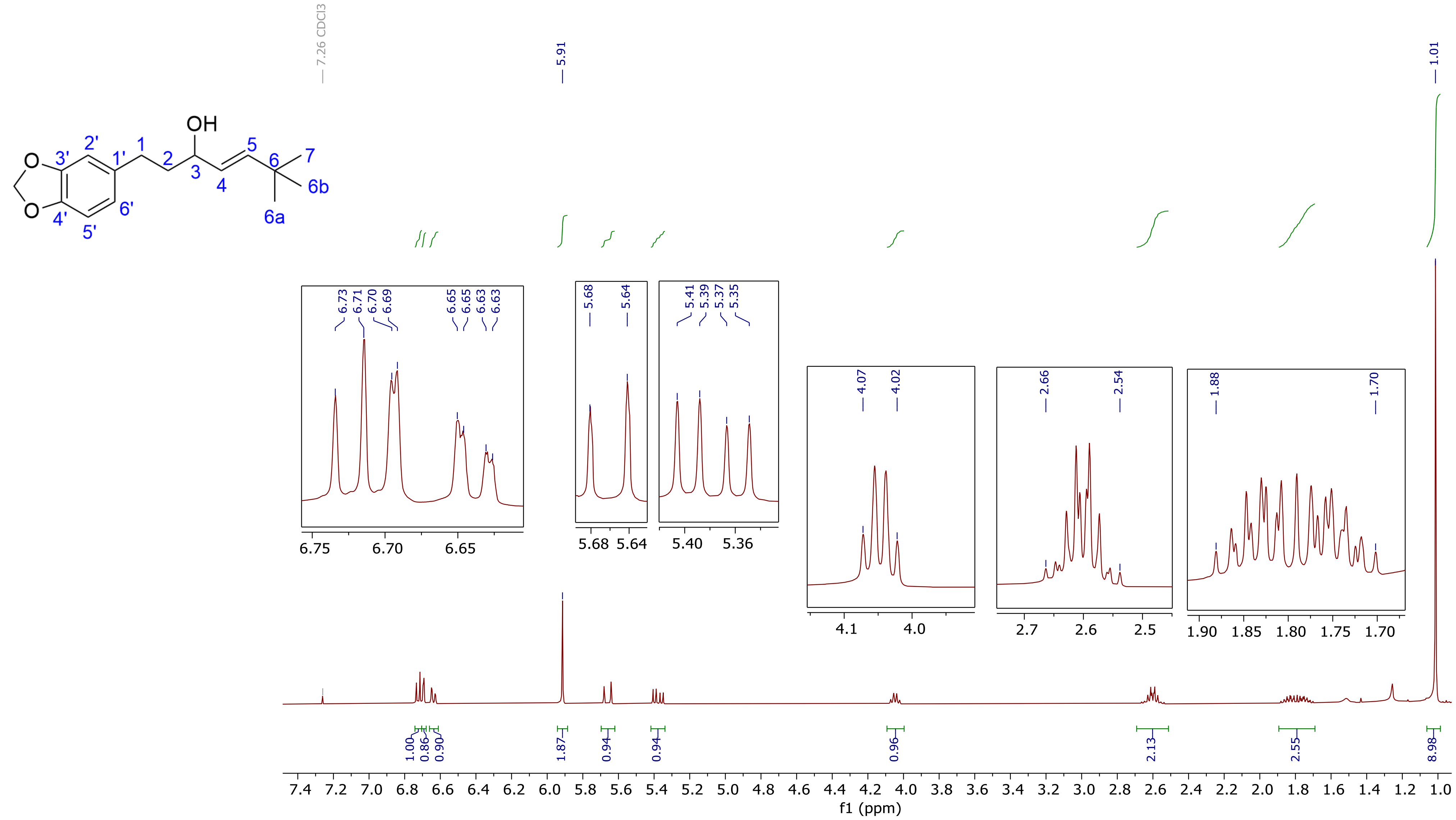

Figure S5. ^{1}H NMR spectrum (400 MHz) of compound **3** in $CDCl_3$.

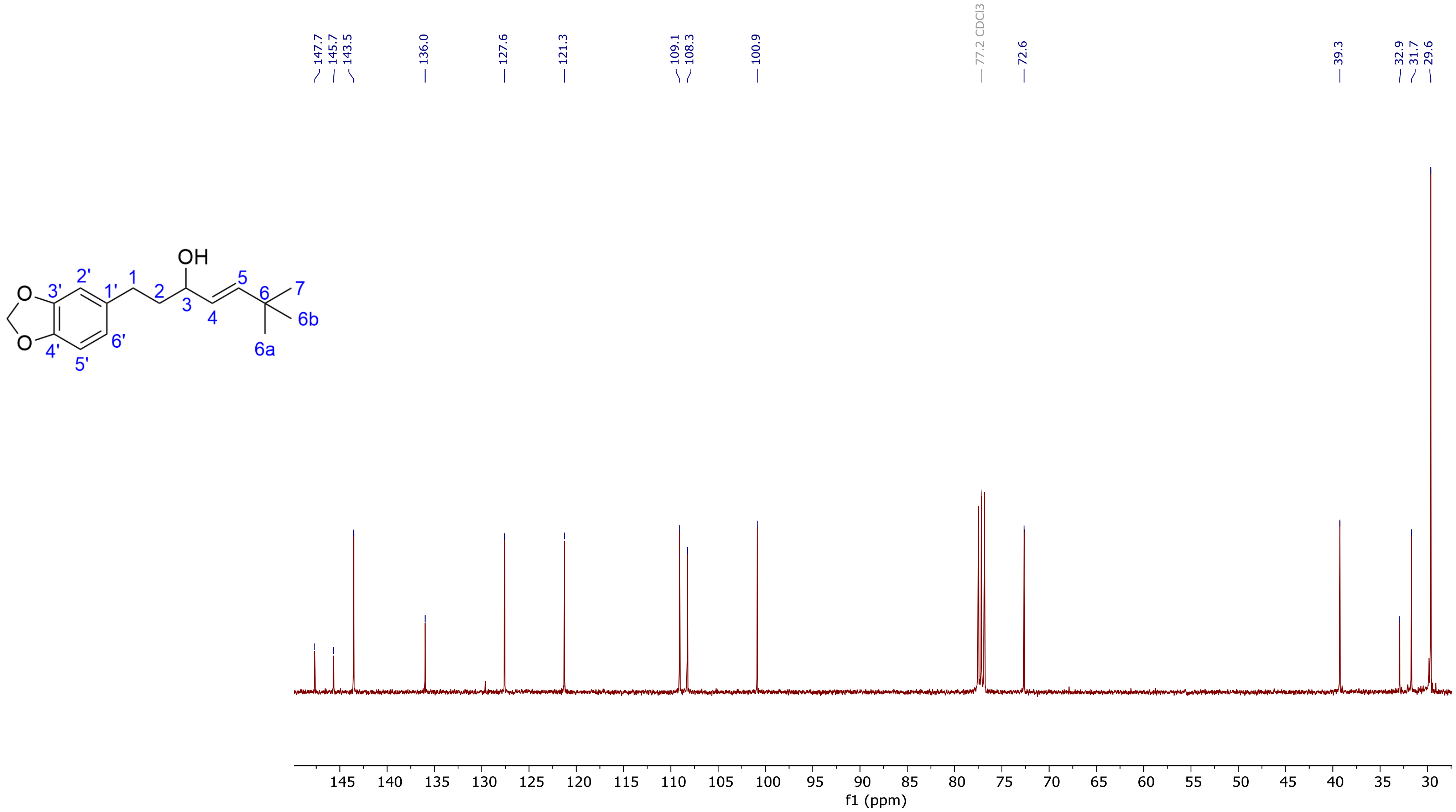

Figure S6. ^{13}C NMR spectrum (100 MHz) of compound **3** in $CDCl_3$.

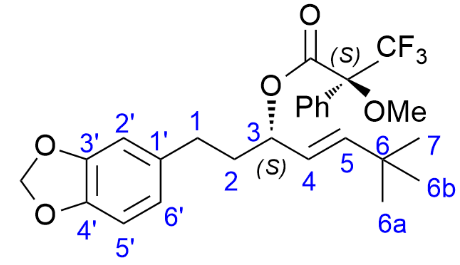

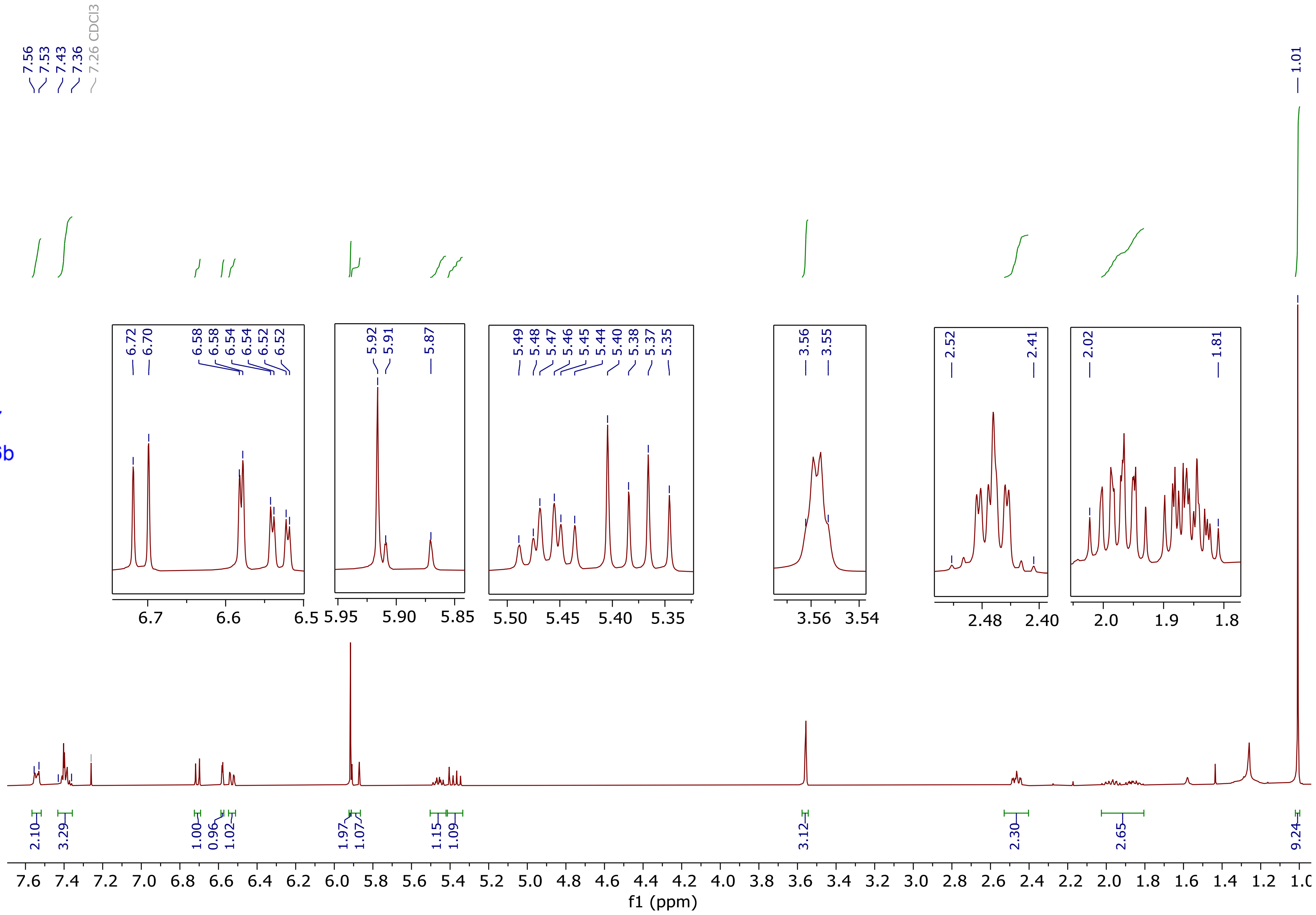

Figure S7. ^{1}H NMR spectrum (400 MHz) of compound **3a** in $CDCl_3$.

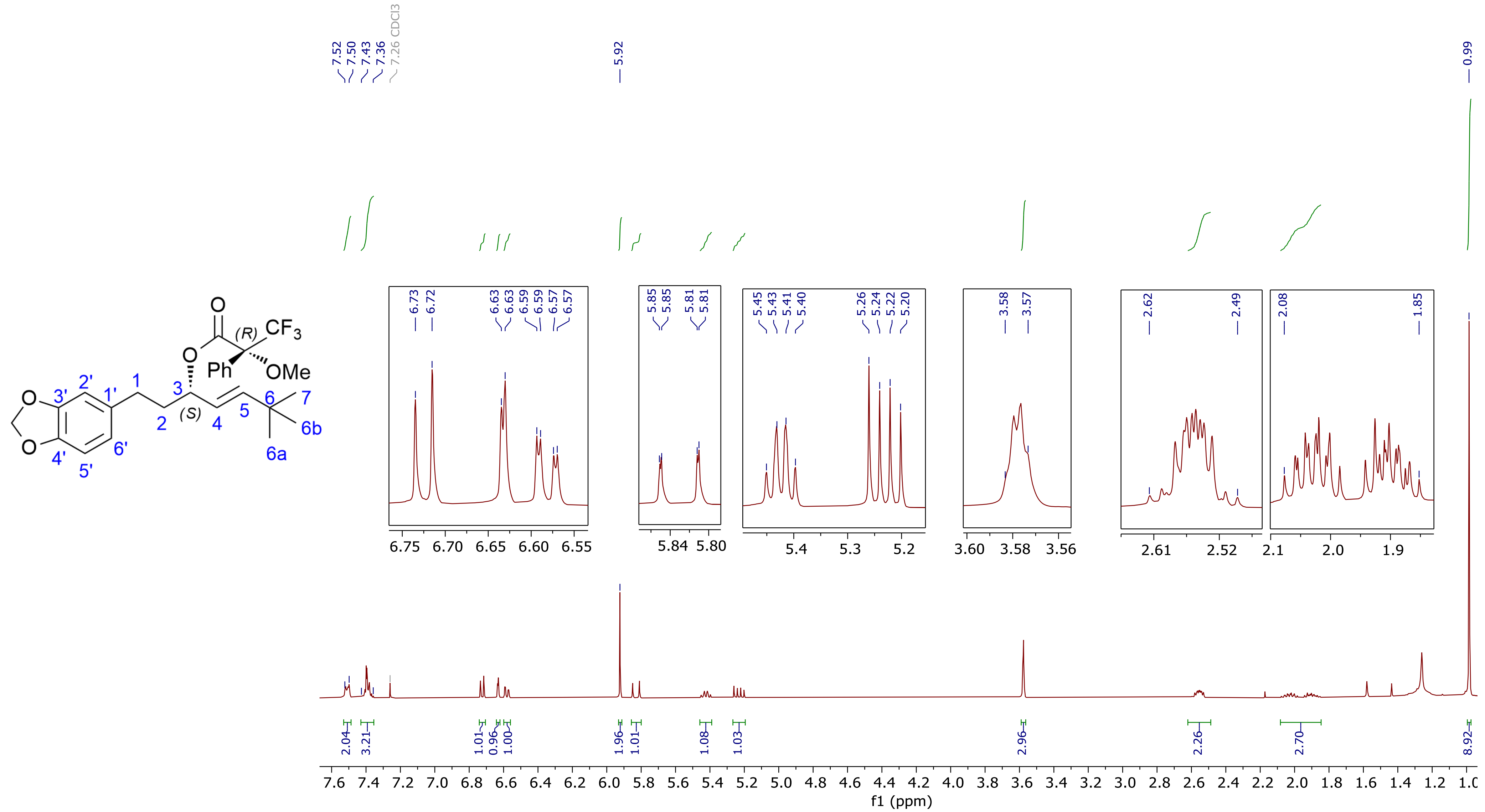

Figure S8. ^1H NMR spectrum (400 MHz) of compound **3b** in $CDCl_3$.

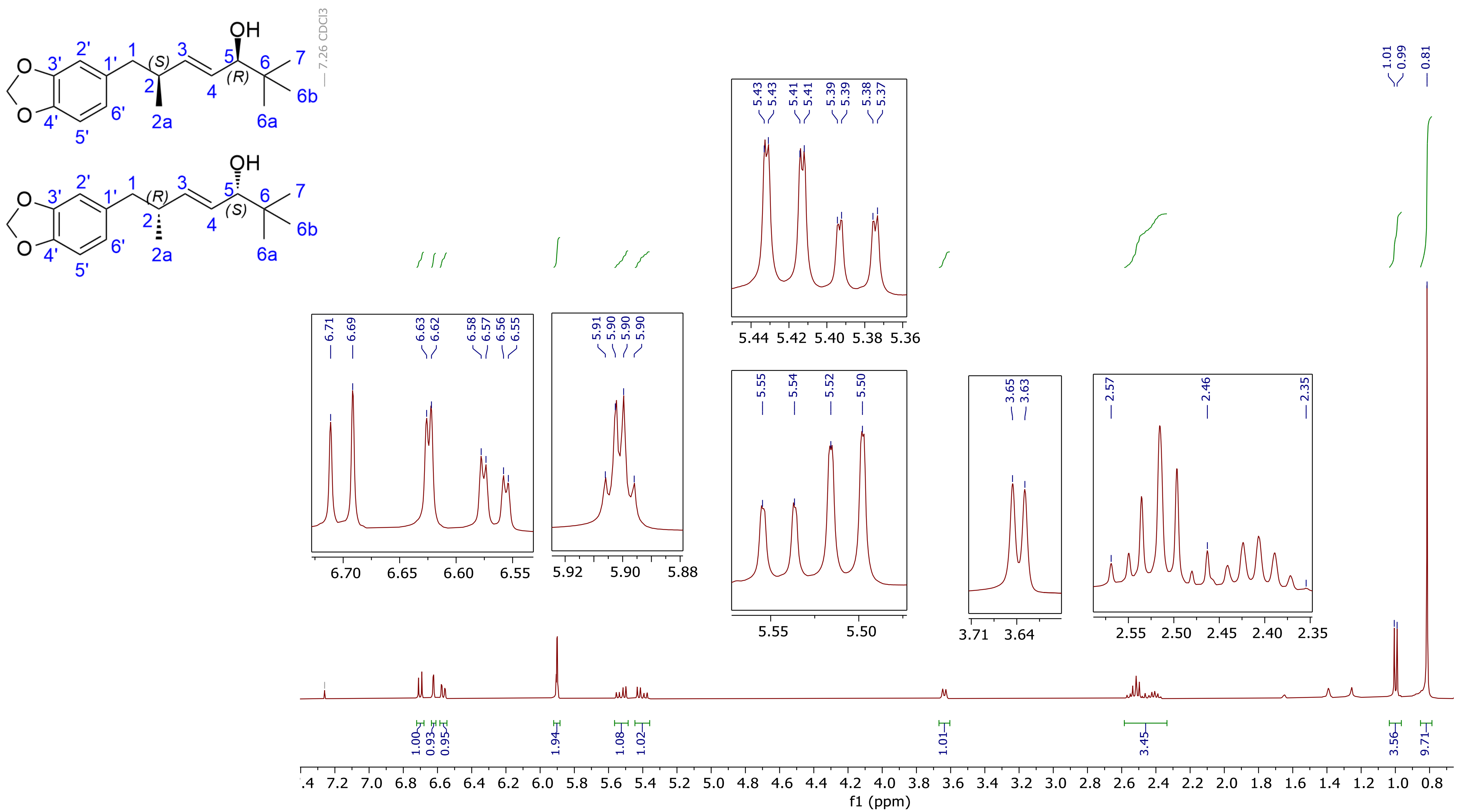

Figure S9. ^{1}H NMR spectrum (400 MHz) of compound (*2S*,5R**)-**4** in $CDCl_3$.

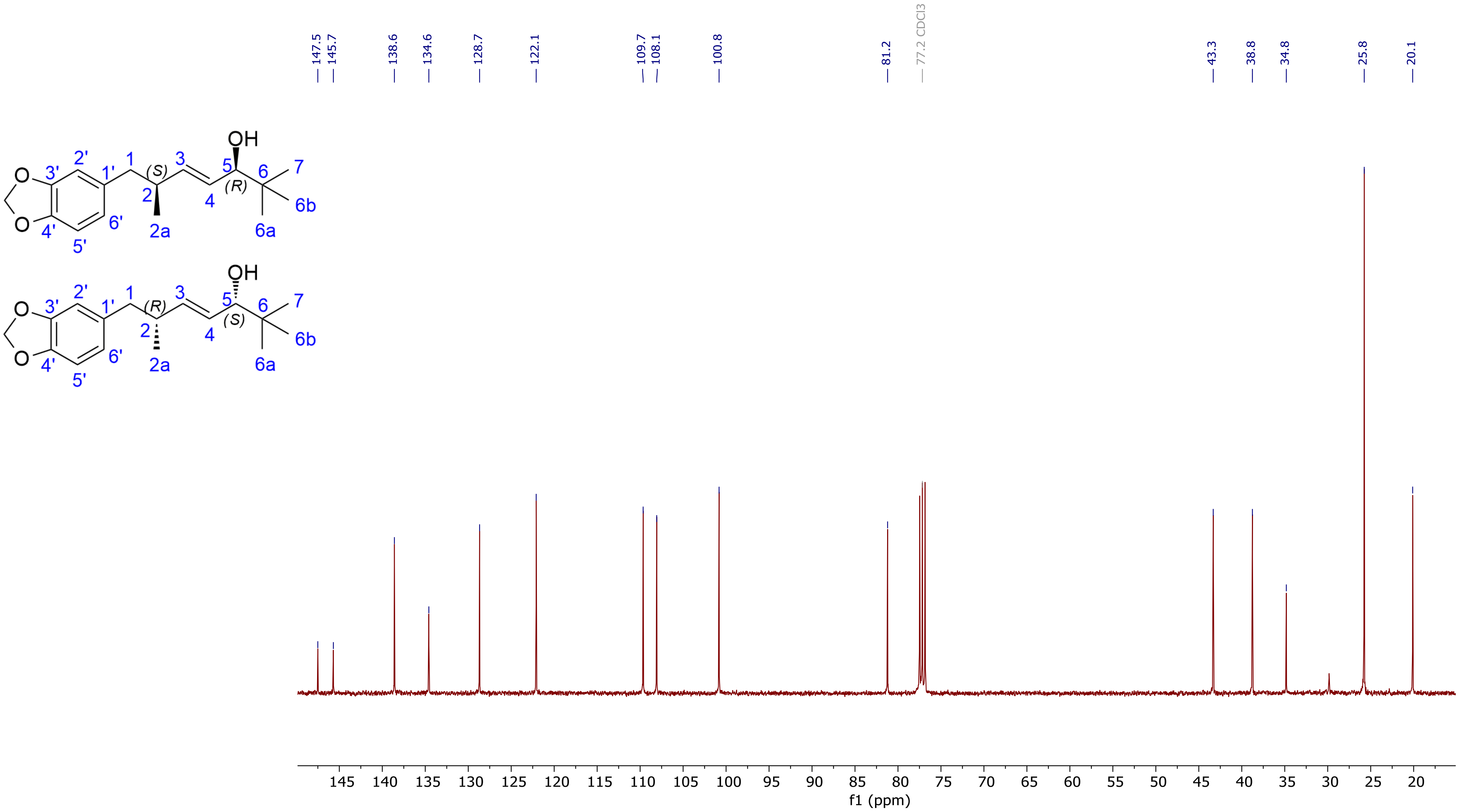

Figure S10. ^{13}C NMR spectrum (100 MHz) of compound (2*S**,5*R**)-**4** in $CDCl_3$.

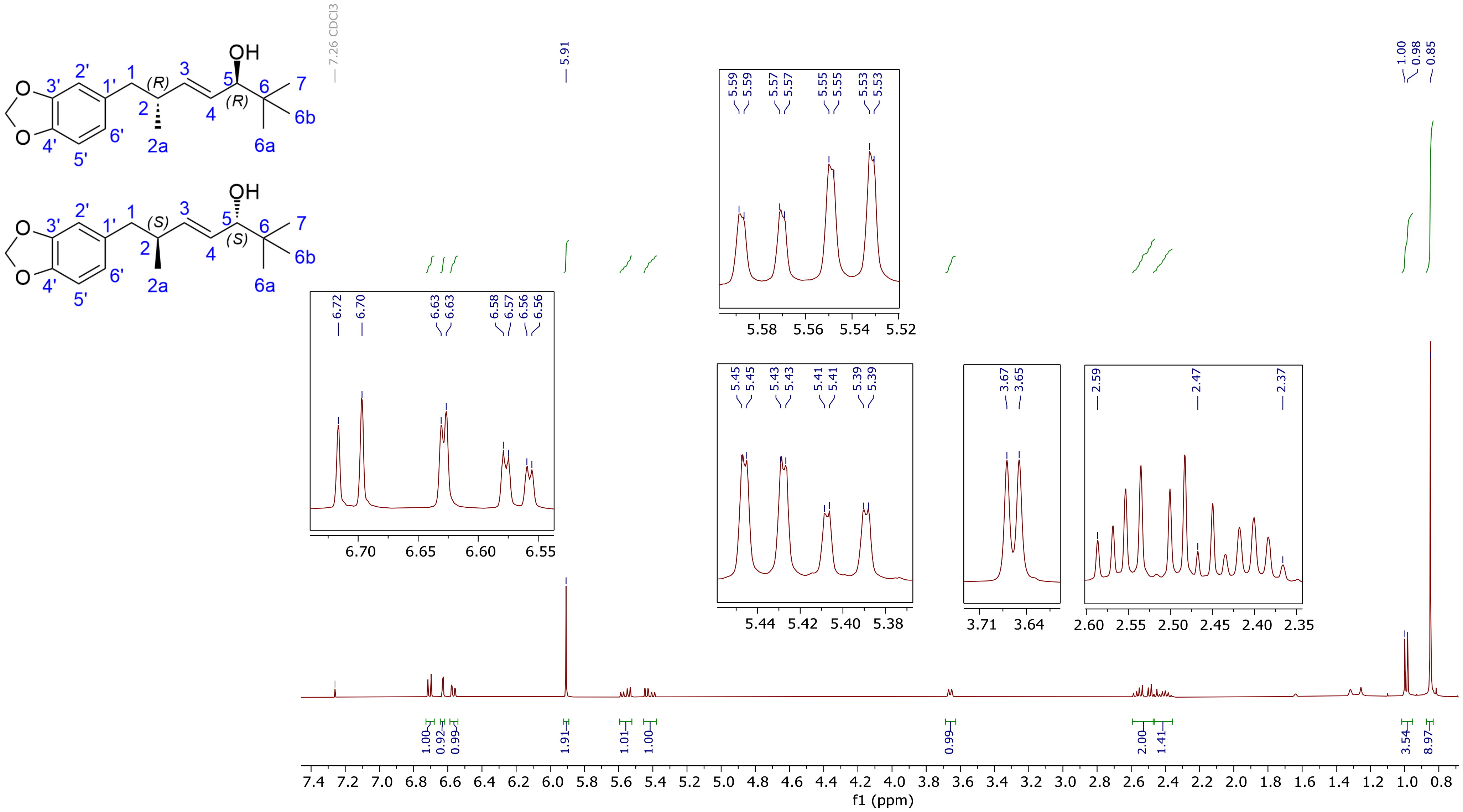

Figure S11. ^{1}H NMR spectrum (400 MHz) of compound (2*R**,5*R**)-**4** in CDCl3.

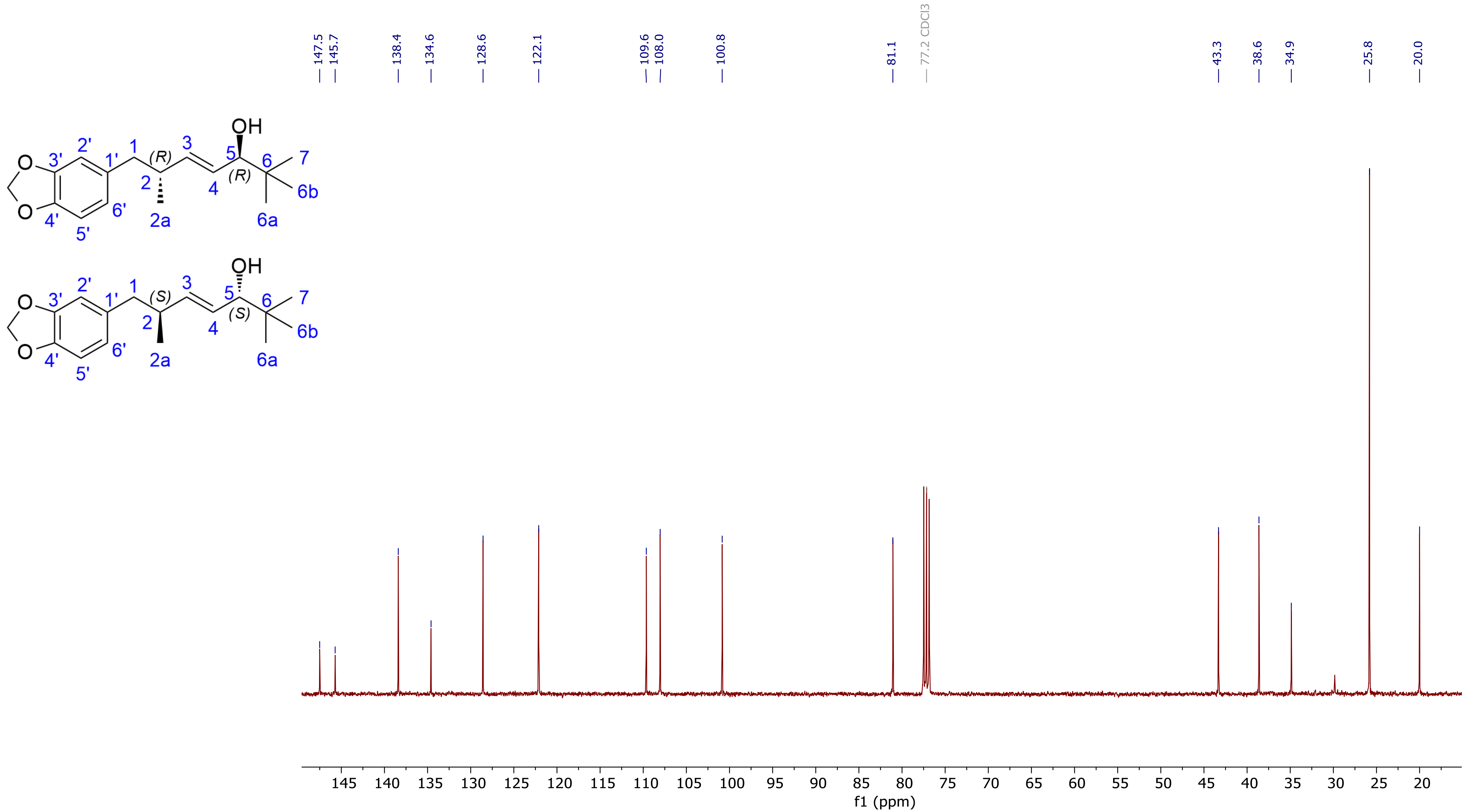

Figure S12. ^{13}C NMR spectrum (100 MHz) of compound (2*R**,5*R**)-**4** in $CDCl_3$.

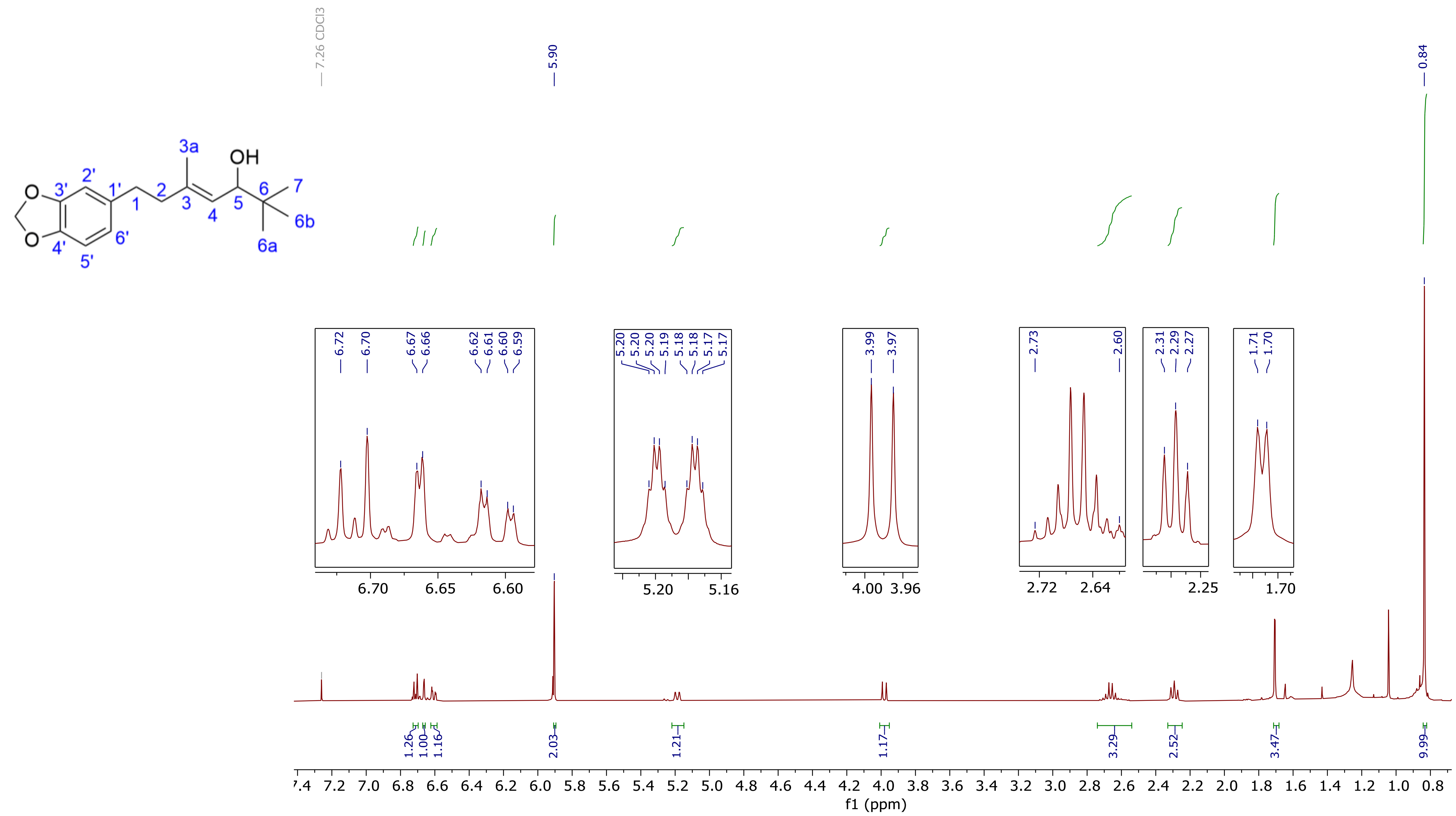

Figure S13. ^{1}H NMR spectrum (400 MHz) of compound **5** in $CDCl_3$.

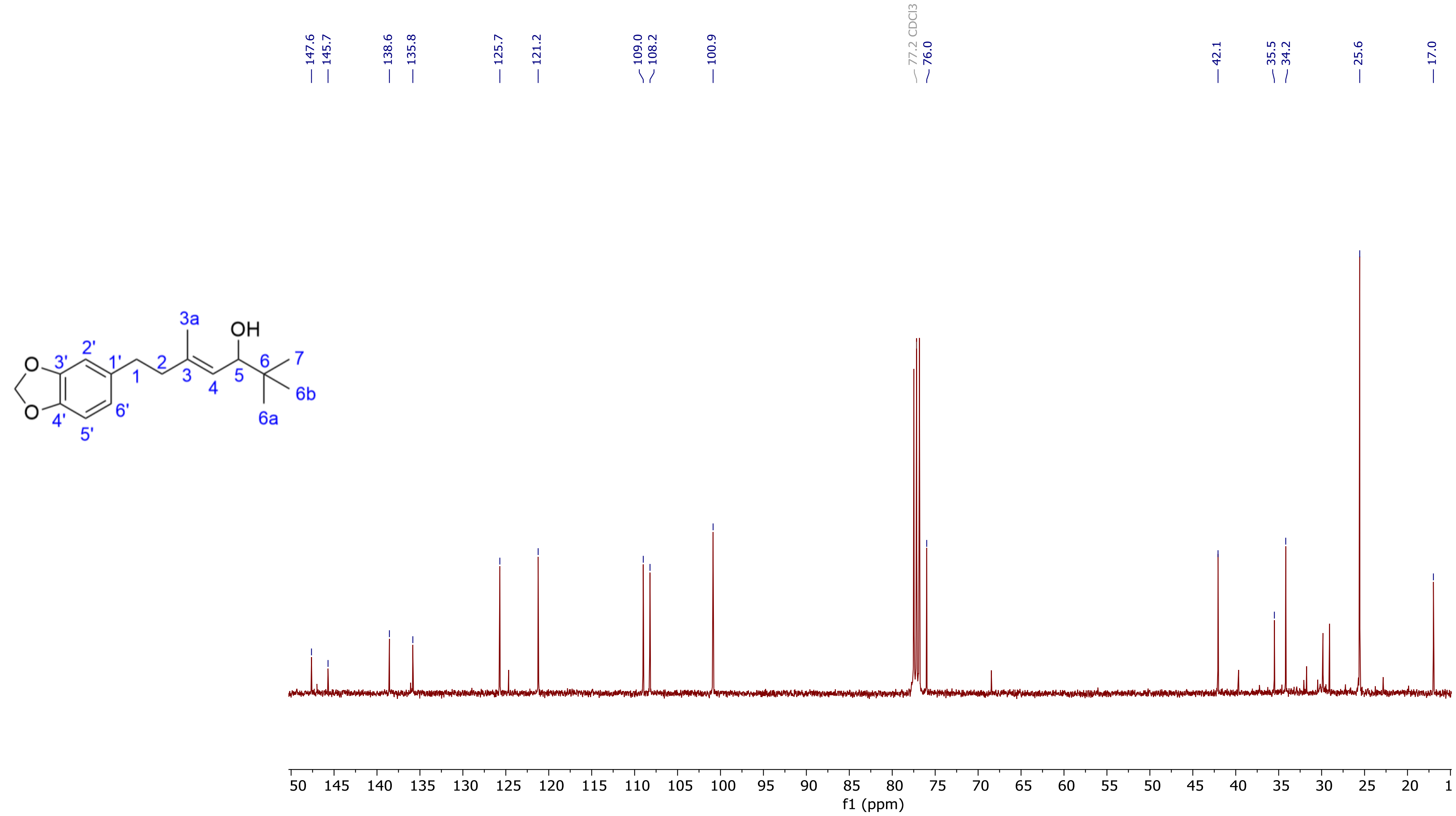

Figure S14. ^{13}C NMR spectrum (100 MHz) of compound **5** in $CDCl_3$.

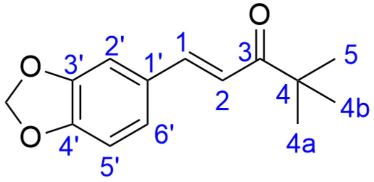

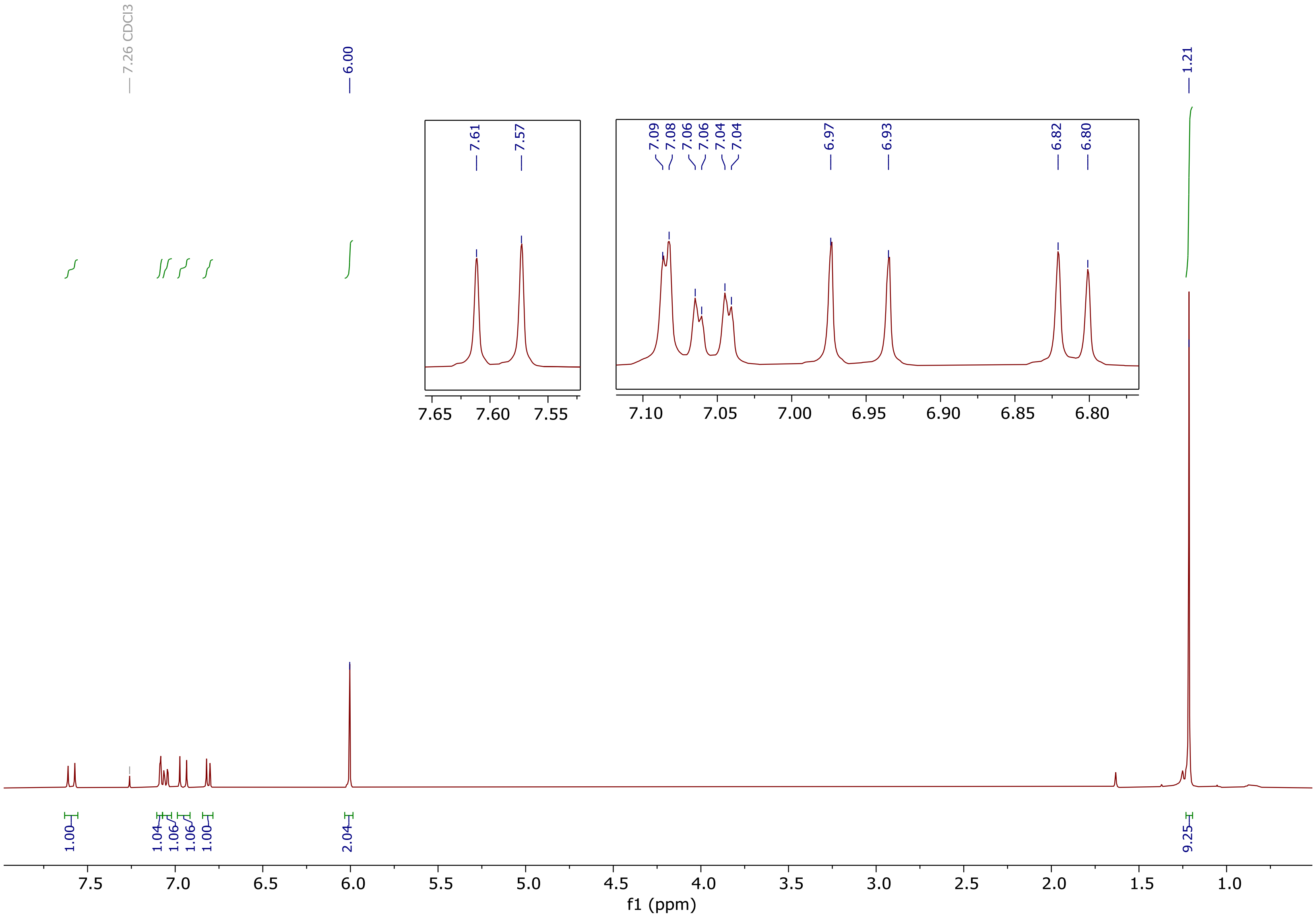

Figure S15. ^{1}H NMR spectrum (400 MHz) of compound **8** in $CDCl_3$.

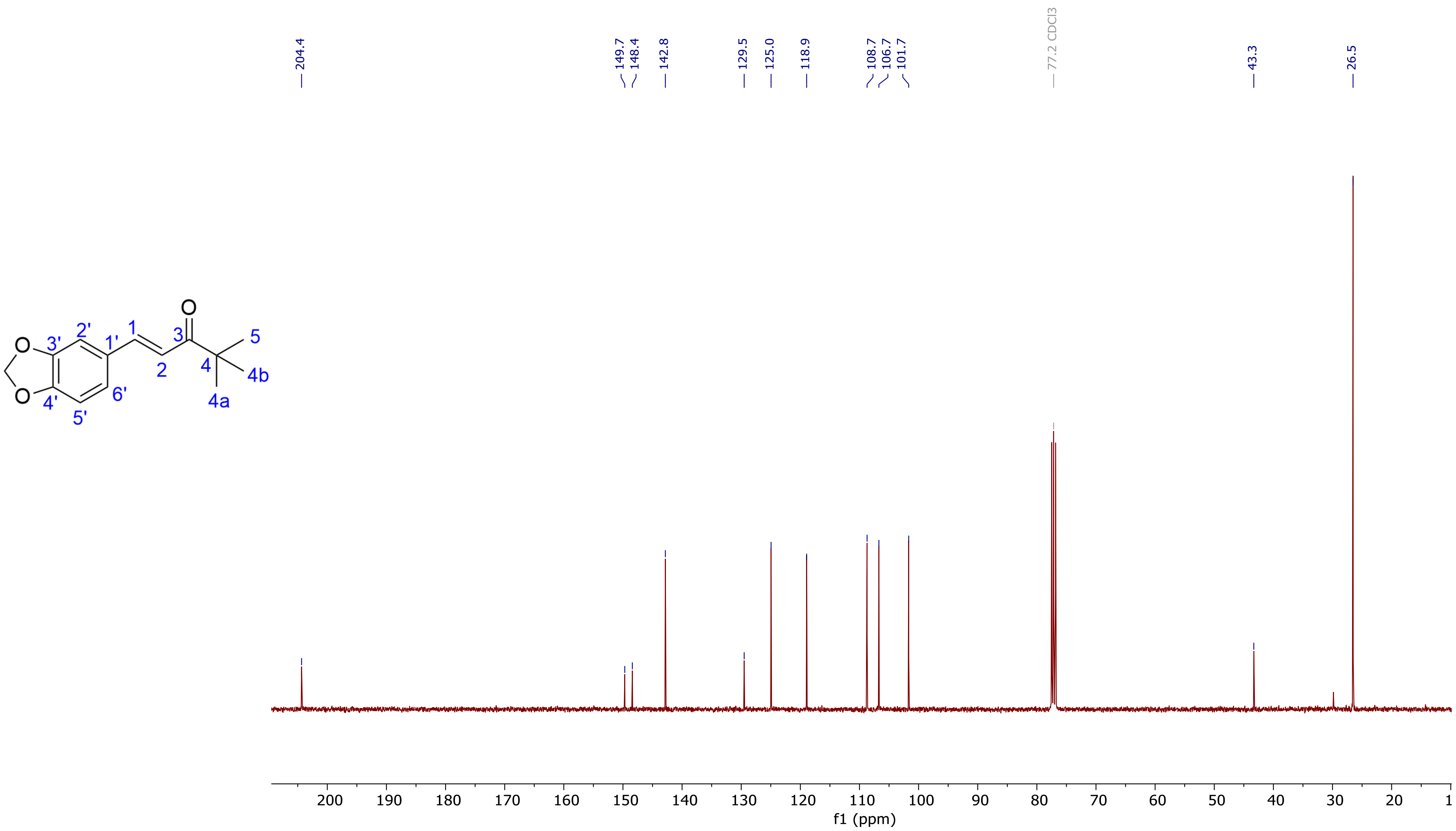

Figure S16. ^{13}C NMR spectrum (100 MHz) of compound **8** in $CDCl_3$.

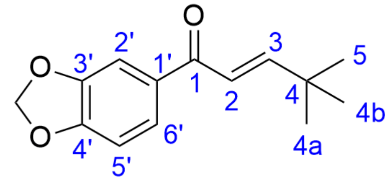

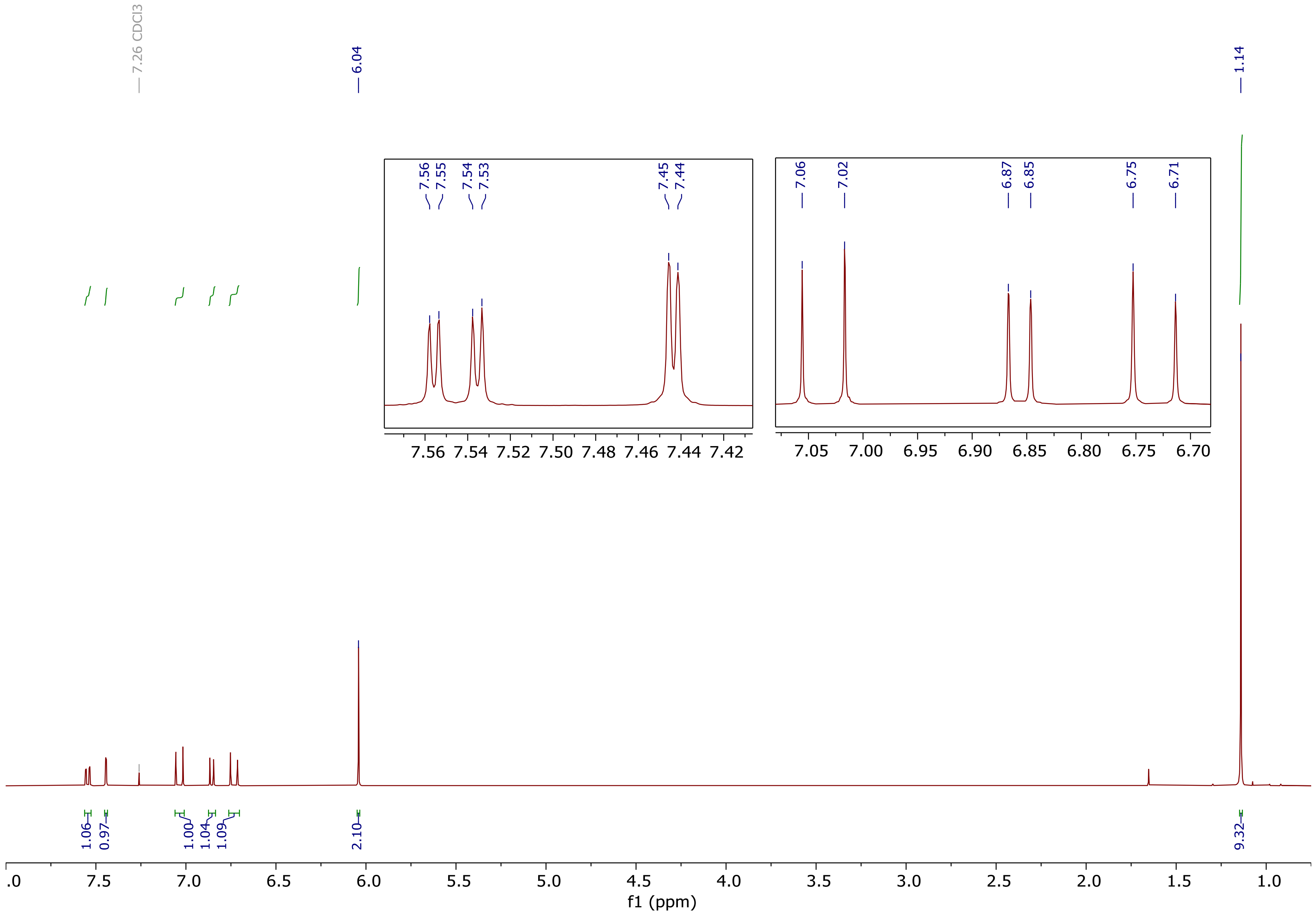

Figure S17. ^{1}H NMR spectrum (400 MHz) of compound **11** in $CDCl_3$.

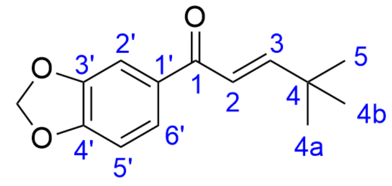

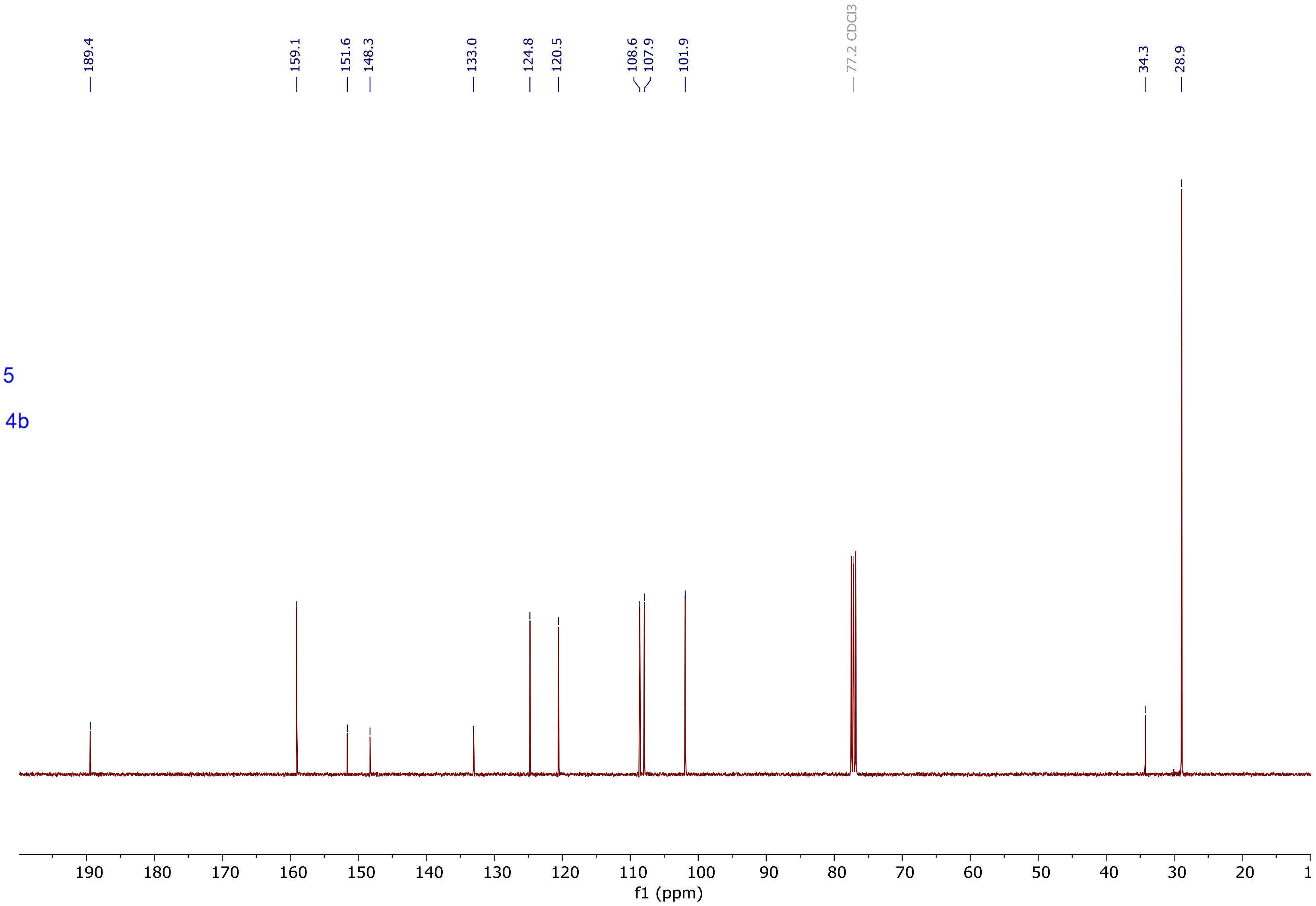

Figure S18. ^{13}C NMR spectrum (100 MHz) of compound **11** in $CDCl_3$.

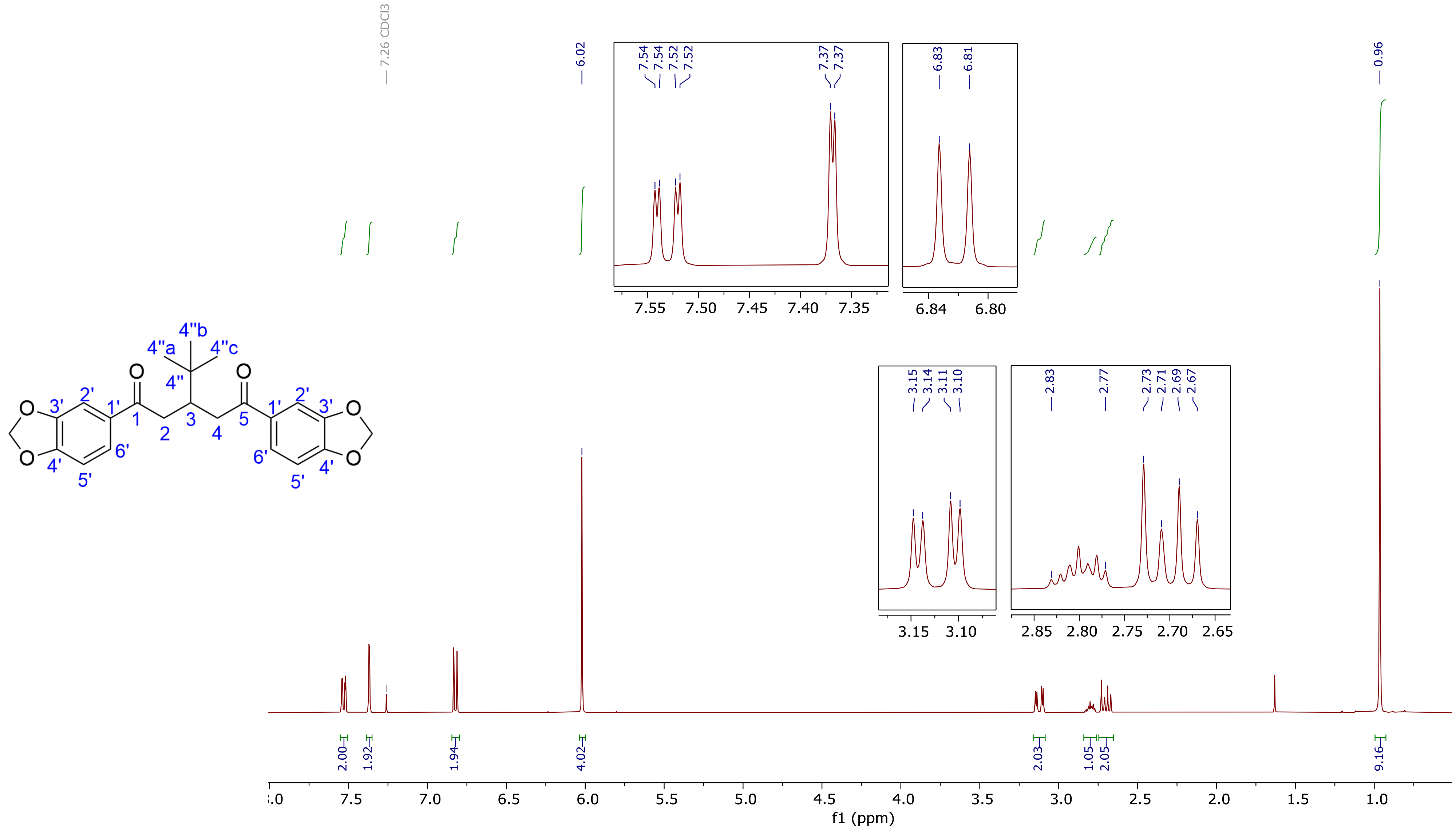

Figure S19. ^{1}H NMR spectrum (400 MHz) of compound **12** in $CDCl_3$.

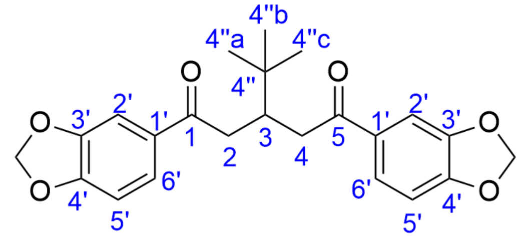

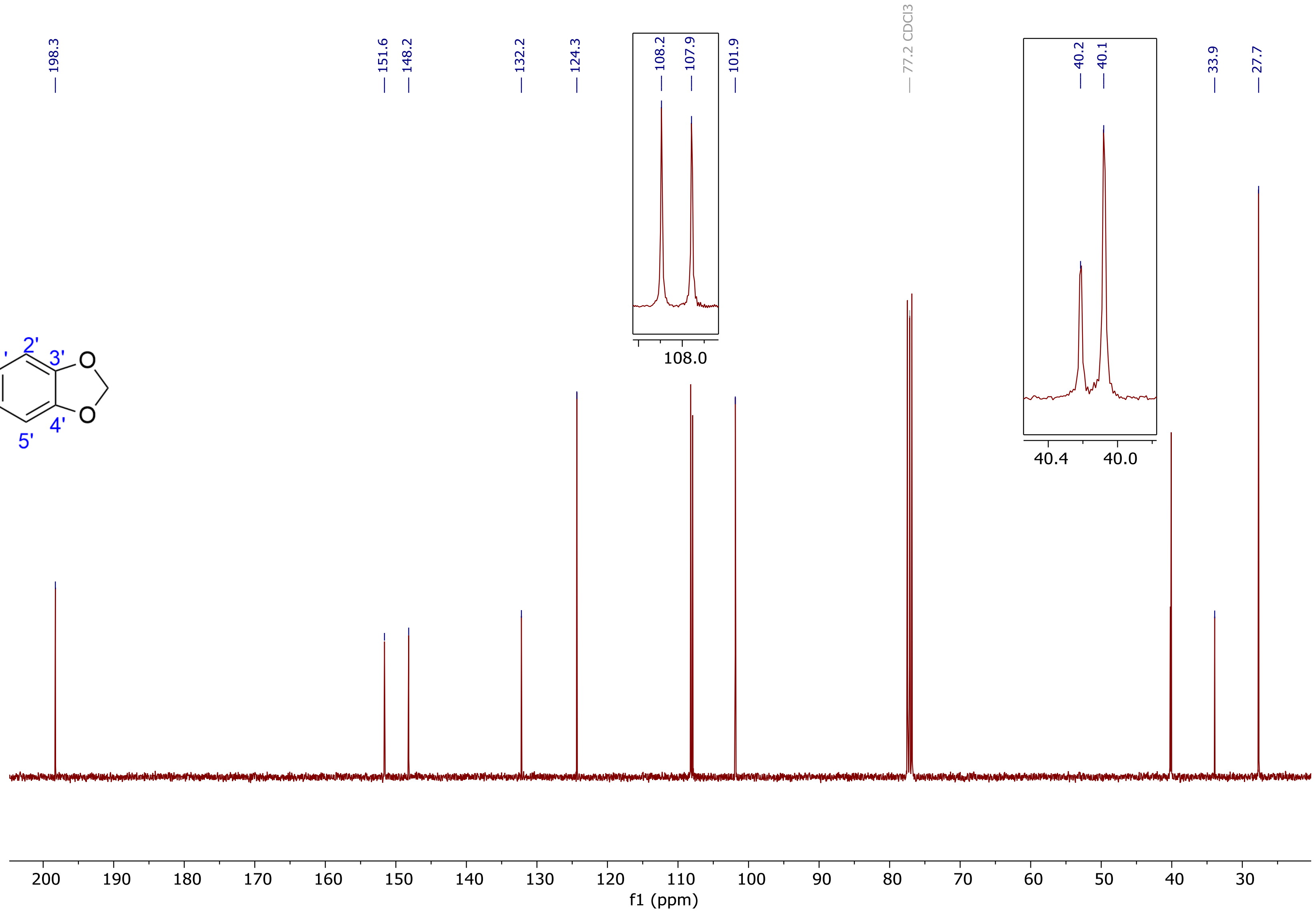

Figure S20. ^{13}C NMR spectrum (100 MHz) of compound **12** in $CDCl_3$.

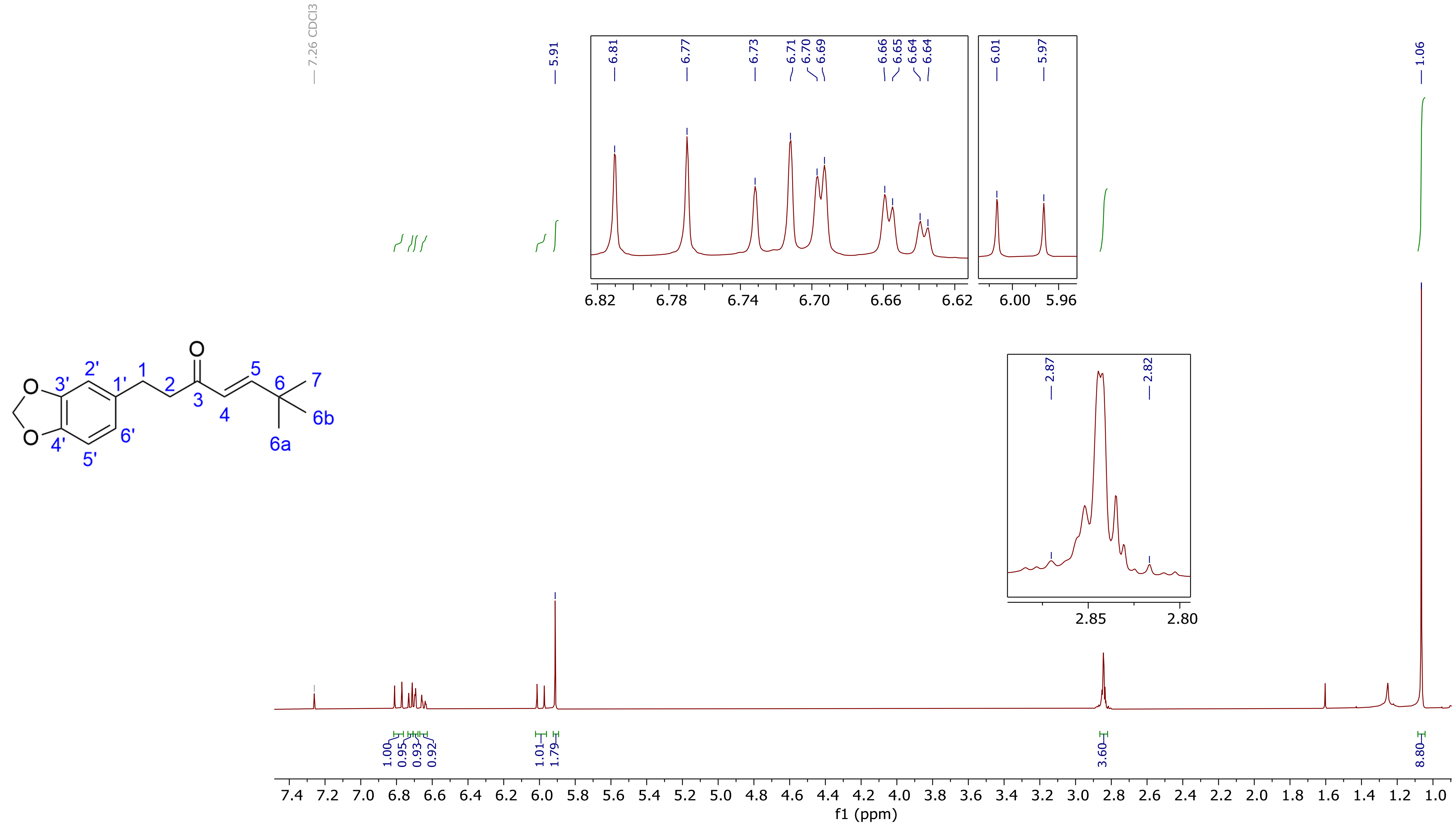

Figure S21. ^{1}H NMR spectrum (400 MHz) of compound **14** in $CDCl_3$.

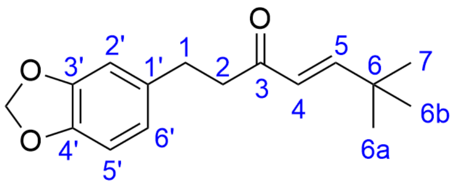

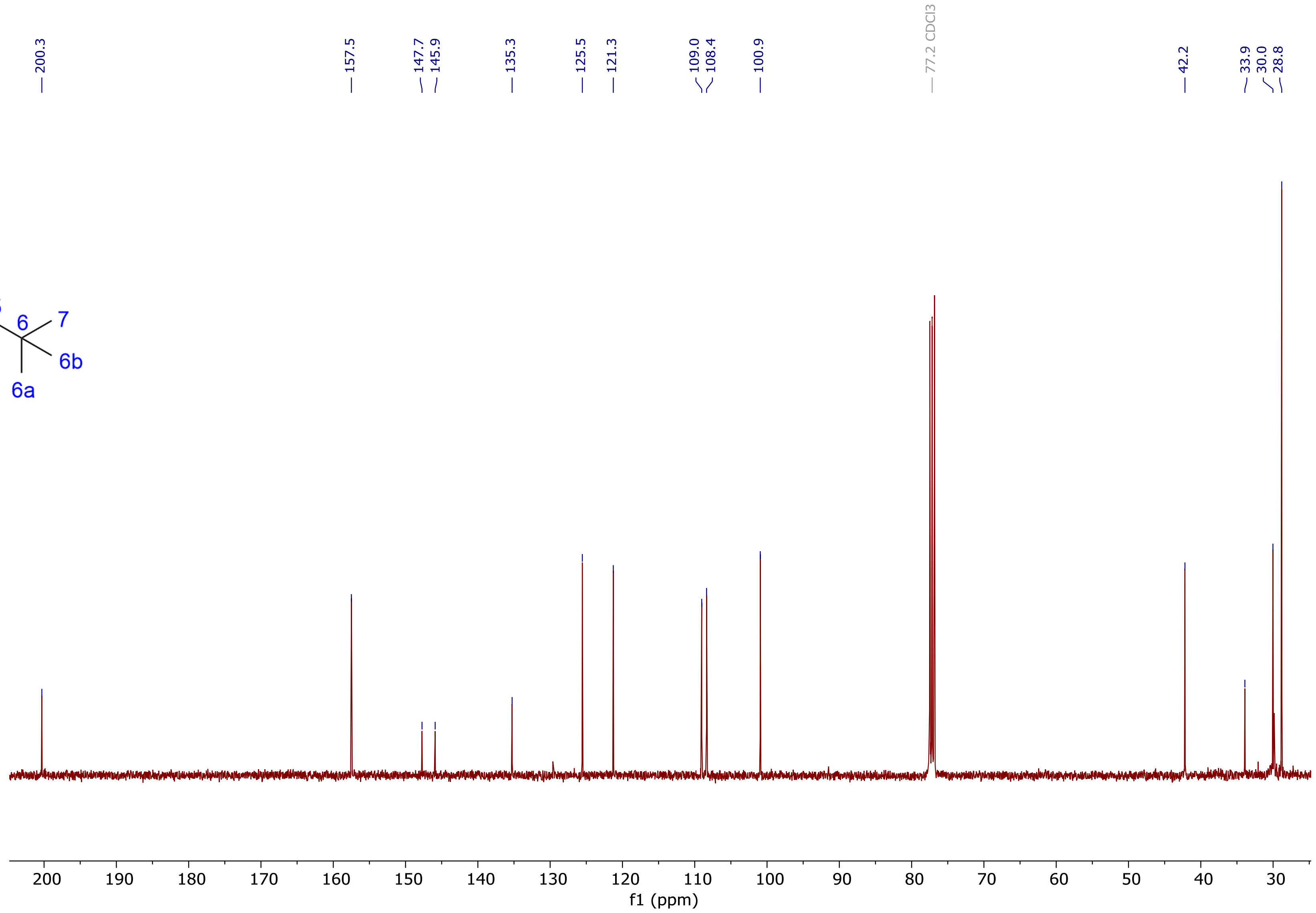

Figure S22. ^{13}C NMR spectrum (100 MHz) of compound **14** in $CDCl_3$.

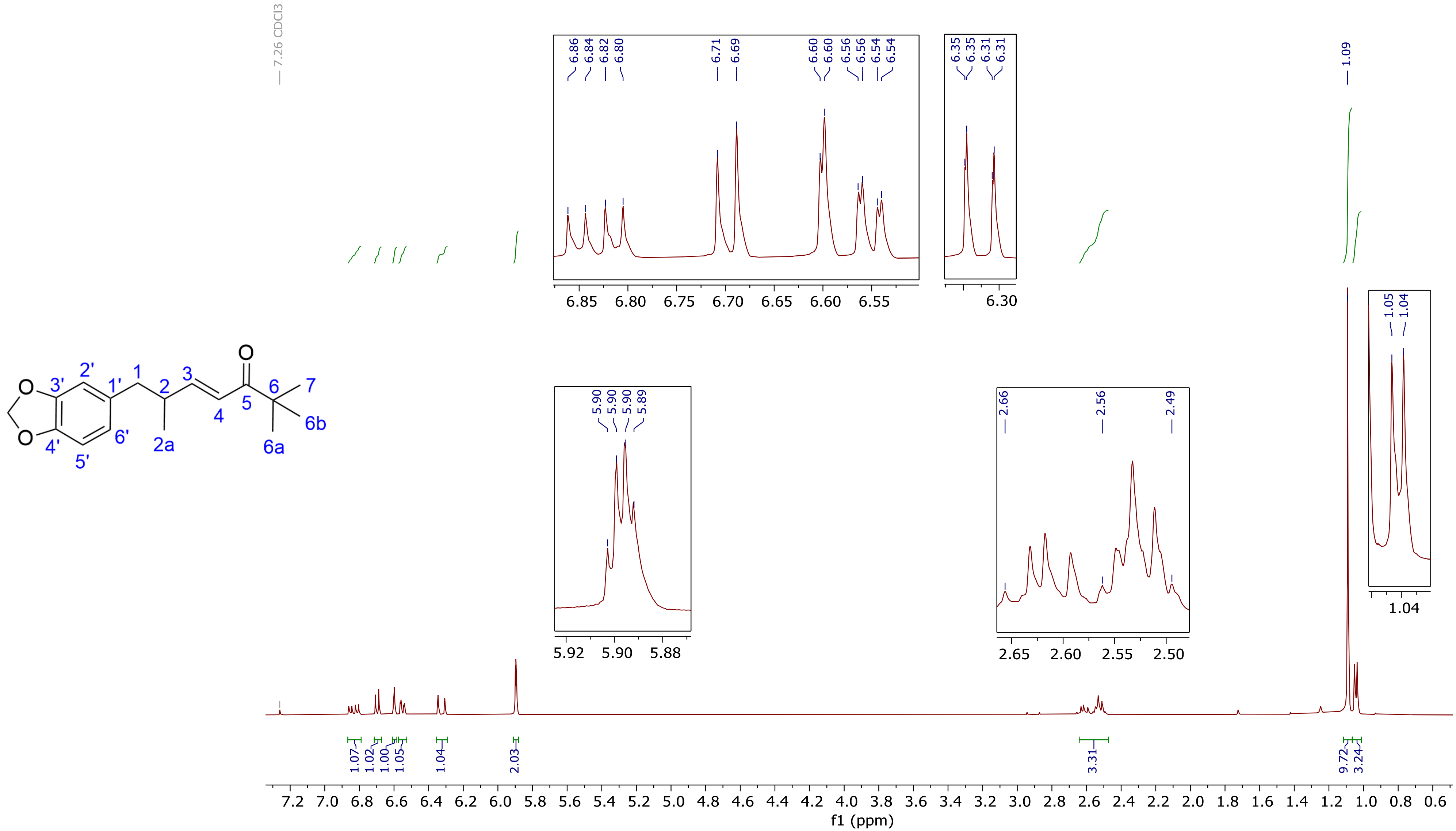

Figure S23. ^{1}H NMR spectrum (400 MHz) of compound **16** in $CDCl_3$.

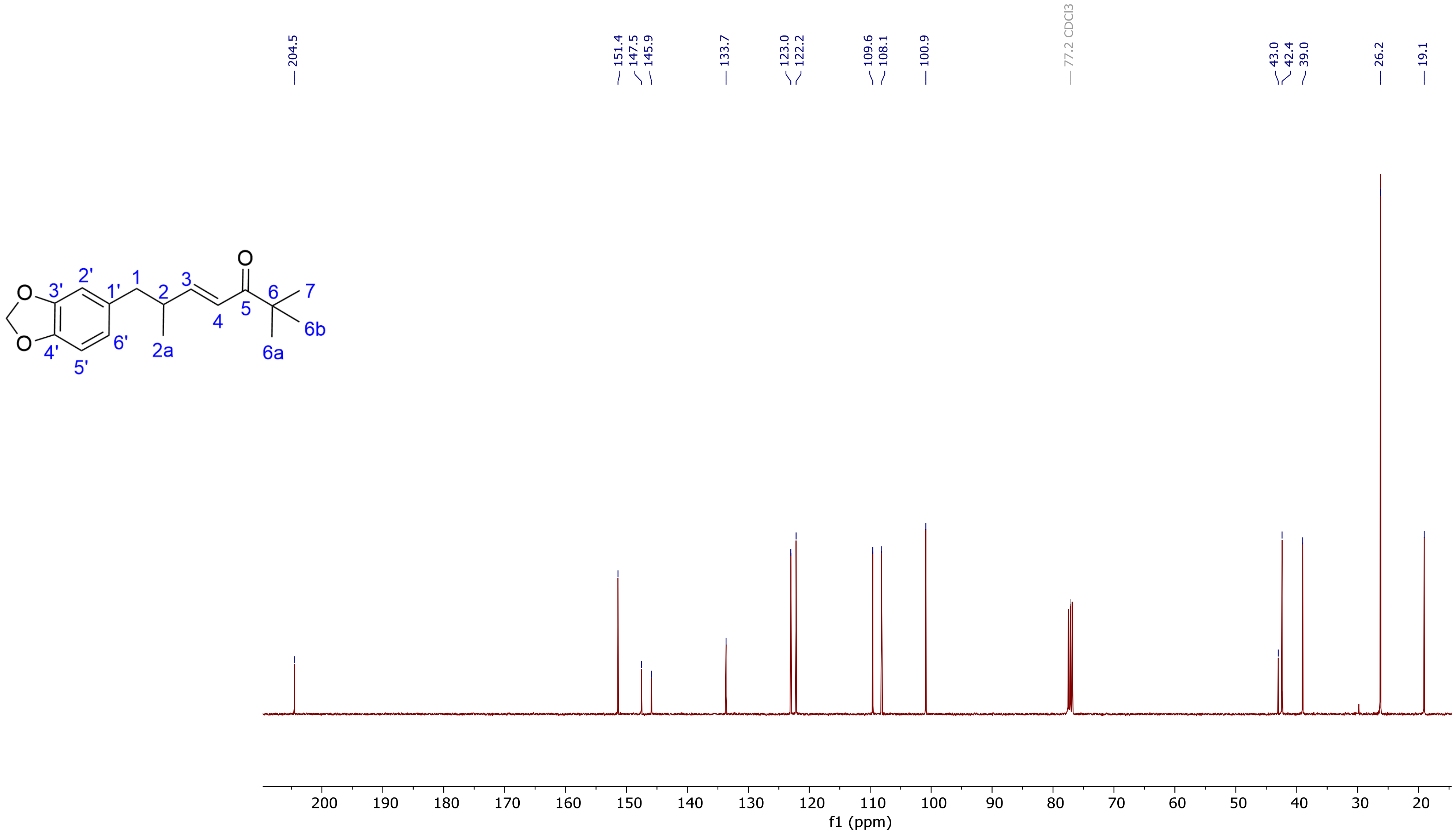

Figure S24. ^{13}C NMR spectrum (100 MHz) of compound **16** in $CDCl_3$.

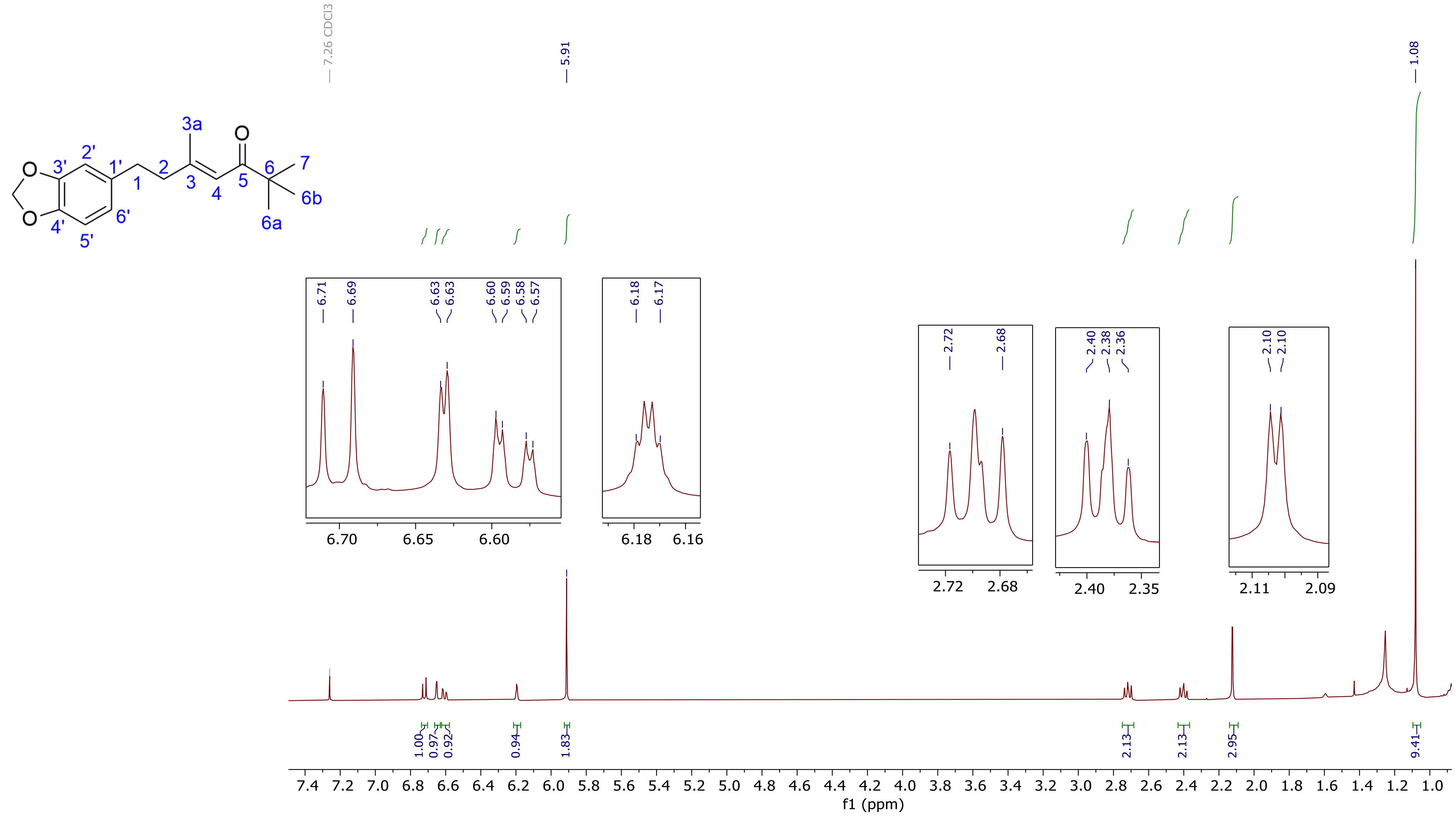

Figure S25. ^{1}H NMR spectrum (400 MHz) of compound **17** in $CDCl_3$.

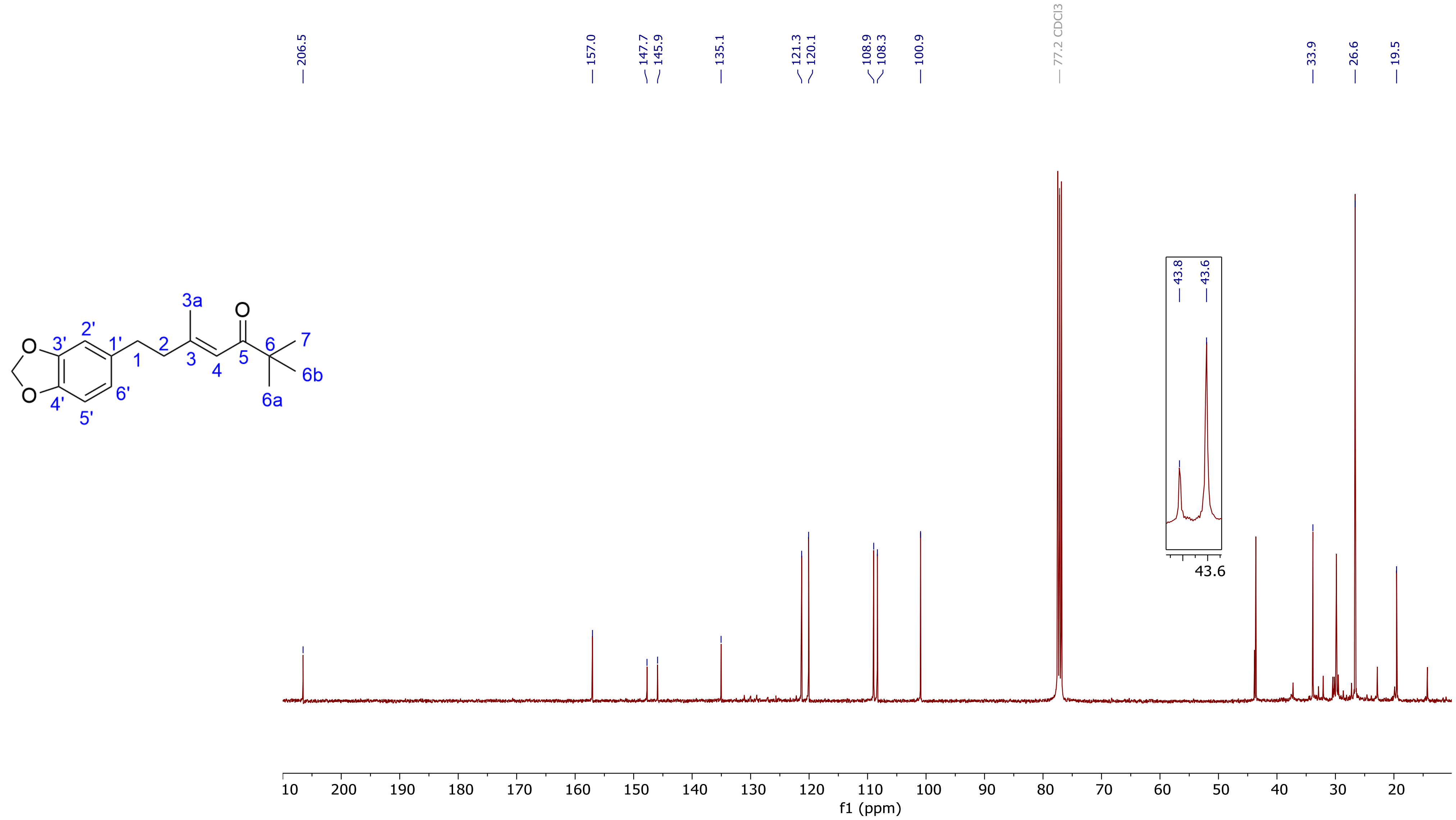

Figure S26. ^{13}C NMR spectrum (100 MHz) of compound **17** in $CDCl_3$.

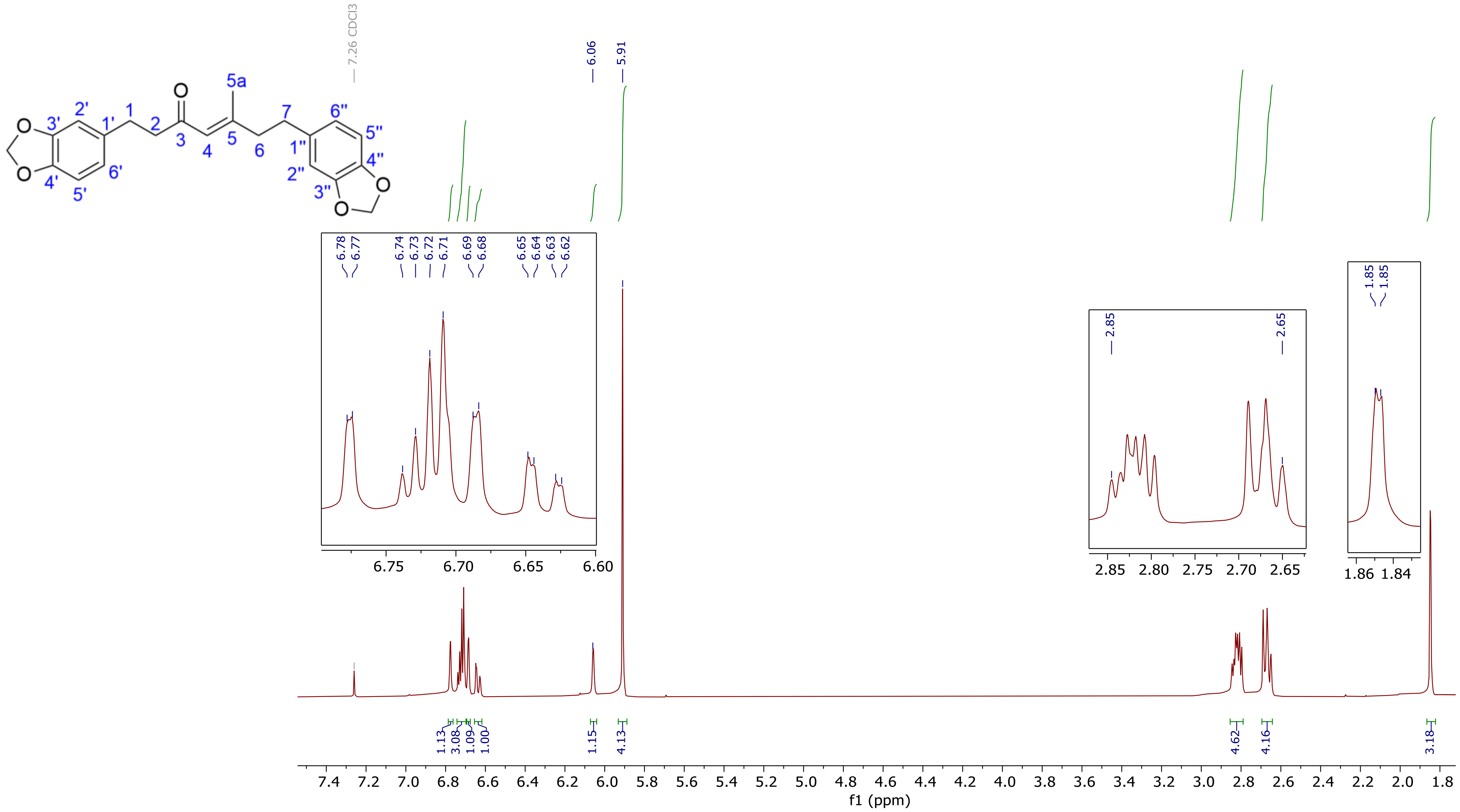

Figure S27. ^{1}H NMR spectrum (400 MHz) of compound **18** in $CDCl_3$.

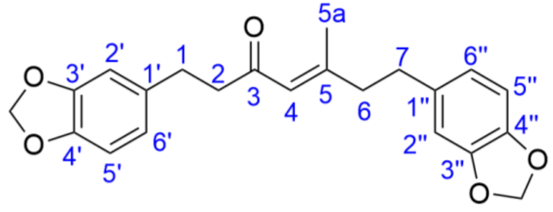

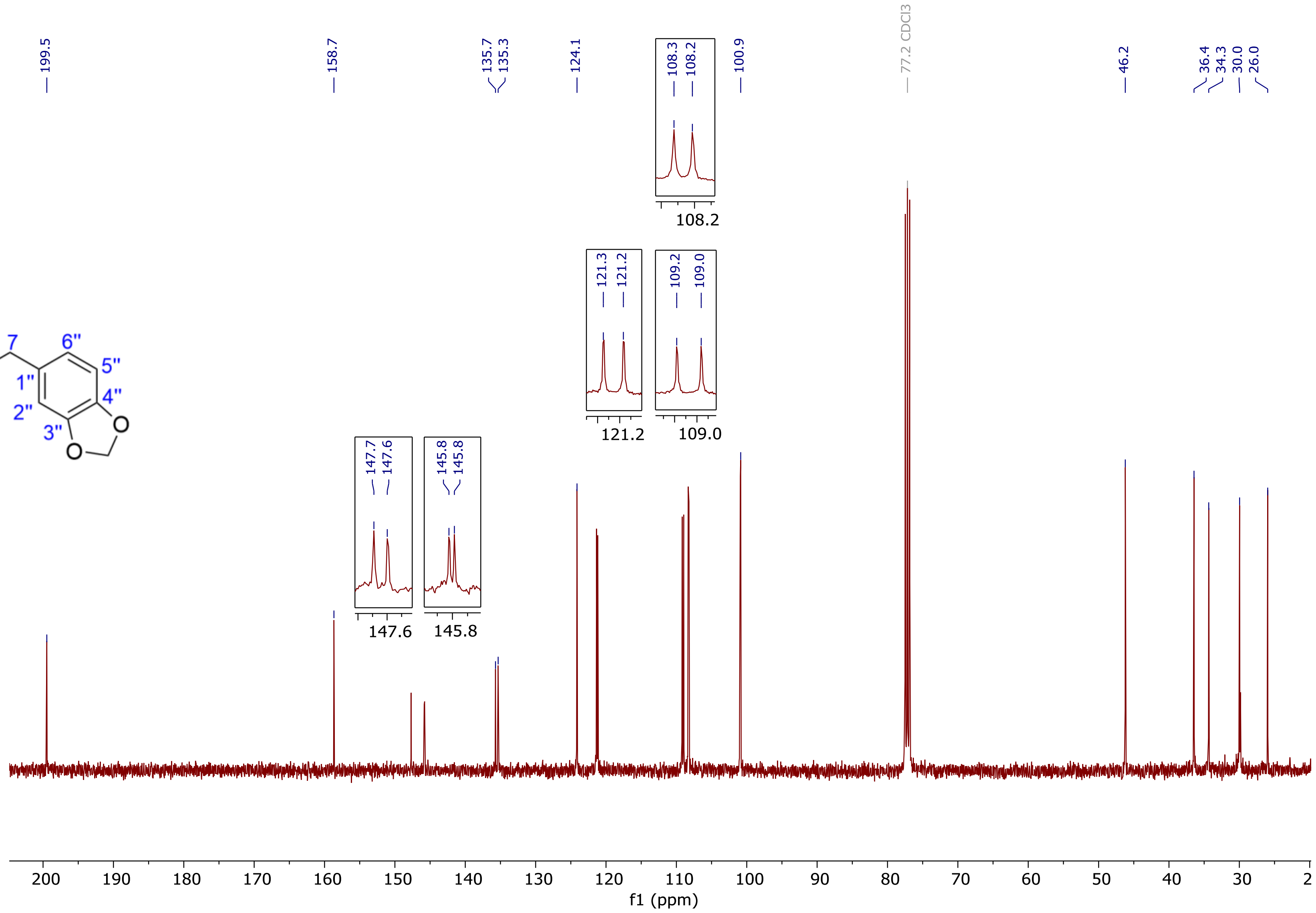

Figure S28. ^{13}C NMR spectrum (100 MHz) of compound **18** in $CDCl_3$.

2. High resolution mass spectra

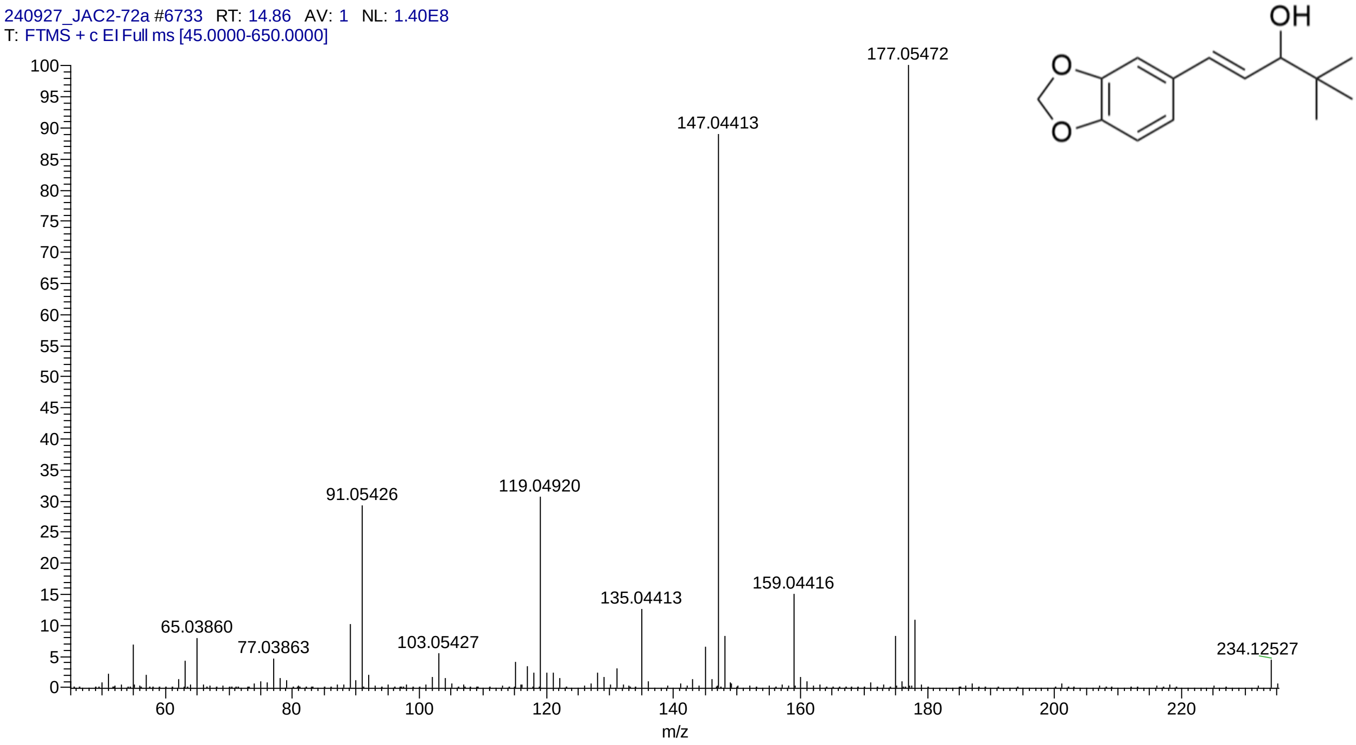

Figure S29. High resolution mass spectrum of compound **1**.

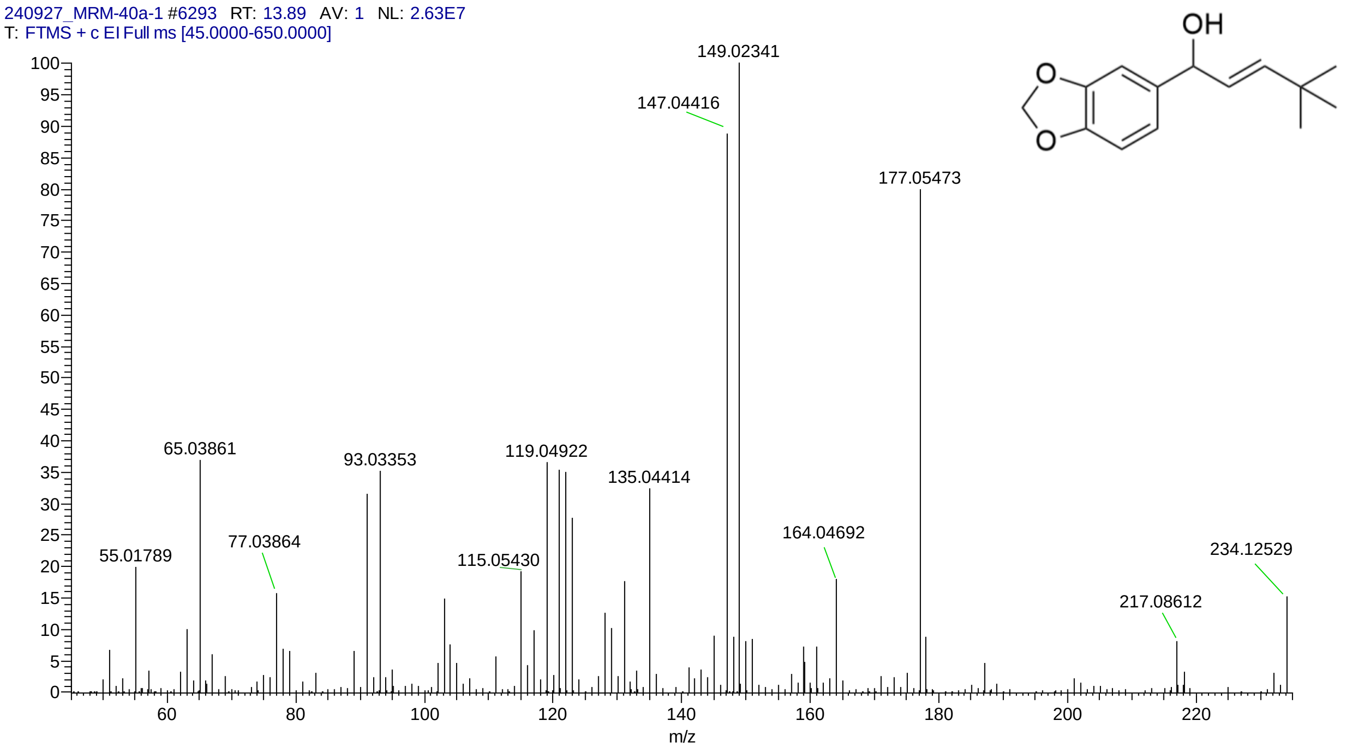

Figure S30. High resolution mass spectrum of compound **2**.

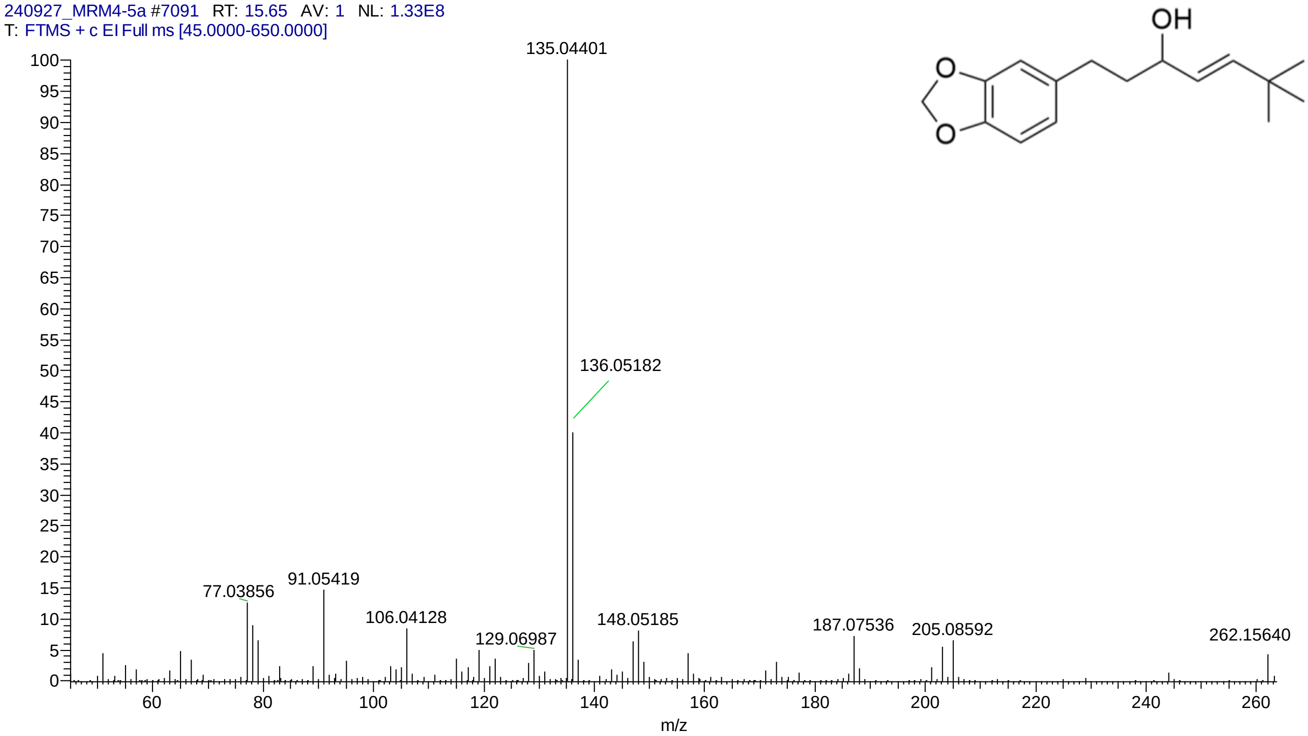

Figure S31. High resolution mass spectrum of compound **3**.

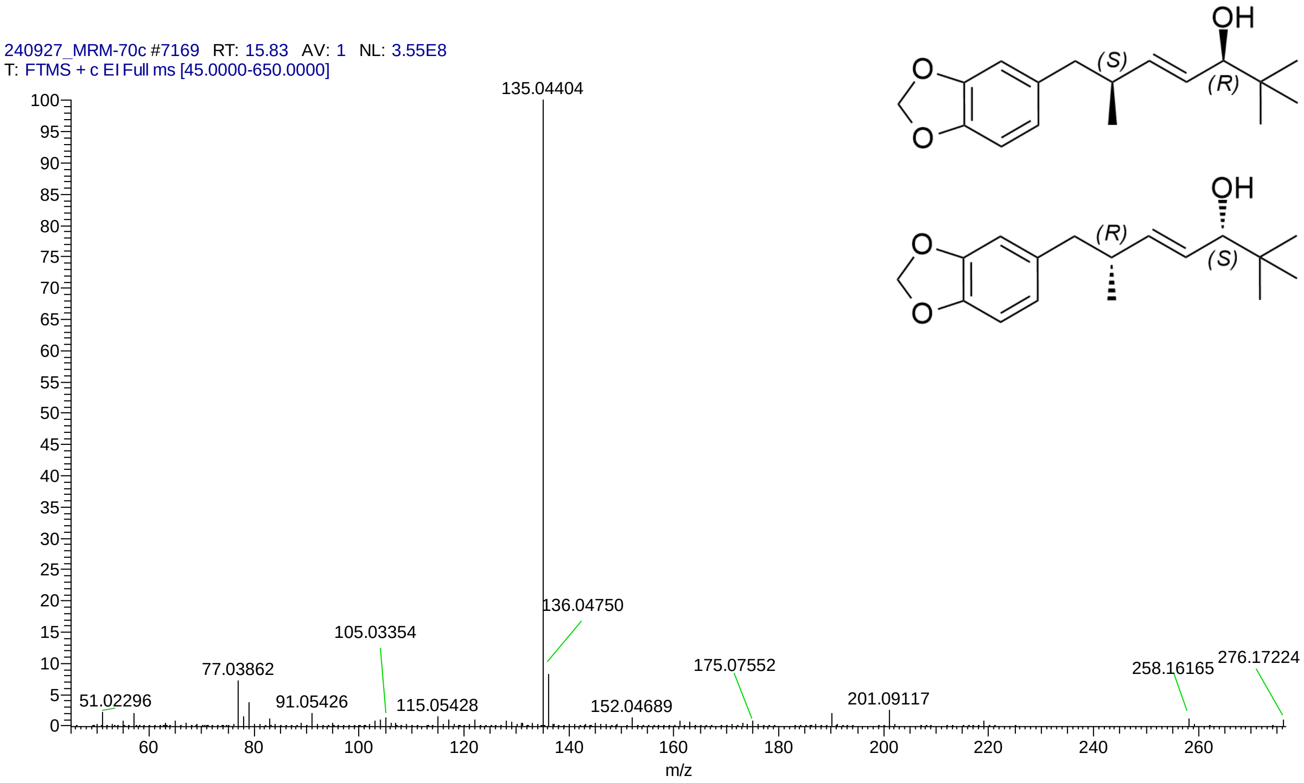

Figure S32. High resolution mass spectrum of compound (2*S**,5*R**)-**4**.

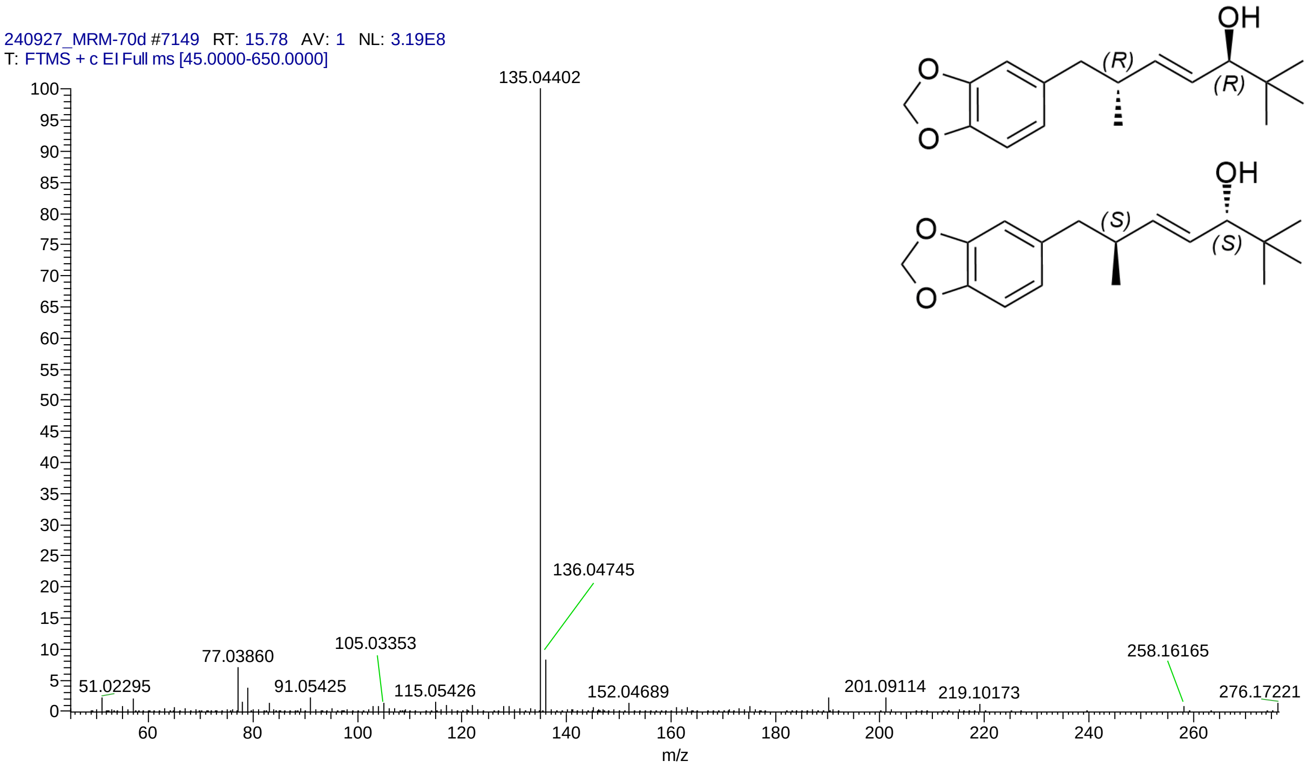

Figure S33. High resolution mass spectrum of compound (2*R**,5*R**)-**4**.

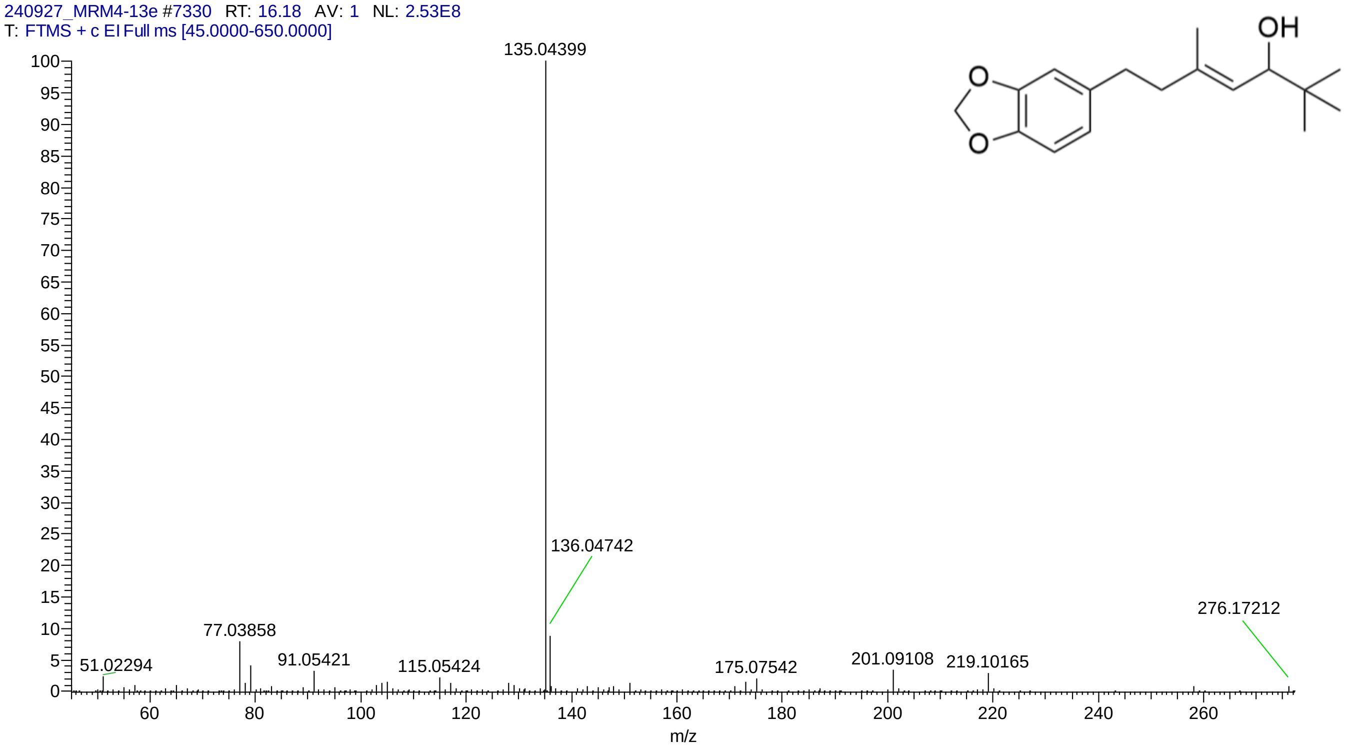

Figure S34. High resolution mass spectrum of compound **5**.

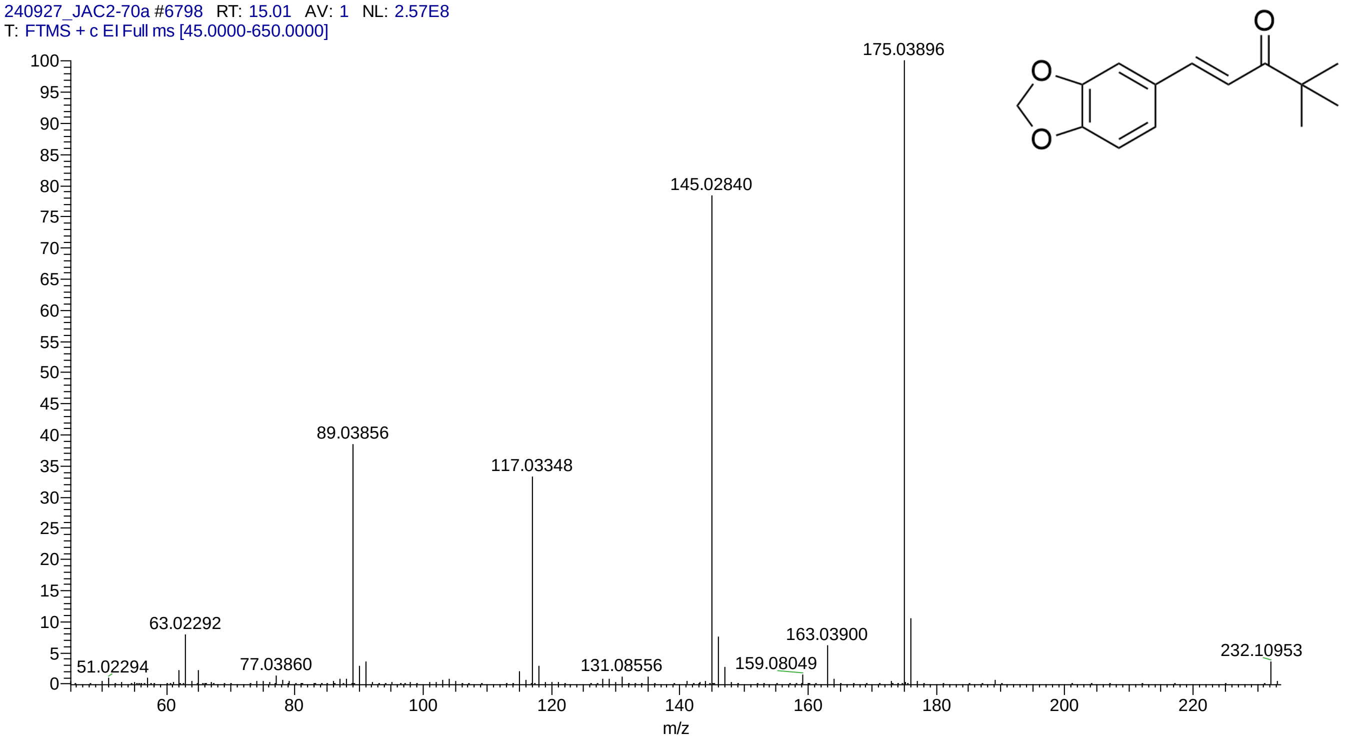

Figure S35. High resolution mass spectrum of compound **8**.

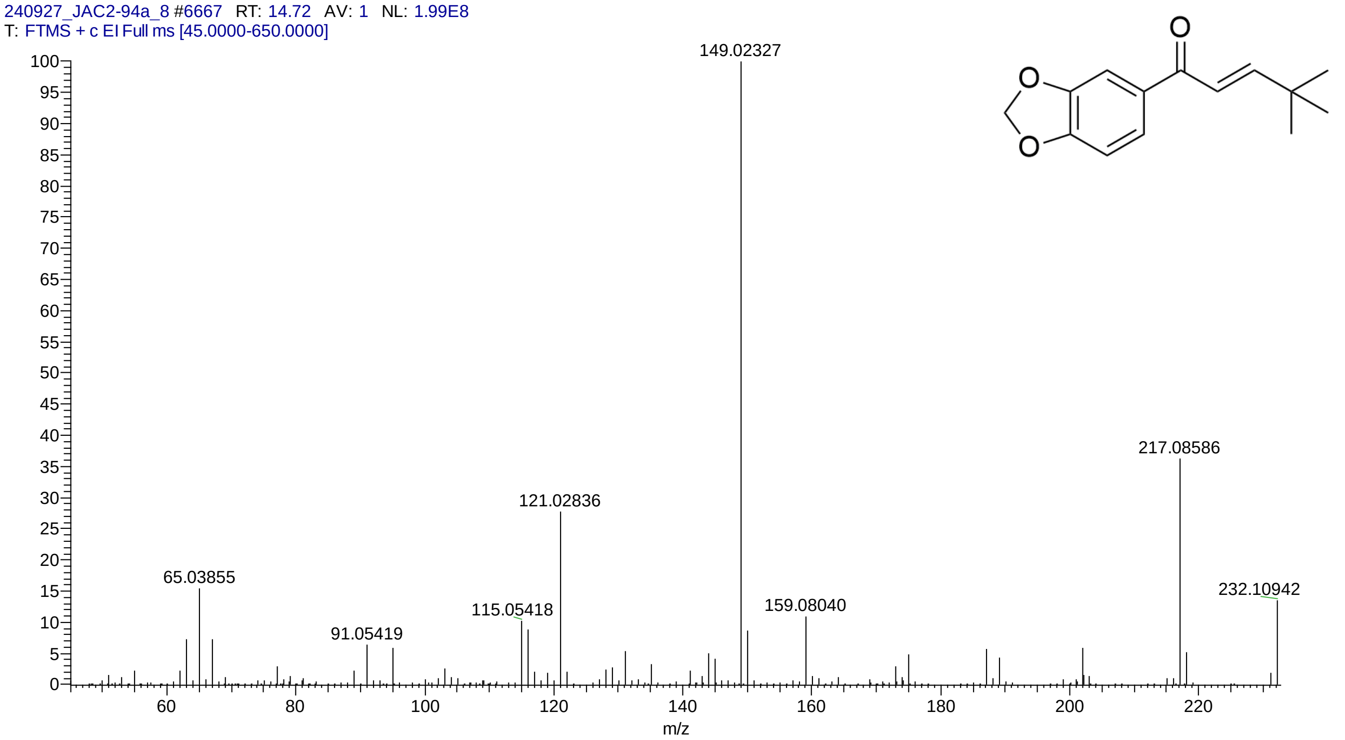

Figure S36. High resolution mass spectrum of compound **11**.

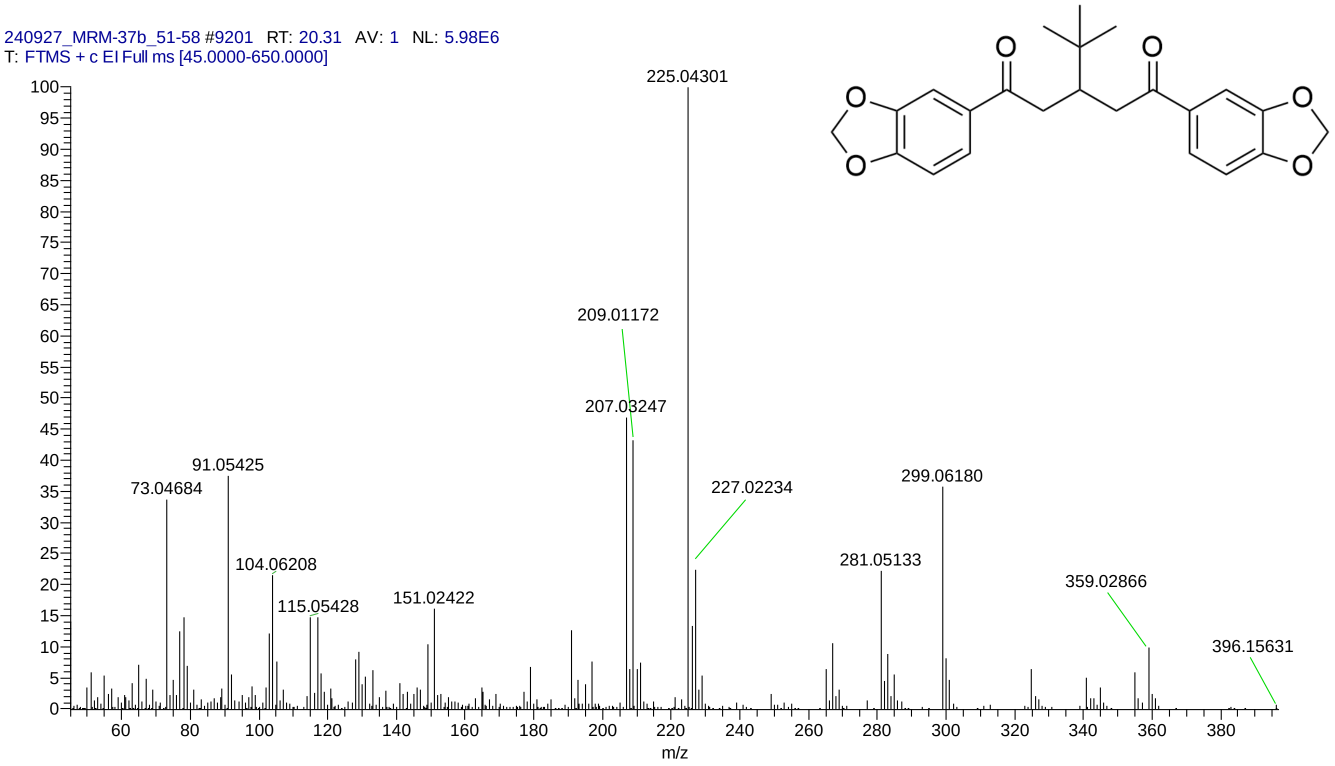

Figure S37. High resolution mass spectrum of compound **12**.

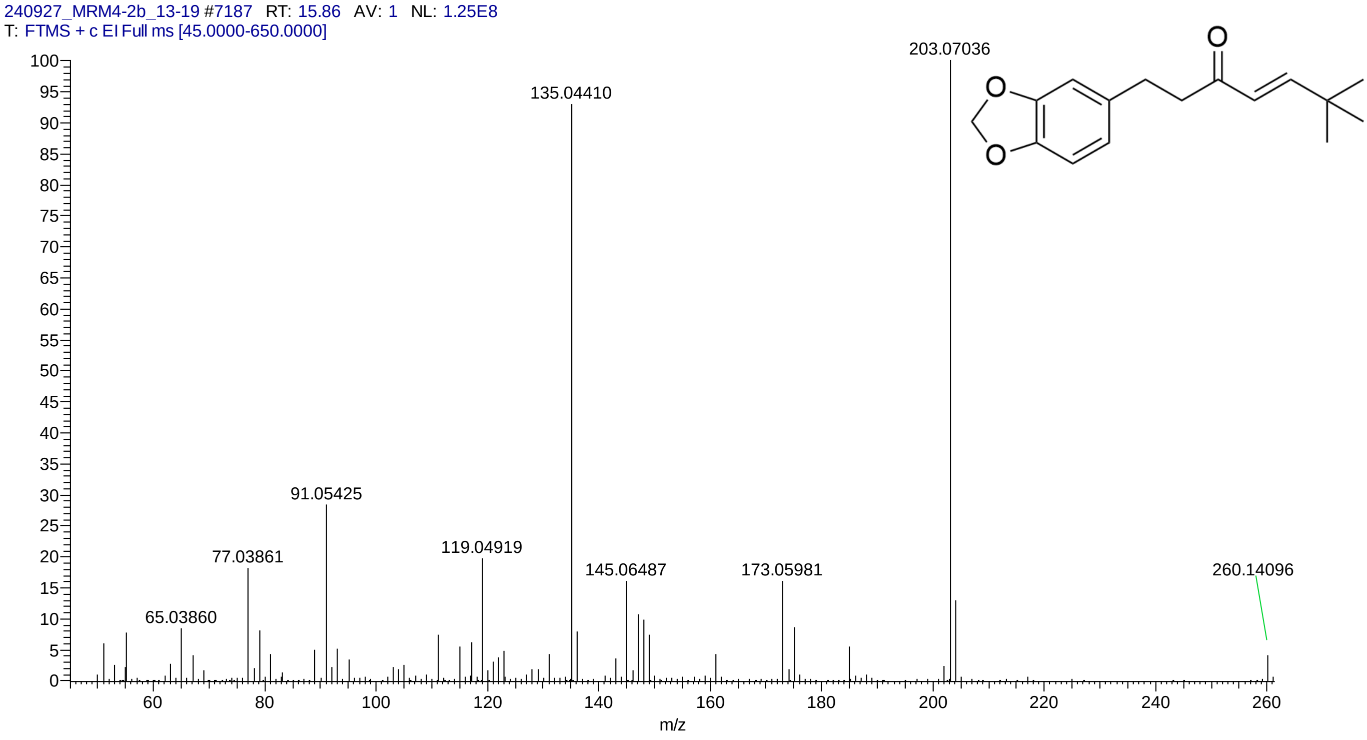

Figure S38. High resolution mass spectrum of compound **14**.

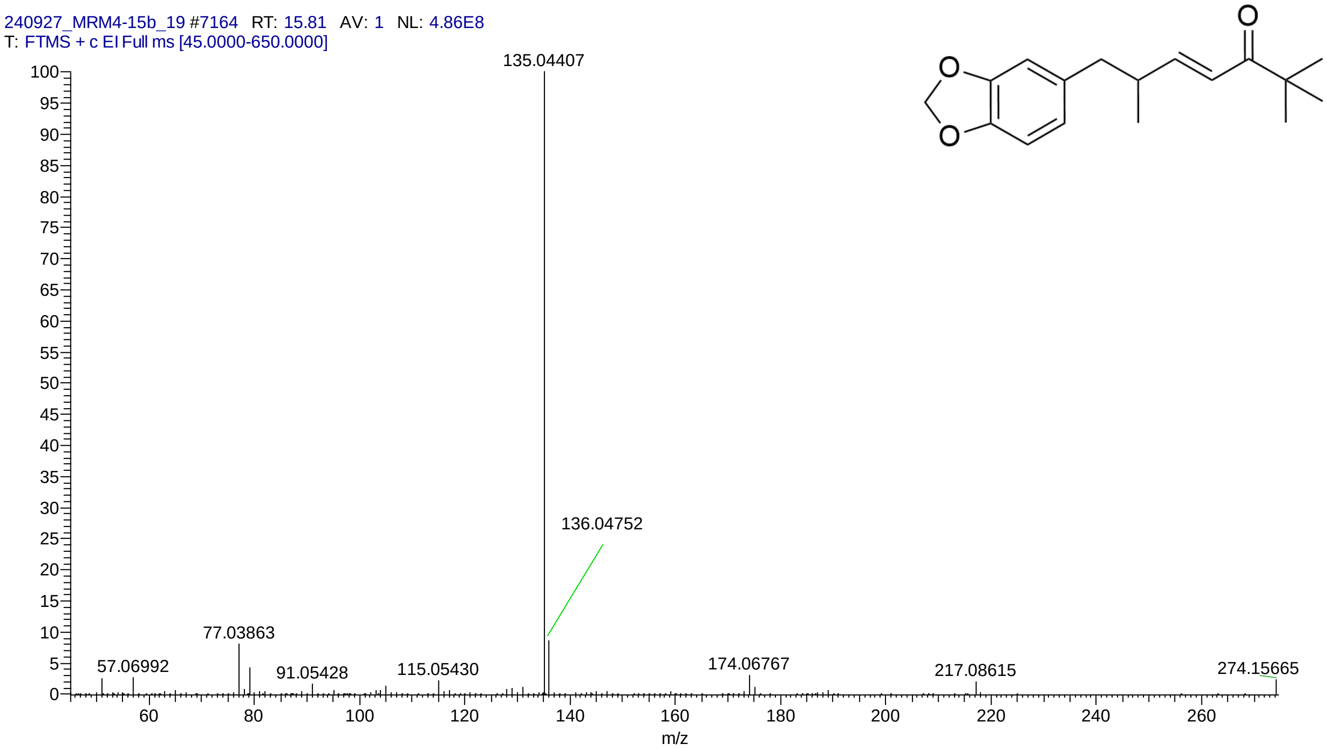

Figure S39. High resolution mass spectrum of compound **16**.

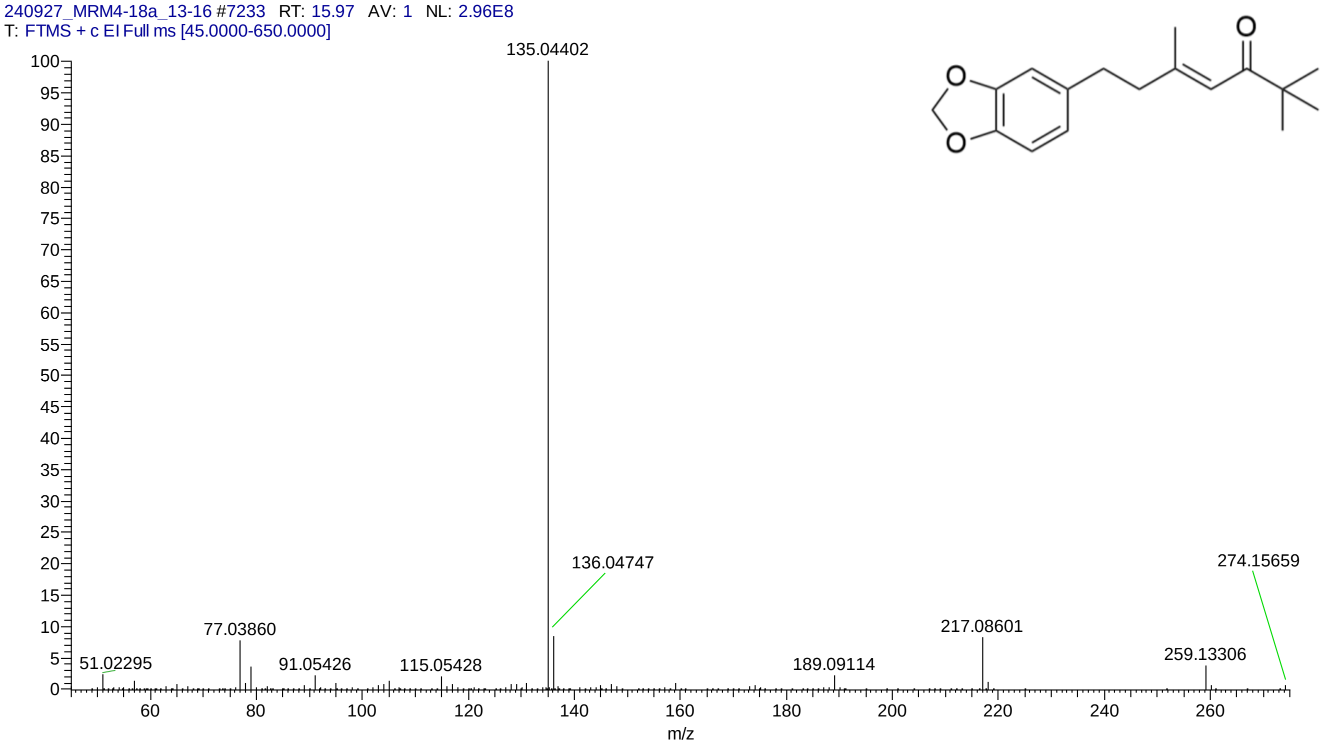

Figure S40. High resolution mass spectrum of compound **17**.

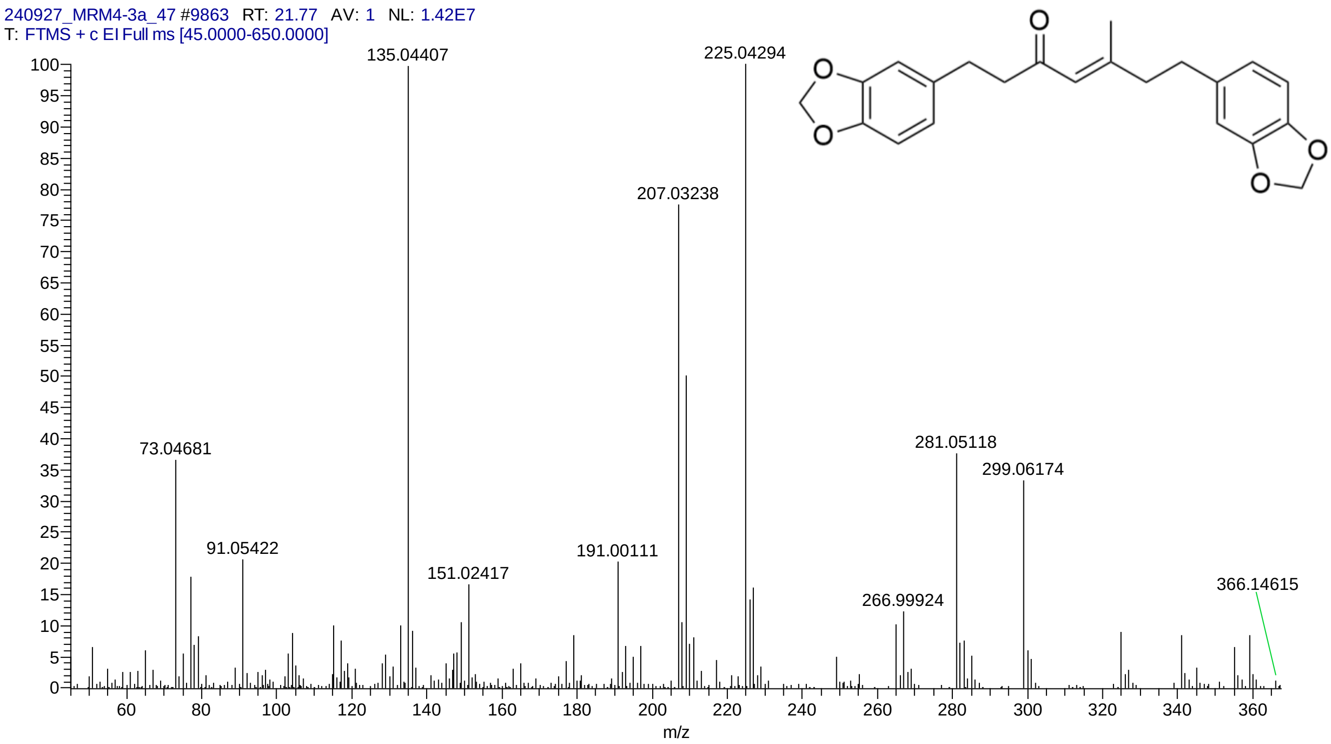

Figure S41. High resolution mass spectrum of compound **18**.

3. HPLC chromatograms

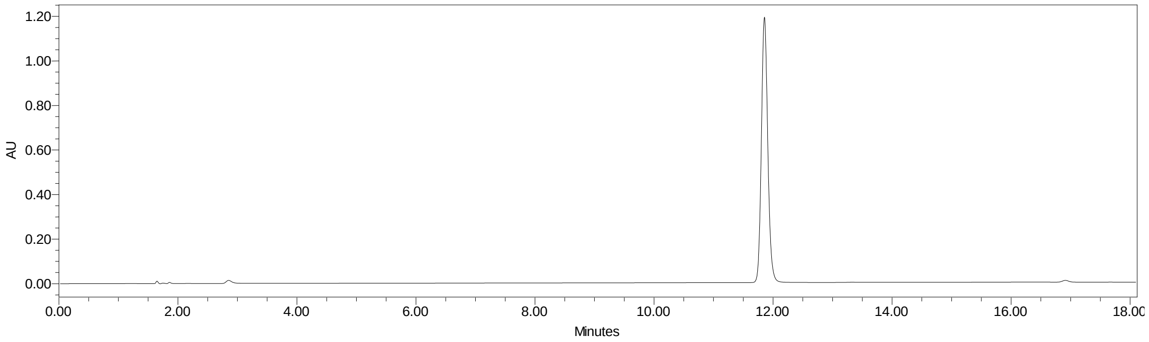

Figure S42. HPLC chromatogram at 254 nm of compound **1** (t_R = 11.9 min). Purity: 99%.

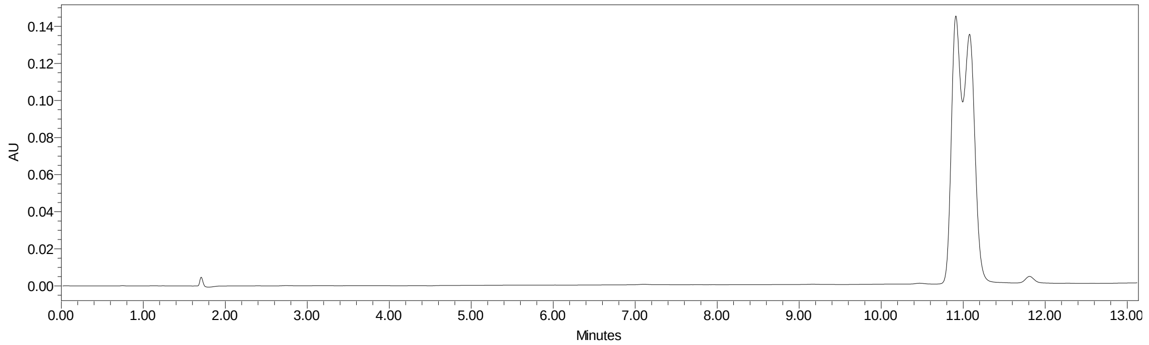

Figure S43. HPLC chromatogram at 254 nm of compound **2** (t_R = 10.9 min). The peak at t_R = 11.1 min corresponds to stiripentol (**1**).

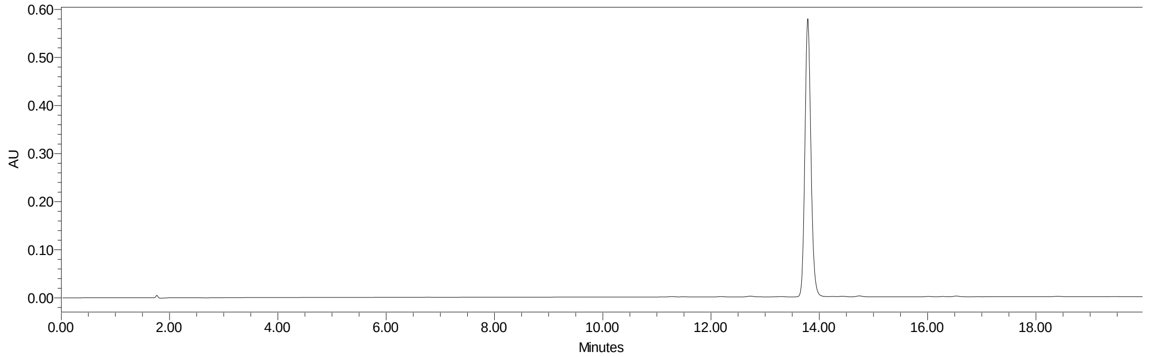

Figure S44. HPLC chromatogram at 285 nm of compound **3** (t_R = 13.8 min). Purity: >99%.

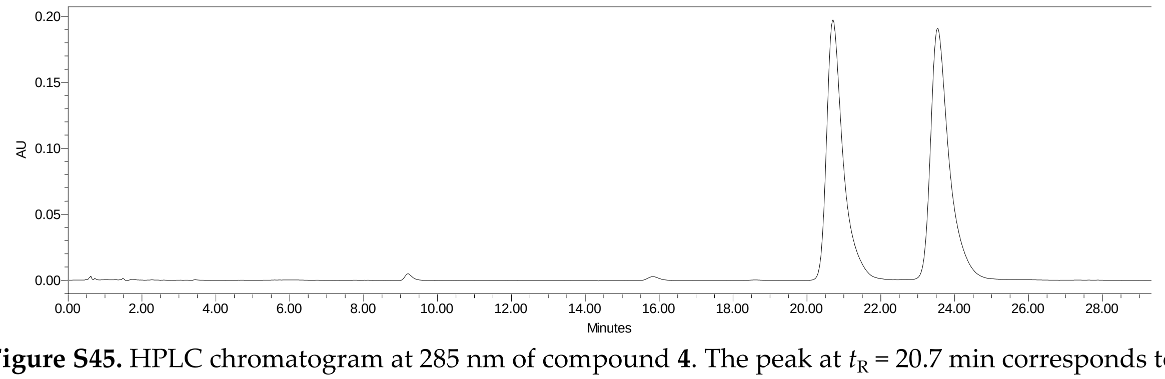

Figure S45. HPLC chromatogram at 285 nm of compound **4**. The peak at t_R = 20.7 min corresponds to diastereomer (*2S*,5R**)-**4**. The peak at t_R = 23.5 min corresponds to diastereomer (*2R*,5R**)-**4**.

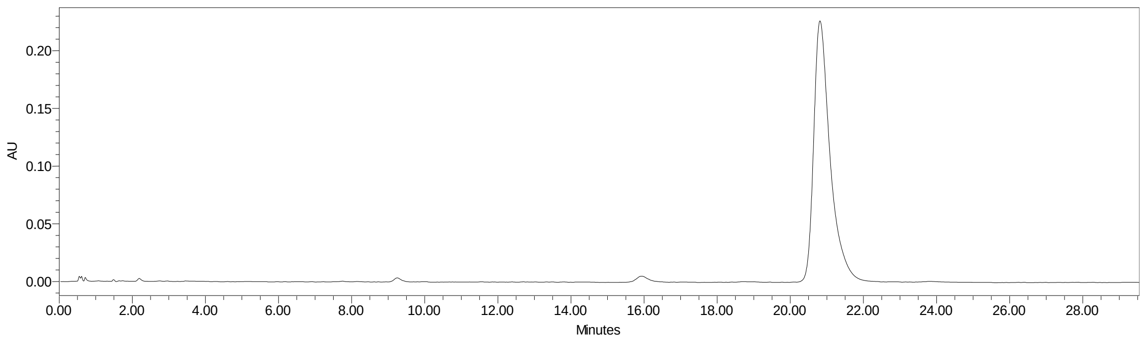

Figure S46. HPLC chromatogram at 285 nm of compound (*2S*,5R**)-**4**. Purity: 98%.

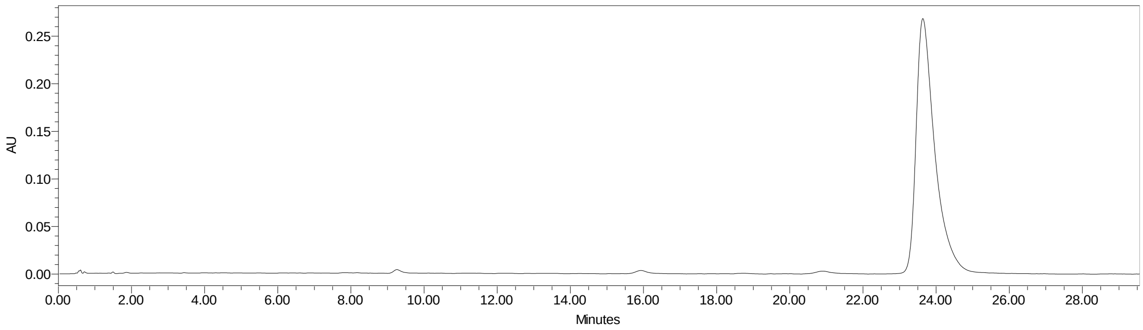

Figure S47. HPLC chromatogram at 285 nm of compound (*2R*,5R**)-**4**. Purity: 99%.

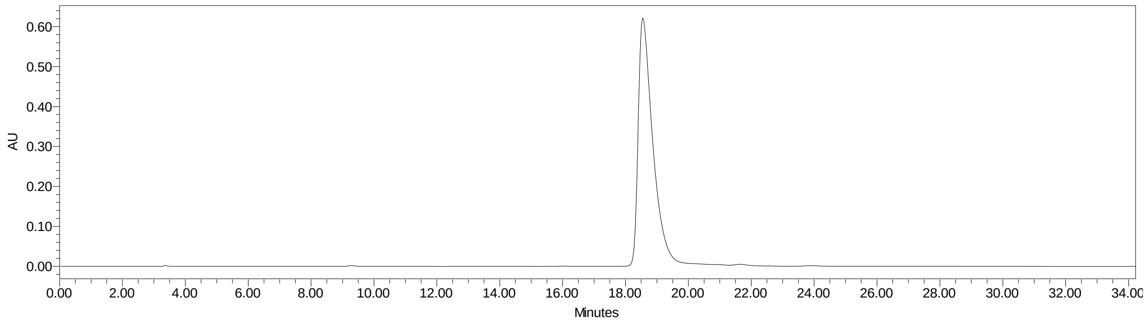

Figure S48. HPLC chromatogram at 285 nm of compound **5** (t_R = 18.6 min). Purity: >99%.

4. Chiral HPLC chromatograms.

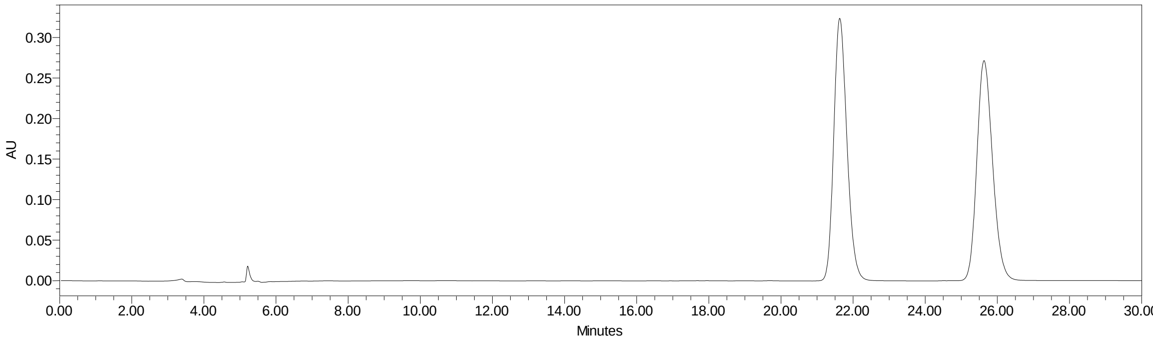

Figure S49. Chiral HPLC chromatogram at 254 nm of compound (±)-**1**. The peak at t_R = 21.6 min corresponds to enantiomer (–)-(*S*)-**1**. The peak at t_R = 25.6 min corresponds to enantiomer (+)-(*R*)-**1**.

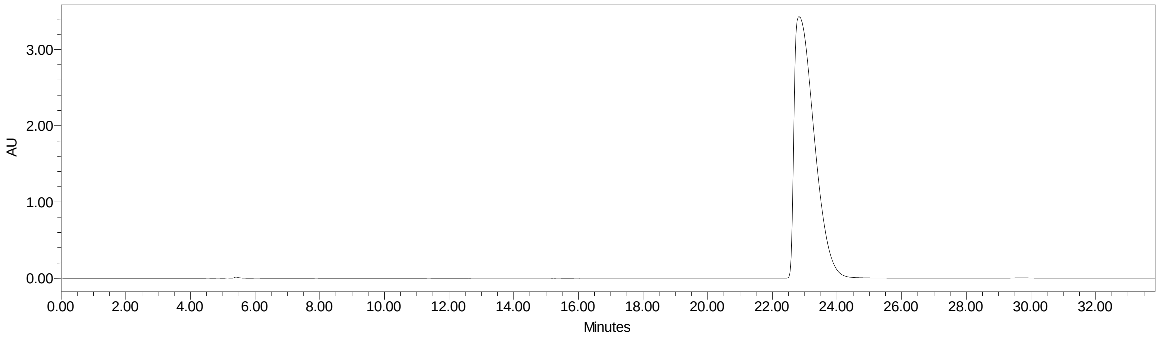

Figure S50. Chiral HPLC chromatogram at 254 nm of compound (–)-(*S*)-**1**. Purity: >99%.

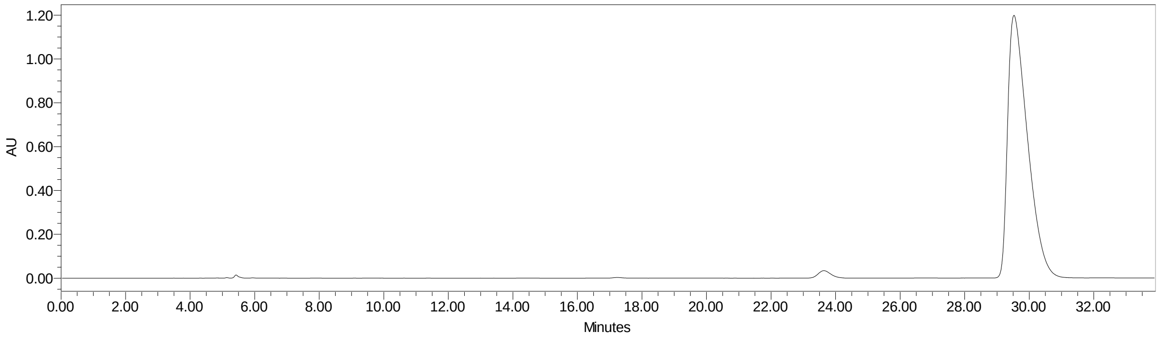

Figure S51. Chiral HPLC chromatogram at 254 nm of compound (+)-(*R*)-**1.** Purity: 98%.

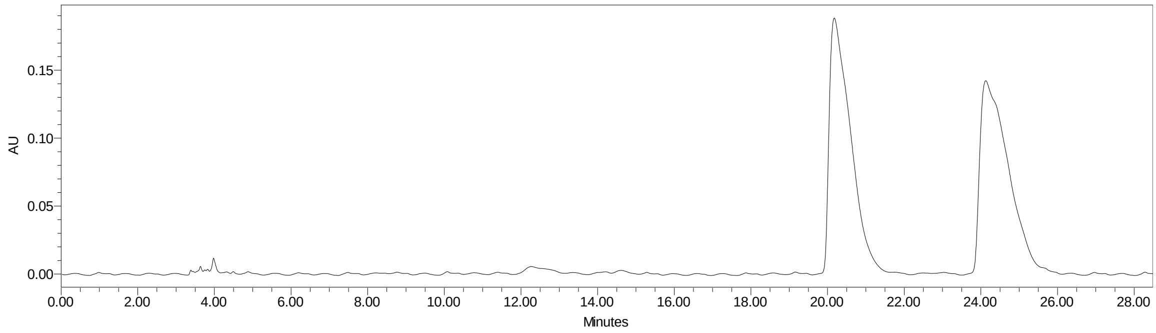

Figure S52. Chiral HPLC chromatogram at 285 nm of compound (±)-**3**. The peak at t_R = 20.2 min corresponds to enantiomer (–)-(*S*)-**3**. The peak at t_R = 24.1 min corresponds to enantiomer (+)-(*R*)-**3**.

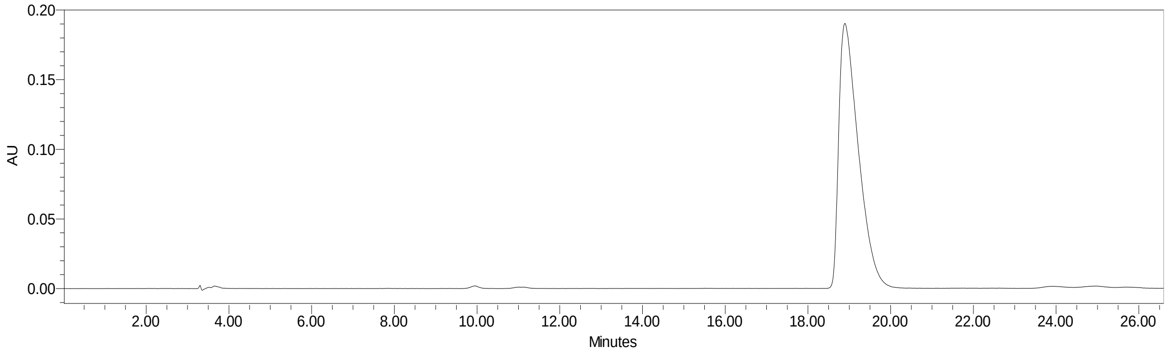

Figure S53. Chiral HPLC chromatogram at 285 nm of compound (**–**)-(*S*)-**3**. Purity: >99%.

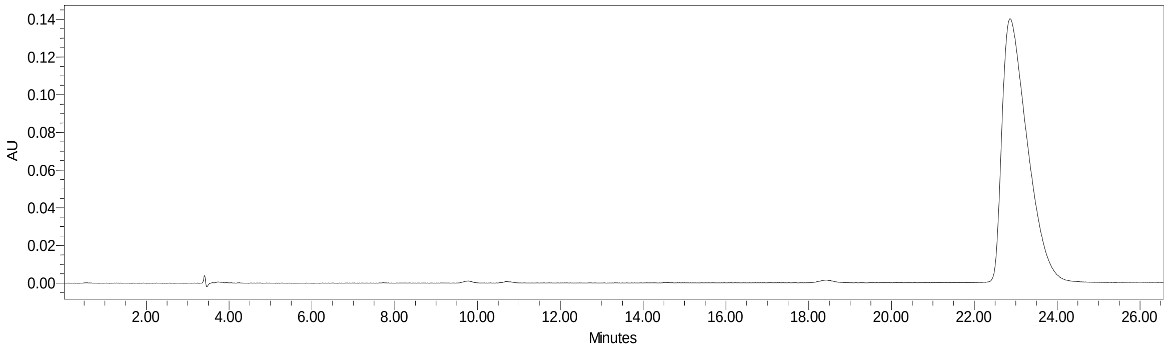

Figure S54. Chiral HPLC chromatogram at 285 nm of compound (+)-(*R*)-**3**. Purity: >99%.

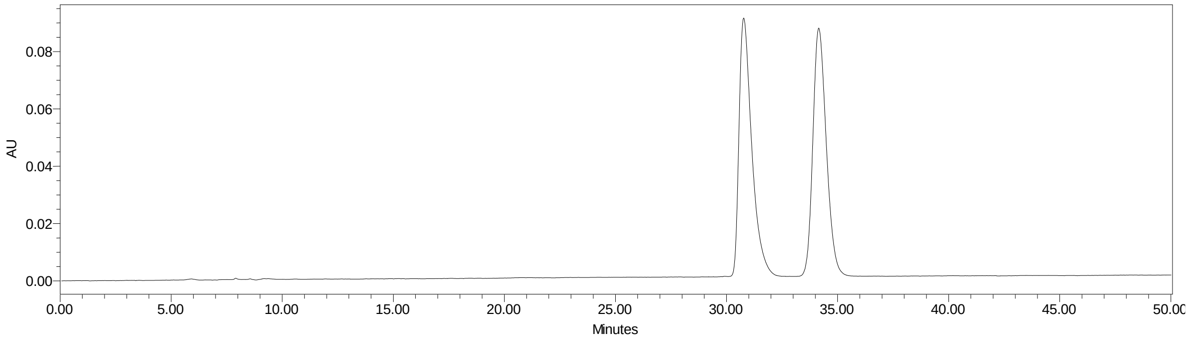

Figure S55. Chiral HPLC chromatogram at 285 nm of compound (±)-(2*S**,5*R**)-**4**. The peak at t_R = 30.8 min corresponds to enantiomer (**–**)-(2*R*,5*S*)-**4**. The peak at t_R = 34.2 min corresponds to enantiomer (+)-(2*S*,5*R*)-**4**.

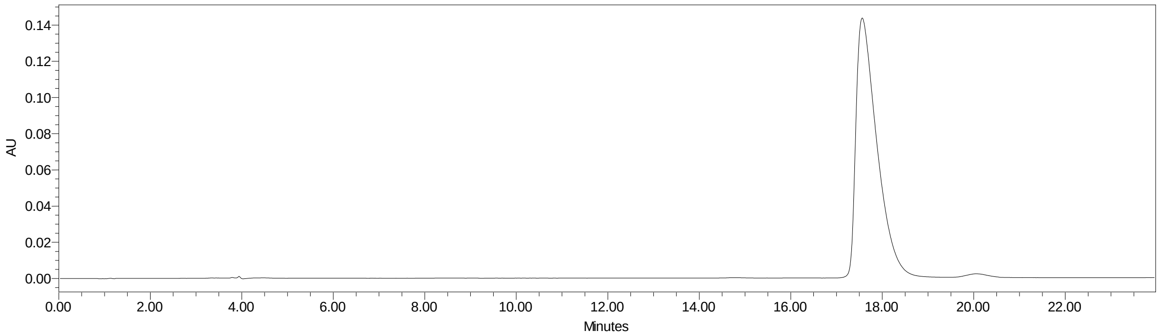

Figure S56. Chiral HPLC chromatogram at 285 nm of compound (**–**)-(2*R*,5*S*)-**4**. Purity: 98%.

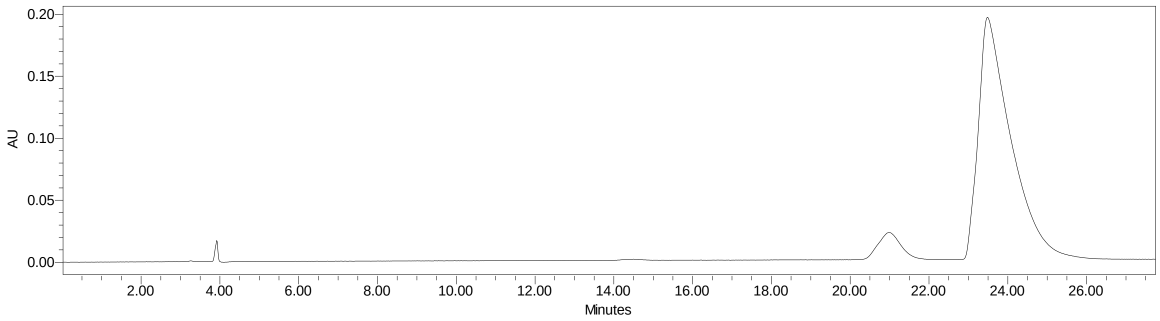

Figure S57. Chiral HPLC chromatogram at 285 nm of compound (+)-(2*S*,5*R*)-**4**. Purity: 95%.

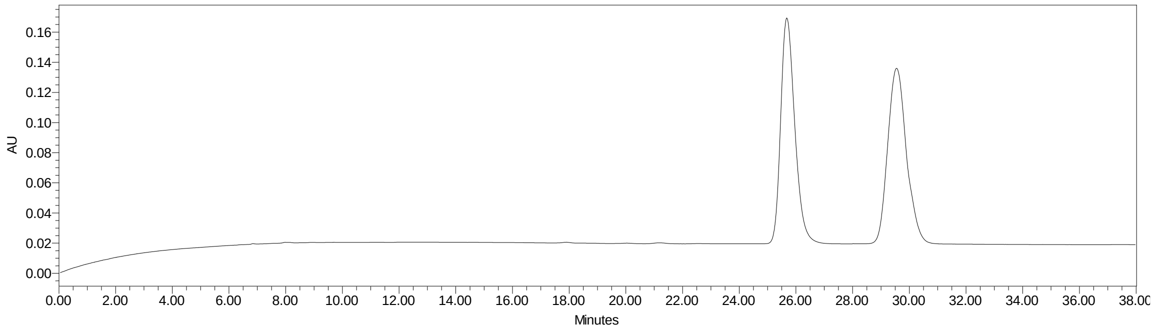

Figure S58. Chiral HPLC chromatogram at 285 nm of compound (±)-(2*R**,5*R**)-**4**. The peak at t_R = 25.7 min corresponds to enantiomer (+)-(2*R*,5*R*)-**4**. The peak at t_R = 29.6 min corresponds to enantiomer **(–)**-(2*S*,5*S*)-**4**.

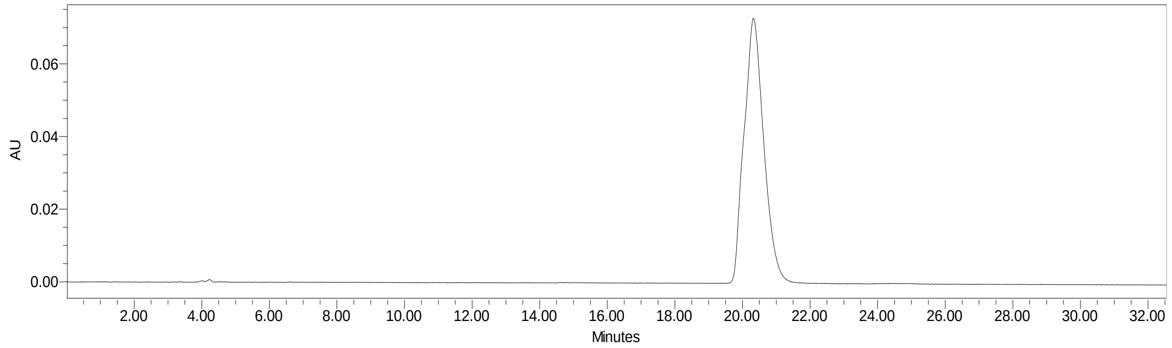

Figure S59. Chiral HPLC chromatogram at 285 nm of compound (+)-(2*R*,5*R*)-**4**. Purity: 99%.

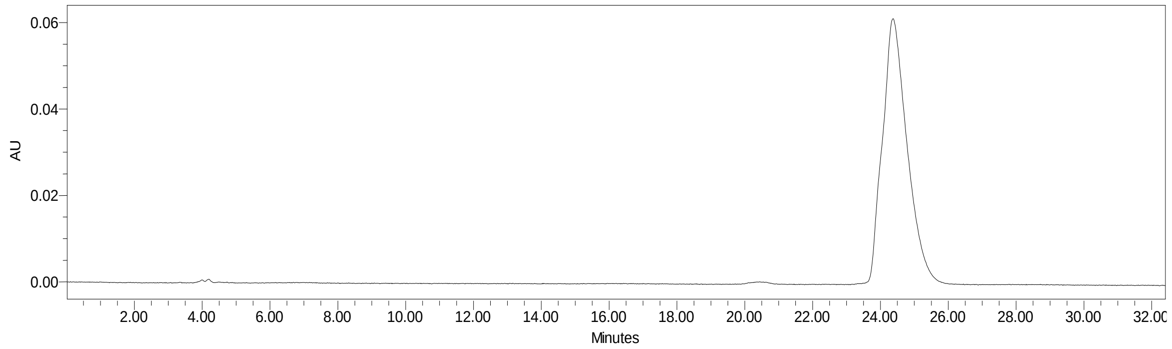

Figure S60. Chiral HPLC chromatogram at 285 nm of compound **(–)**-(2*S*,5*S*)-**4**. Purity: 99%.

5. DFT-optimized lowest energy conformers

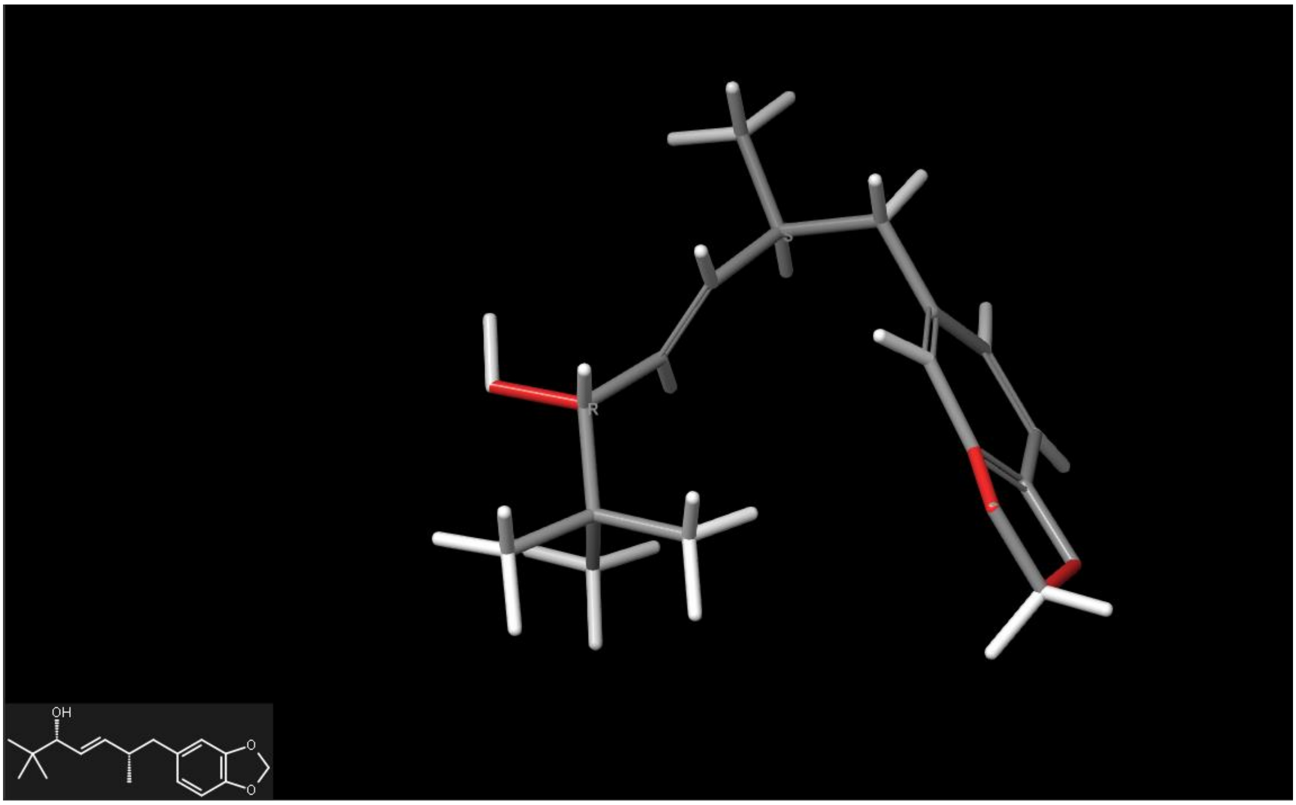

Figure S61. DFT-optimized lowest energy conformer of (2*R*,5*S*)-**4**.

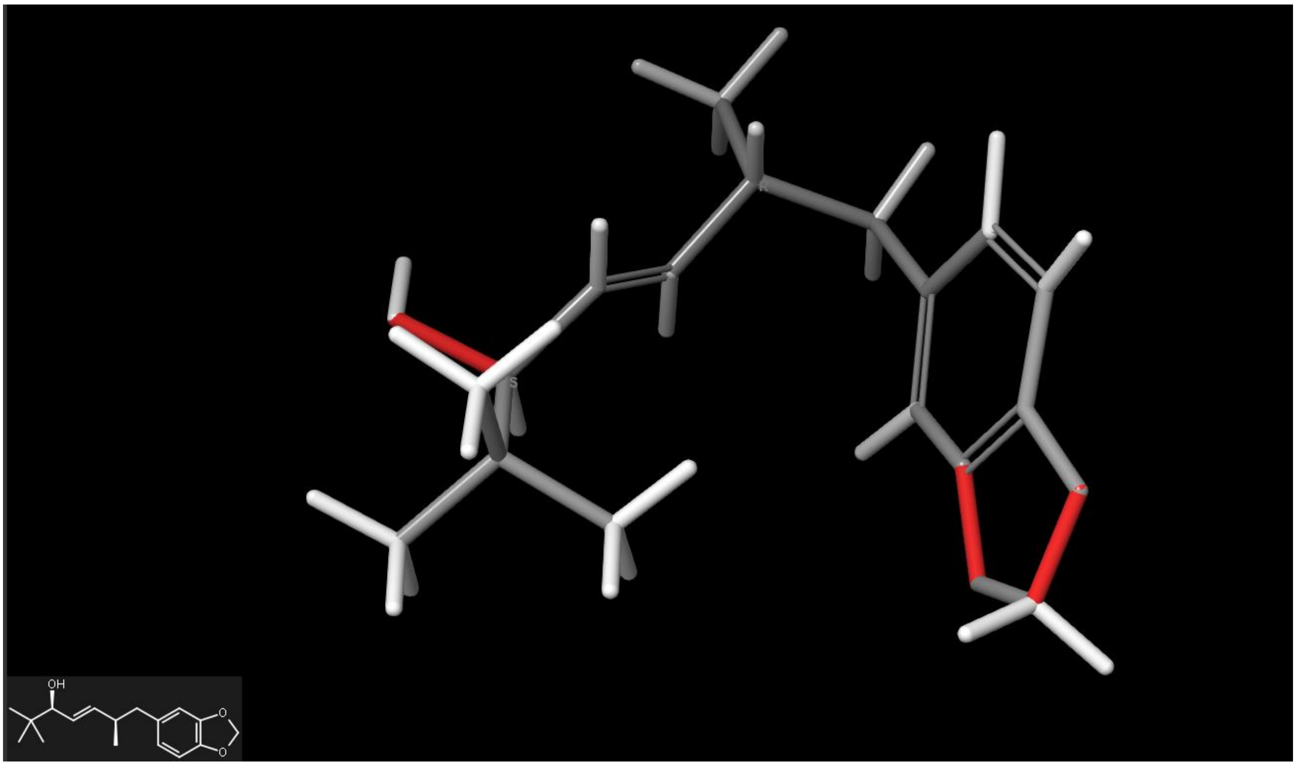

Figure S62. DFT-optimized lowest energy conformer of (2*S*,5*R*)-**4**.

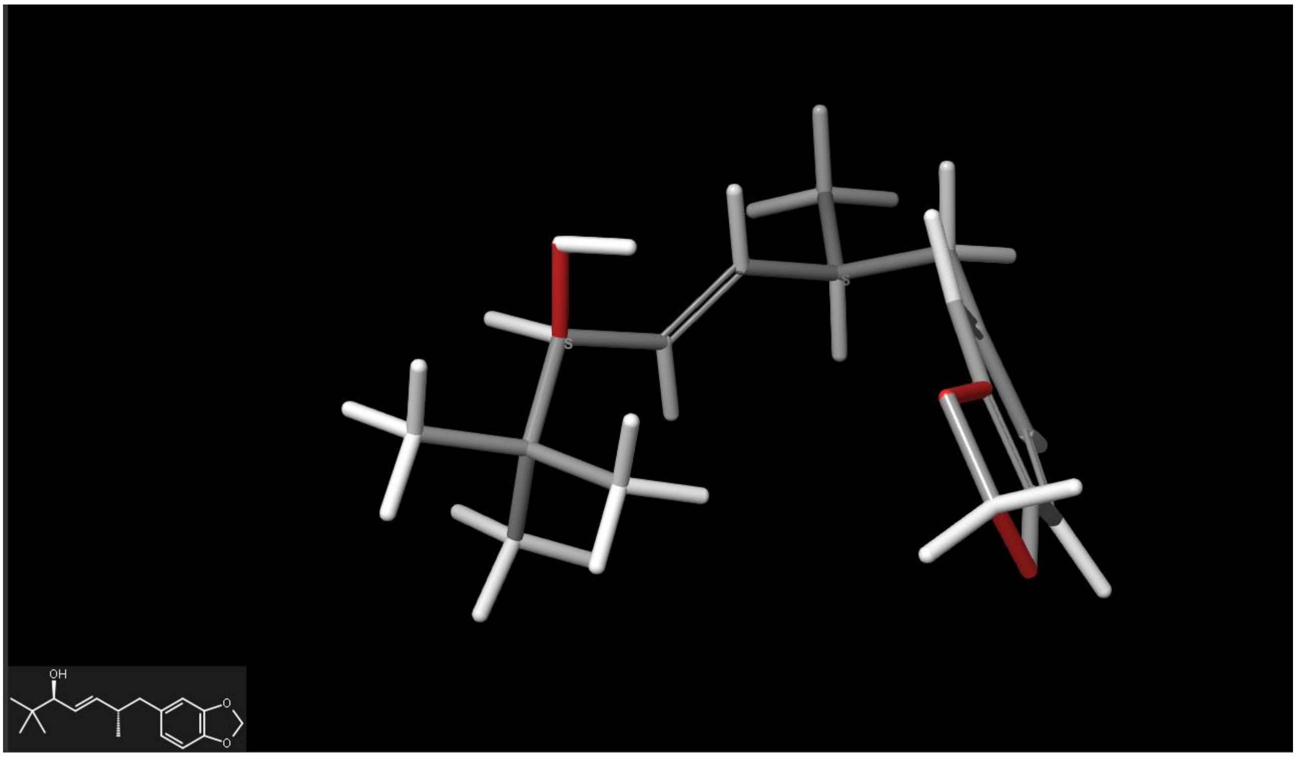

Figure S63. DFT-optimized lowest energy conformer of (2*S*,5*S*)-**4**.

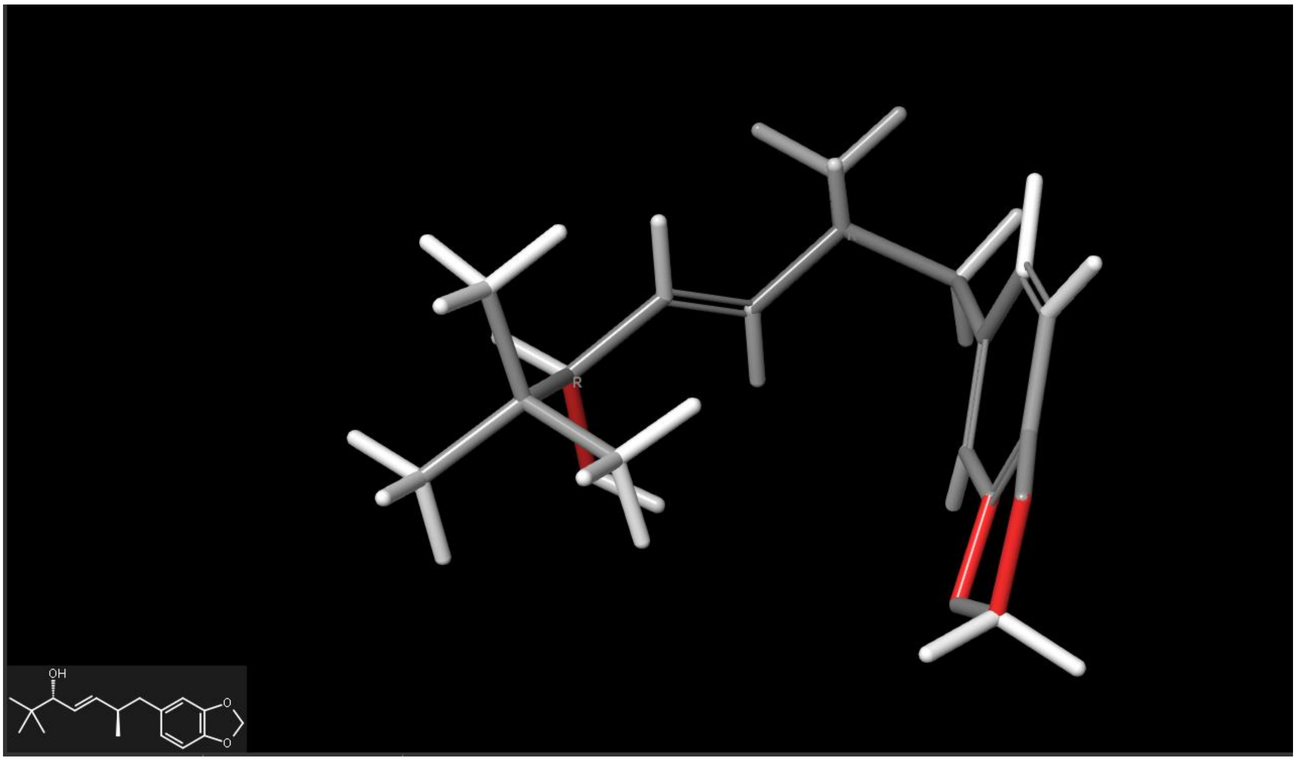

Figure S64. DFT-optimized lowest energy conformer of (2*R*,5*R*)-**4**.

6. **Dose response curves against *h*LDHA**

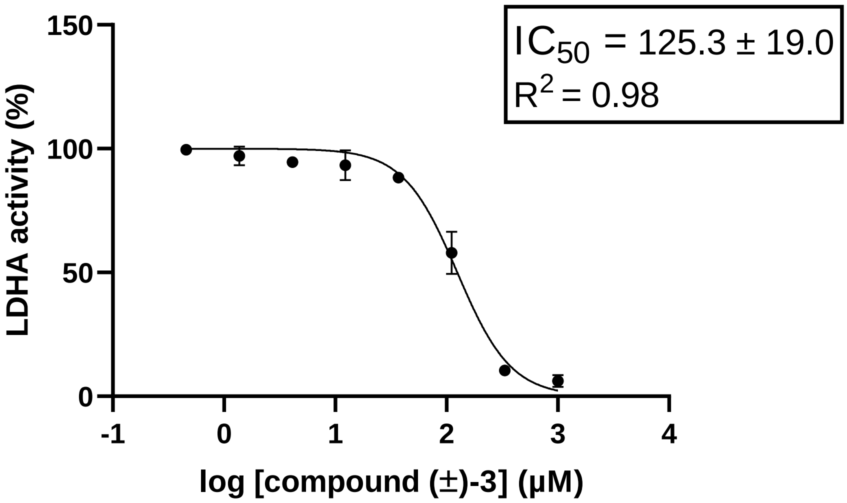

Figure S65. *h*LDHA inhibition curve of compound (±)-**3** (mean ± SD of n = 3 replicates).

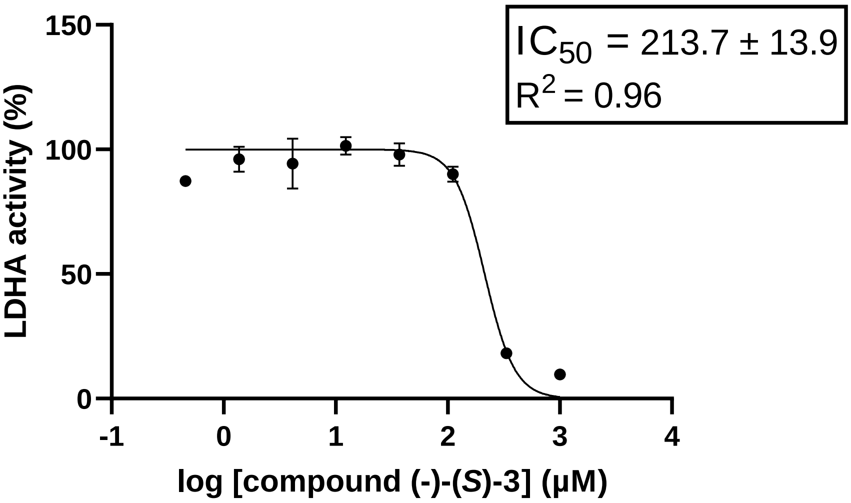

Figure S66. *h*LDHA inhibition curve of compound (–)-(*S*)-**3** (mean ± SD of n = 3 replicates).

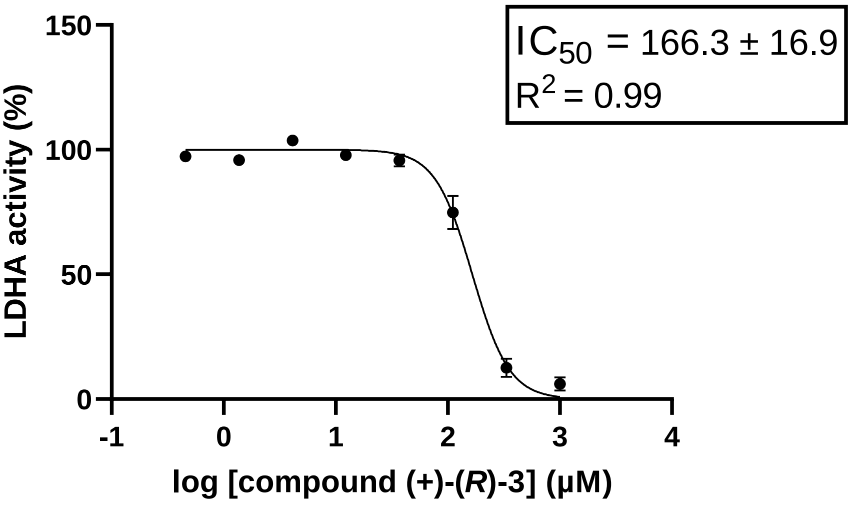

Figure S67. *h*LDHA inhibition curve of compound (+)-(*R*)-**3** (mean ± SD of n = 3 replicates).

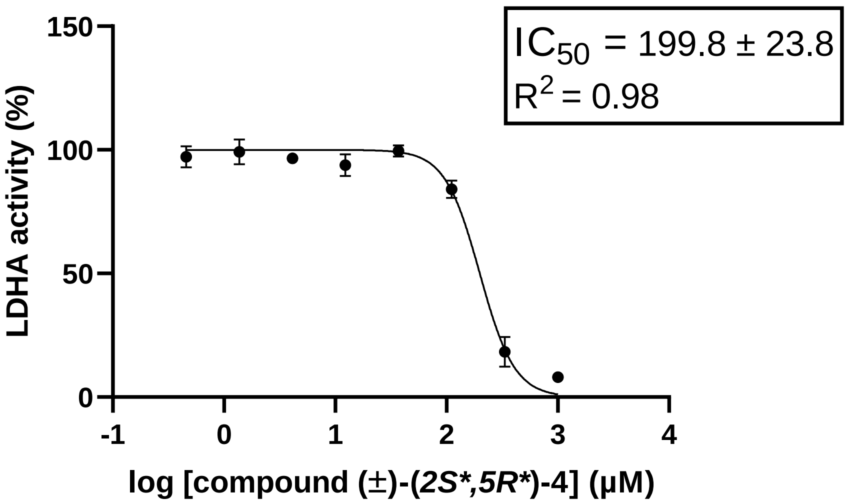

Figure S68. *h*LDHA inhibition curve of compound (±)-(2*S**,5*R**)-**4** (mean ± SD of n = 3 replicates).

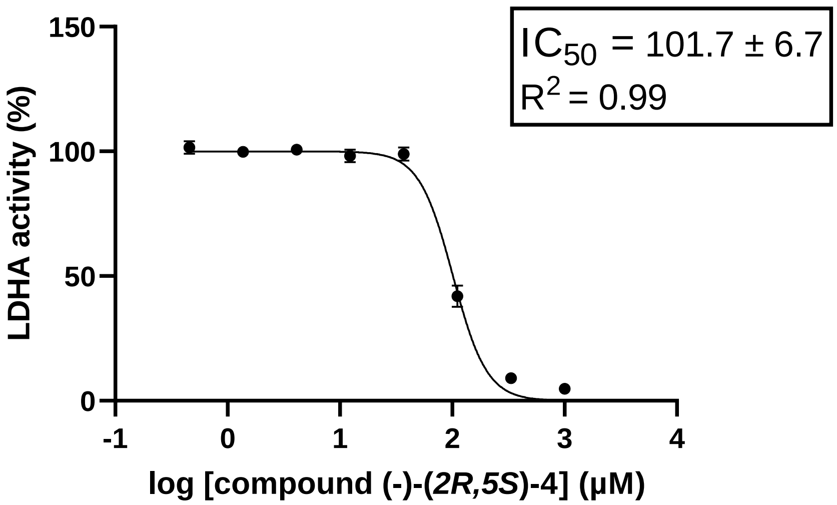

Figure S69. *h*LDHA inhibition curve of compound (–)-(2*R*,5*S*)-**4** (mean ± SD of n = 3 replicates).

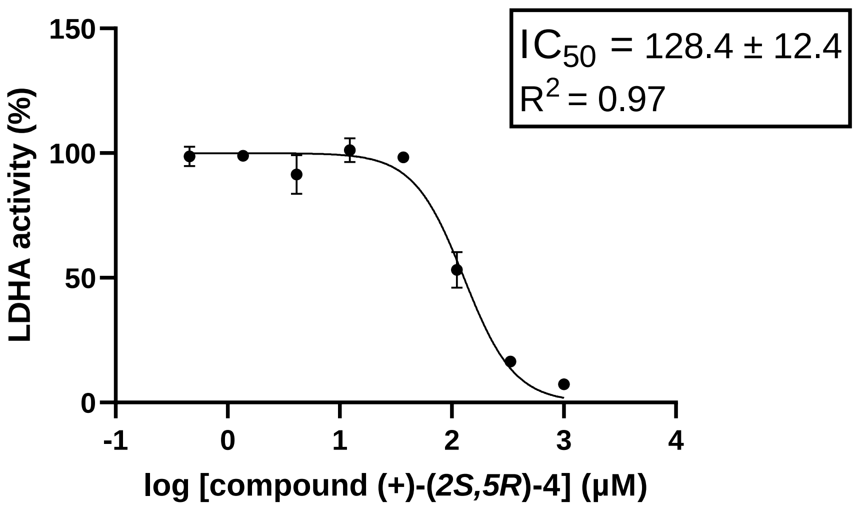

Figure S70. *h*LDHA inhibition curve of compound (+)-(2*S*,5*R*)-**4** (mean ± SD of n = 3 replicates).

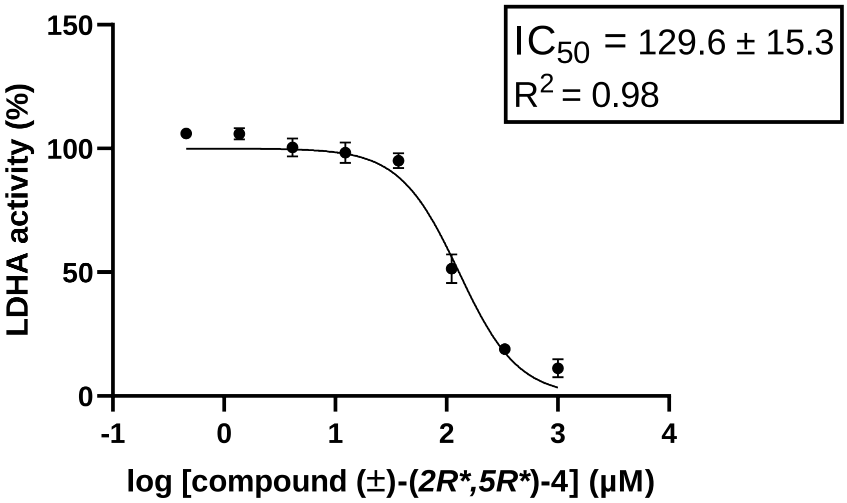

Figure S71. *h*LDHA inhibition curve of compound (±)-(2*R**,5*R**)-**4** (mean ± SD of n = 3 replicates).

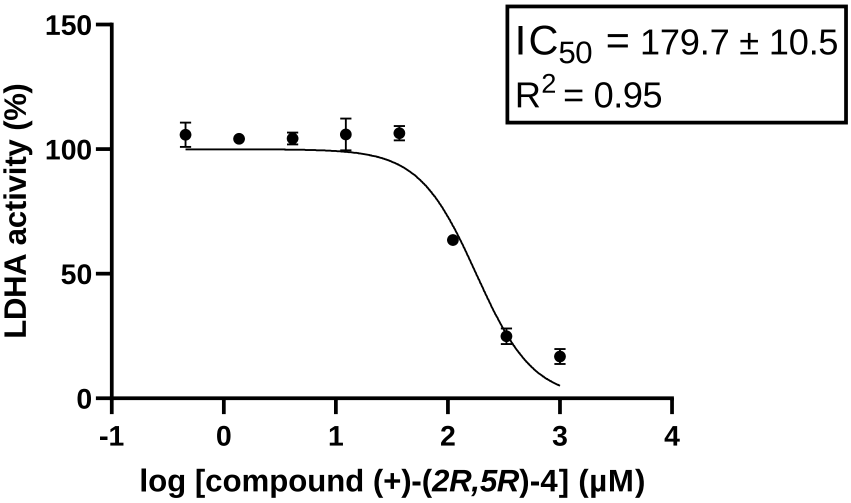

Figure S72. *h*LDHA inhibition curve of compound (+)-(2*R*,5*R*)-**4** (mean ± SD of n = 3 replicates).

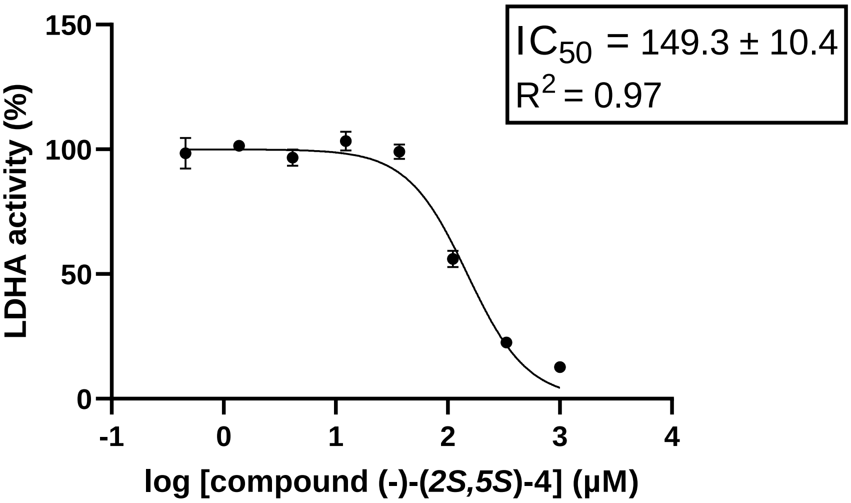

Figure S73. *h*LDHA inhibition curve of compound (–)-(2*S*,5*S*)-**4** (mean ± SD of n = 3 replicates).

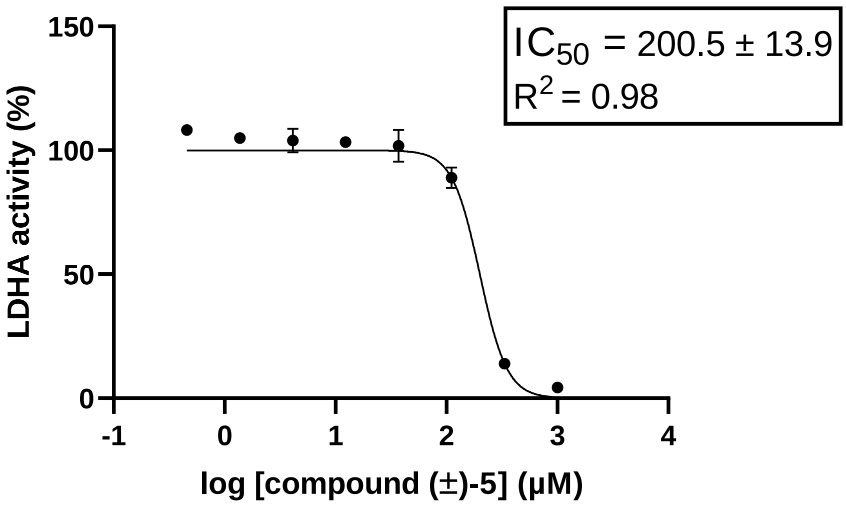

Figure S74. *h*LDHA inhibition curve of compound (±)-**5** (mean ± SD of n = 3 replicates).
